# Molecular imine cages with π-basic Au_3_(pyrazolate) faces†

**DOI:** 10.1039/d3sc06280e

**Published:** 2024-02-01

**Authors:** Noga Eren, Farzaneh Fadaei-Tirani, Rosario Scopelliti, Kay Severin

**Affiliations:** a Institut des Sciences et Ingénierie Chimiques, École Polytechnique Fédérale de Lausanne (EPFL) 1015 Lausanne Switzerland kay.severin@epfl.ch

## Abstract

One tetrahedral and two trigonal prismatic cages with π-basic Au_3_(pyrazolate)_3_ faces were obtained by connection of pre-formed gold complexes *via* dynamic covalent imine chemistry. The parallel arrangement of the Au_3_(pyrazolate)_3_ complexes in the prismatic cages augments the interaction with π-acids, as demonstrated by the encapsulation of polyhalogenated aromatic compounds. The tetrahedral cage was found to act as a potent receptor for fullerenes. The structures of the three cages, as well as the structures of adducts with C_60_ and C_70_, could be established by X-ray crystallography.

## Abstract 2

Molecular cages with π-basic Au_3_(pyrazolate)_3_ faces were obtained by linking pre-formed gold complexes *via* dynamic covalent imine chemistry. The cages are able to encapsulate different guests including fullerenes.

## Graphical abstract

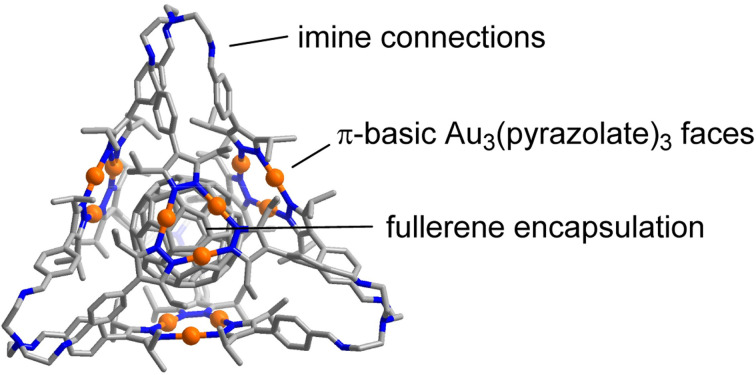

## Introduction

**Fig. 1:**
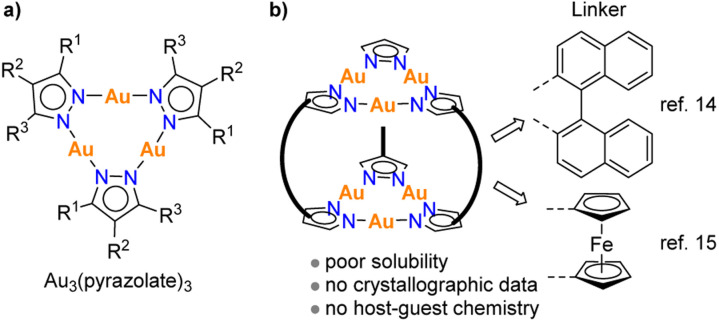
The general structure of trinuclear gold pyrazolate complexes (a), and previously reported hexanuclear gold pyrazolate complexes with binaphthyl or ferrocenyl linkers (b).

Trinuclear gold complexes of the general formula Au_3_(pyrazolate)_3_ (Fig. 1a) were first described by Bonati and co-workers in 1974.^1^ Following this initial report, Au_3_(pyrazolate)_3_ complexes were studied by numerous other groups.^2^ These investigations have shown that Au_3_(pyrazolate)_3_ complexes display high chemical and thermal stability.^2,3^ Similar to other Au(i) complexes, Au_3_(pyrazolate)_3_ trimers are prone to form Au⋯Au contacts in the solid state, and the presence of these aurophilic interactions is often associated with solid-state luminescence.^2,4^ Luminescence can also be induced by the confinement of multiple Au_3_(pyrazolate)_3_ complexes in supramolecular hosts.^5^ An important feature of Au_3_(pyrazolate)_3_ complexes is their variable π-acidity/basicity.^2*c*,6^ Most Au_3_(pyrazolate)_3_ complexes behave as π-bases.^2*c*,7^ However, the trimer Au_3_[(3,5-CF_3_)_2_pz]_3_ was found to be π-acidic due to the presence of electron-withdrawing CF_3_ groups.^8^

Au_3_(pyrazolate)_3_ complexes have found different applications.^2^ For example, they were used to form mesogens,^9^ stimuli-responsive organogels,^10^ or conductive thin films.^11^ Furthermore, a Au_3_(pyrazolate)_3_ complex was employed as a chemosensor for the selective detection of Ag^+^ ions,^12^ and materials based on 2-dimensional nanosheets containing Au_3_(pyrazolate)_3_ complexes were used for photocatalytic hydrogen evolution.^13^

So far, there are few reports about cage-like structures with multiple Au_3_(pyrazolate)_3_ units. Thiel and co-workers have synthesized a hexanuclear Au complex, in which two Au_3_(pyrazolate)_3_ complexes are connected by three binaphthyl spacers (Fig. 1b).^14^ The complex was found to display poor solubility, preventing a solution-based characterization. Structurally related complexes with ferrocenyl linkers were described by Meyer and co-workers.^15^ Again, poor solubility was encountered, hampering a more comprehensive characterization. The limited success in preparing defined complexes with multiple Au_3_(pyrazolate)_3_ units is in contrast to what was found for analogous Cu_3_(pyrazolate)_3_ and Ag_3_(pyrazolate)_3_ complexes. Cu_3_(pyrazolate)_3_ and Ag_3_(pyrazolate)_3_ complexes have been incorporated into prismatic and antiprismatic cages,^16^ and some of these cages were found to encapsulate small molecules.^16*a*–*c*^ Furthermore, there is a report about an octahedral cage containing four Cu_3_(pyrazolate)_3_ complexes,^17^ and studies about bridged^18^ or interlocked systems^19^ with two Cu_3_(pyrazolate)_3_-based prisms.

The difficulty in preparing more complex molecular structures with multiple Au_3_(pyrazolate)_3_ units is likely related to two features of Au_3_(pyrazolate)_3_ complexes. First, metallophilic interactions are stronger for Au_3_(pyrazolate)_3_ complexes than for analog Cu_3_(pyrazolate)_3_ and Ag_3_(pyrazolate)_3_ complexes.^2^ Stronger intermolecular interaction can lead to reduced solubility. Second, Au_3_(pyrazolate)_3_ complexes are rather inert.^20^ As a result, error correction processes are less efficient during metallosupramolecular syntheses. Notwithstanding these difficulties, we think that molecularly defined nanostructures with multiple Au_3_(pyrazolate)_3_ units are worthwhile synthetic targets, because the pronounced π-basicity of Au_3_(pyrazolate)_3_ complexes is expected to lead to interesting host properties.

Below, we describe examples of well-soluble molecular cages containing two or four Au_3_(pyrazolate)_3_ faces. The cages were obtained by connection of pre-formed gold complexes *via* dynamic covalent imine chemistry. The presence of the Au complexes enables the molecular recognition of different guest molecules. Notably, a tetrahedral cage was found to be a potent receptor for C_60_ and C_70_.

## Results and discussion

**Scheme 1:**
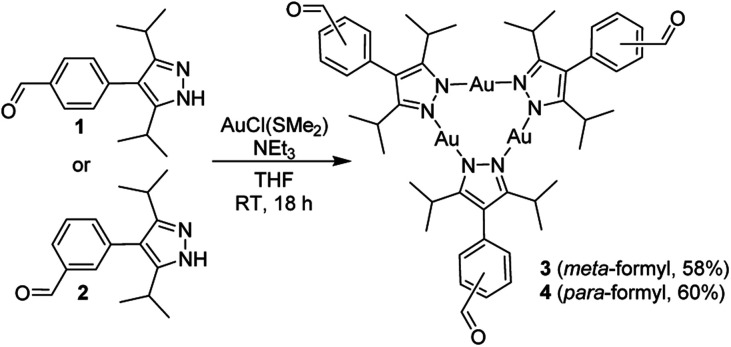
Synthesis of the complexes 3 and 4.

The substituted pyrazoles 1 and 2 (Scheme 1) were obtained by Suzuki cross-coupling reactions of 4-bromo-3,5-diisopropyl-1-tosyl-1*H*-pyrazole with the corresponding formylphenylboronic acids, followed by base-induced deprotection (for details, see the ESI†). Subsequent reactions with AuCl(SMe_2_) in the presence of triethylamine in THF gave the Au_3_(pyrazolate)_3_ complexes 3 and 4 (Scheme 1). Both complexes are soluble in chloroform, but they display very poor solubility in acetonitrile and diethyl ether.

**Fig. 2:**
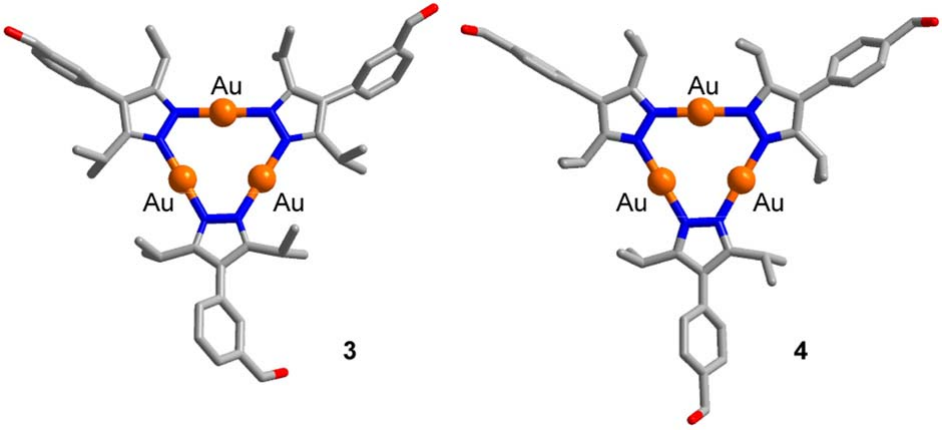
Graphic representation of the molecular structures of 3 and 4 as determined by single-crystal XRD. Hydrogen atoms are not shown.

The trinuclear complexes 3 and 4 were characterized by NMR spectroscopy and single-crystal X-ray diffraction (XRD). The XRD analyses (Fig. 2) confirm that trinuclear complexes have formed.^21^ In the solid state, 3 and 4 display a co-planar arrangement of the pyrazolate heterocycles, and the Au–N bond distances are within the expected range (1.98 to 2.02 Å). Close intermolecular Au⋯Au contacts are not observed.

Organic cages with imine linkages can be obtained in condensation reaction of di/poly-amines with di/poly-aldehydes.^22^ The trinuclear complexes 3 and 4 appeared to be potentially well-suited for such condensation reactions.^23^ However, the clean formation of imine cages is often not straightforward, even if the building blocks seem to have an appropriate geometry. Frequently encountered problems include incomplete condensation reactions, the formation of side products (insoluble polymers or mixtures of cages), and structural rearrangements during isolation.^24^ In the following, we focus on reactions that resulted in the clean formation of a structurally defined cage. A brief discussion of reaction with other amines can be found in the ESI.†

**Scheme 2:**
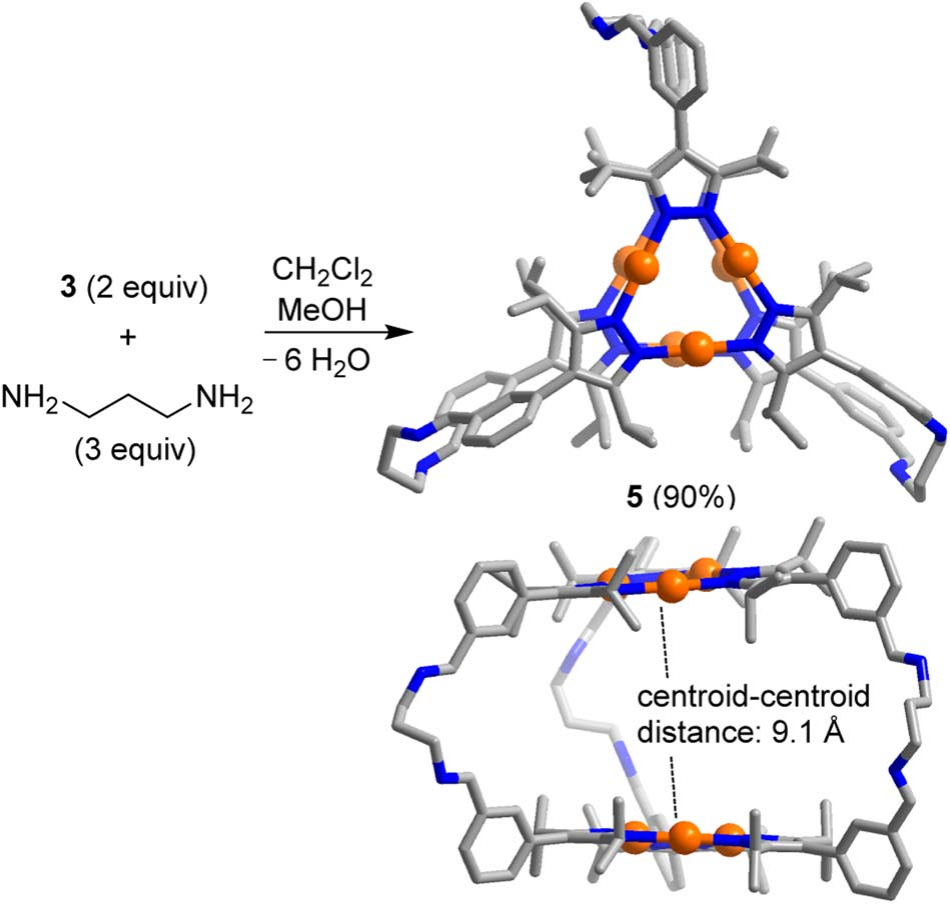
Synthesis of the prismatic cage 5. The graphic representation of the product is based on a crystallographic analysis (view from the top and from the side; hydrogen atoms are not shown).

The reaction of complex 3 (2 equiv.) with 1,3-diaminopropane (3 equiv.) in a mixture of dichloromethane and methanol (3 : 2) gave the [2 + 3] condensation product 5 in high yield (Scheme 2). In solution, cage 5 displays high apparent symmetry, with only one set of NMR signals for the six bridging pyrazolate groups.

Single crystals of 5, suitable for an XRD analysis, were obtained by layering of acetonitrile onto a solution of 5 in dichloromethane. The two Au_3_(pyrazolate)_3_ complexes in the trigonal prismatic cage 5 are arranged in a parallel fashion, with a distance between the planes of ∼9 Å and a distance between the centroids of 9.1 Å. This spacing suggests that 5 is potentially well-suited to bind ‘flat’ aromatic π-systems.^2*b*^ The two Au trimers are roughly eclipsed with an angle of ∼6° between them.

**Scheme 3:**
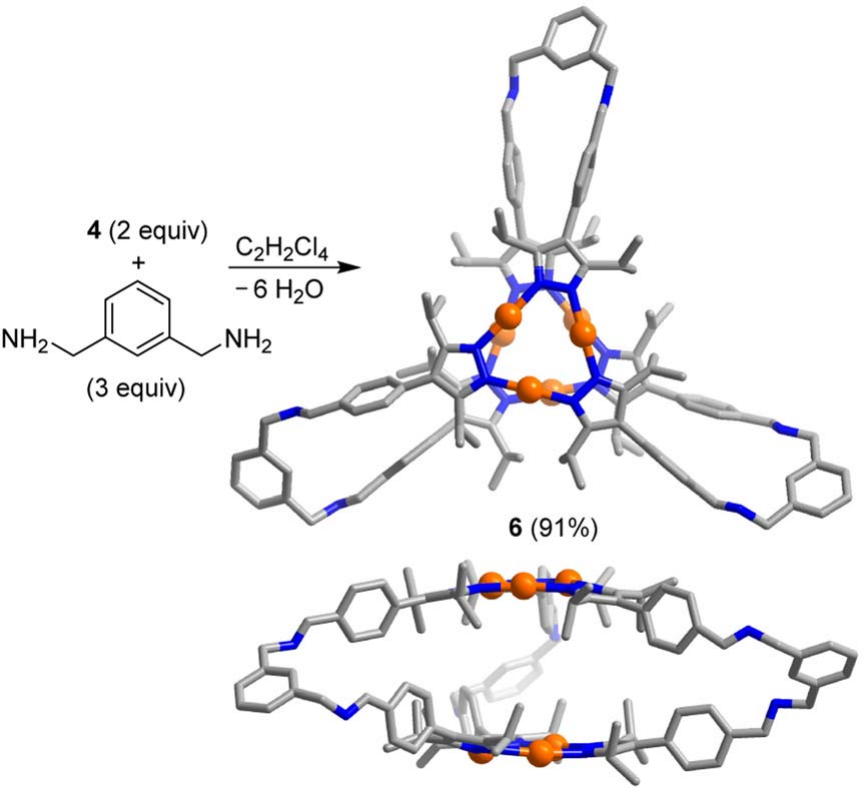
Synthesis of the prismatic cage 6. The graphic representation of the product is based on a crystallographic analysis (view from the top and from the side; hydrogen atoms are not shown).

The trigonal prismatic cage 6 was obtained in high yield by combining the Au trimer 4 with *m*-xylylenediamine in a ratio of 2 : 3 in C_2_H_2_Cl_4_ (Scheme 3). An XRD analysis of 6 revealed that the height of the prismatic cage, as defined by the distance between the planes of the Au trimers, is ∼7 Å. The overall size of prismatic 6 is significantly larger than that of 5, with a maximum C⋯C distance of 26.8 Å (5: 20.4 Å). In contrast to what was found for 5, one can observe short intermolecular Au⋯Au contacts in crystalline 6 (for details, see the ESI†). The deviation of the Au trimers from a staggered arrangement is ∼25°.

**Scheme 4:**
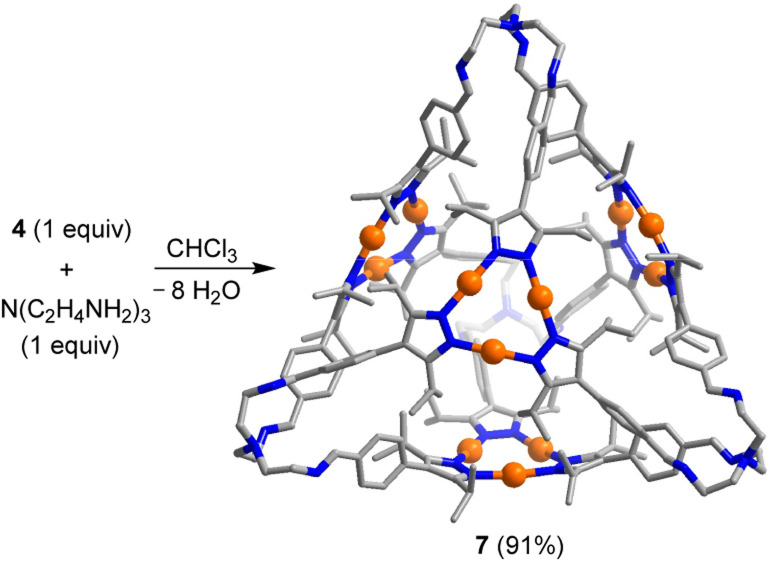
Synthesis of the tetrahedral cage 7. The graphic representation of the product is based on a crystallographic analysis. Hydrogen atoms are not shown.

Tris(2-aminoethyl)amine (TREN) is frequently employed as a building block for the synthesis of imine-based organic cages.^22*a*^ A mixture of TREN and the Au trimer 4 (ratio: 1 : 1) in CDCl_3_ gave the [4 + 4] cage 7 (Scheme 4) in nearly quantitative yield as revealed by *in situ* NMR spectroscopy and ESI mass spectrometry analysis of the reaction mixture. Isolation of 7 was possible by precipitation with acetonitrile (yield: 91%).

At room temperature, the ^1^H NMR spectrum of 7 in CDCl_3_ showed very broad peaks for the CH signals of the phenylene groups. When cooling the solution to 273 K, four defined signals for the aromatic CH protons were observed. The underlying dynamic phenomenon is likely a hindered rotation of the tightly packed phenylene groups.^25^ Another noteworthy spectroscopic feature is the presence of two sets of NMR signals for iso-propyl substituents at the pyrazolate ligands. The appearance of two sets of signals is a consequence of the chirality of the TREN-based vertices,^26^ rending the iso-propyl groups diastereotopic.

A crystallographic analysis of 7 confirmed the tetrahedral shape of the cage (Scheme 4). The edge length of 7 is 24.8 Å (maximum C⋯C distance), making it one of the largest TREN-based imine cages described so far.^22*a*^ The Au_3_(pyrazolate)_3_ complexes panel the four faces of the tetrahedron. As deduced by NMR spectroscopy, the TREN-based vertices show a propeller-like conformation, with the same helical orientation for all four vertices. Residual electron density pointed to the presence of disordered solvent molecules. A solvent mask was calculated, and 2010 electrons were found in a volume of 7652 Å^3^ in two voids per unit cell.^27^ The solvent molecules, too disordered to be located in the electron density map, were taken into account using the Olex2 solvent-mask procedure.^28^

**Fig. 3:**
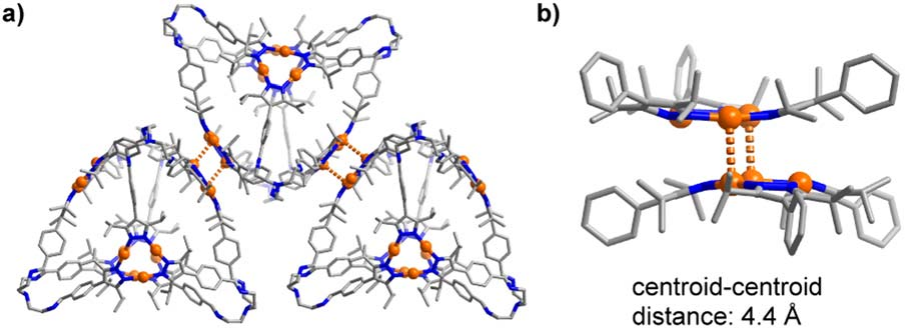
Aurophilic interactions between cages in crystalline 7 (a), and zoom-in on two Au_3_(pyrazolate)_3_ complexes in 7, highlighting the bent geometry of the trimers (b). Hydrogen atoms are not shown.

In crystalline 7, close Au⋯Au contacts between the cages are observed (Fig. 3a). The corresponding Au⋯Au distances range from 3.268 to 3.393 Å. These aurophilic interactions are present for three out of the four Au_3_(pyrazolate)_3_ complexes in cage 7. To accommodate the Au⋯Au contacts, the Au_3_(pyrazolate)_3_ complexes adopt a bent geometry (Fig. 3b), resulting in tetrahedral cages with slightly convex faces. A similar bending of the Au_3_(pyrazolate)_3_ complexes was observed for the prismatic cage 6 (Scheme 3, graphic on the bottom). As discussed, this cage shows likewise intermolecular Au⋯Au contacts in the solid state.

**Scheme 5:**
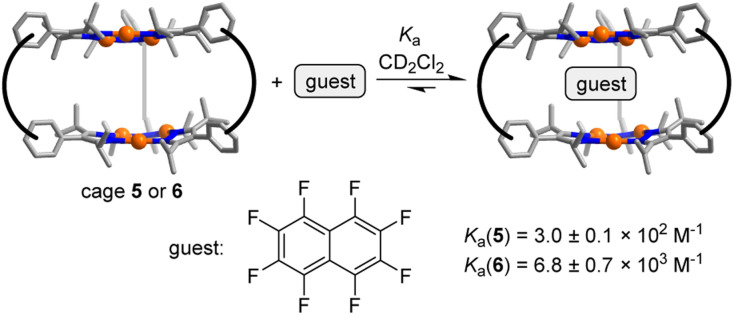
Molecular recognition of octafluoronaphthalene.

The arrangement of the π-basic Au_3_(pyrazolate)_3_ complexes in 5 and 6 suggested that the prismatic cages might be able to act as hosts for π-acidic aromatic compounds. This proposition could be corroborated by NMR studies. The addition of increasing amounts of octafluoronaphthalene to a solution of cage 5 in CD_2_Cl_2_ resulted in complexation-induced shifts (CIS) of the ^1^H NMR signals (for details, see the ESI†). The CIS *vs.* concentration data could be fitted to a 1 : 1 binding model resulting in an apparent association constant of *K*_a_ = 3.0 ± 0.1 × 10^2^ M^−1^ (Fig. S54†) (Scheme 5).

For cage 6, the complexation of octafluoronaphthalene was found to be slow on the ^19^F NMR time scale, and separate signals for the ‘free’ and the ‘bound’ guest were observed. By integration of the ^19^F NMR signals, we were able to derive a binding constant of *K*_a_ = 6.8 ± 0.7 × 10^3^ M^−1^ (Fig. S50†). A similar value was derived from ^1^H NMR data (for details, see the ESI, Fig. S48 and S49†). The tighter binding of octafluoronaphthalene by cage 6 is possibly related to the reduced flexibility of the *m*-xylylene linkers when compared to the propylene linkers in cage 5. Hexabromobenzene and hexachlorobenzene are likewise bound by 5 and 6, as evidenced by NMR experiments (for details, see the ESI†). The parallel arrangement of the two Au_3_(pyrazolate)_3_ complexes in 5 and 6 is of key importance for the complexation of polyhalogenated compounds. In control experiments with the simple Au_3_(pyrazolate)_3_ complexes 3 and 4, we were not able to detect an interaction with polyhalogenated compounds.^29^ Likewise, we were not able to observe a complexation of octafluoronaphthalene by cage 7.

Coinage metal pyrazolate complexes of the general formula [M{3,5-(CF_3_)_2_pz}]_3_ (M = Cu, Ag, Au) were reported to form co-crystals with C_60_, with four [M{3,5-(CF_3_)_2_pz}]_3_ complexes surrounding C_60_ in a tetrahedral fashion.^30^ This finding inspired us to examine if cage 7 would be able to bind fullerenes.^31^

When C_60_ (2 equiv.) was added to a solution of cage 7 (1.7 mM) in C_2_D_2_Cl_4_, the formation of the host–guest complex C_60_⊂7 was detected by mass spectrometry. The complexation of C_60_ was found to be slow on the NMR time scale, and it could be monitored by ^1^H NMR spectroscopy. Quantitative formation of C_60_⊂7 was observed within 12 h. Similar results were obtained with C_70_. Upon mixing C_70_ and cage 7 in C_2_D_2_Cl_4_, the adduct C_70_⊂7 formed in quantitative yield as evidenced by ^1^H NMR spectroscopy and mass spectrometry. In competition experiments with equal amounts of C_60_ and C_70_, we observed the formation of the adducts C_60_⊂7 and C_70_⊂7 in nearly equal amounts (∼10 : 8).

**Fig. 4:**
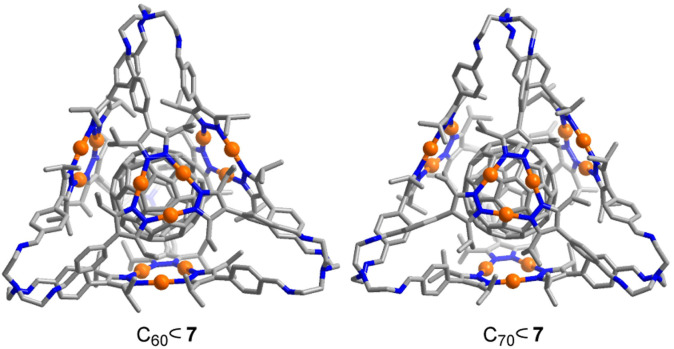
Molecular structure of C_60_⊂7 and C_60_⊂7 as determined by single-crystal XRD. Hydrogen atoms are not shown.

The structures of C_60_⊂7 and C_70_⊂7 in the solid state were analyzed by single-crystal XRD. As expected, the fullerenes are bound in the central cavity of cage 7 (Fig. 4). The distance between the fullerenes and the Au_3_(pyrazolate)_3_ complexes, as defined by the closest Au⋯C contacts, is ∼3.4 Å. The average distance between the centroids of the four Au trimers in C_60_⊂7 is 10.7 Å. For C_70_⊂7, the corresponding value is 11.3 Å and for 7 it is 11.8 Å. Intermolecular Au⋯Au contacts, as observed for the ‘empty’ cage 7, are observed for C_70_⊂7 but not for C_60_⊂7 (for details, see the ESI†).

## Conclusions

Molecular cages containing Au_3_(pyrazolate)_3_ complexes were obtained by connection of pre-formed gold complexes *via* dynamic covalent imine chemistry. In contrast to previously described cages with Au_3_(pyrazolate)_3_ faces,^14,15^ the imine cages are soluble in chlorinated organic solvents. It was therefore possible to perform solution-based analyses and to grow single crystals for XRD measurements. The parallel arrangement of the two Au_3_(pyrazolate)_3_ complexes in the trigonal prismatic cages 5 and 6 enabled the complexation of polyhalogenated aromatic compounds. Notably, we observed tight encapsulation of octafluoronaphthalene by cage 6 with an apparent binding constant of *K*_a_ = 6.8 ± 0.7 × 10^3^ M^−1^. The tetrahedral cage 7 is able to form adducts with C_60_ and C_70_ in a competitive solvent such as tetrachloroethane. Overall, our results provide evidence that the incorporation of Au_3_(pyrazolate)_3_ complexes in molecularly defined nanostructures can give compounds with interesting host–guest chemistry.

## Data availability

The data that support the findings of this study are available in the ESI† of this article.

## Author contributions

N. E. and K. S. initiated the study, N. E. performed the experiments and analyzed the data, F. F.-T. and R. S. collected and processed the X-ray data, and N. E. and K. S. co-wrote the manuscript. All authors discussed the results and commented on the manuscript.

## Conflicts of interest

There are no conflicts to declare.

## Supplementary Material

SC-015-D3SC06280E-s001

SC-015-D3SC06280E-s002
