# Supplementary material for: Molecular imine cages with π-basic Au3(pyrazolate) faces
Source: Chem Sci. 2024 Feb 1;15(10):3539–44. doi: 10.1039/d3sc06280e (PMC10915823; doi:10.1039/d3sc06280e)
Supplement: SC-015-D3SC06280E-s001 [file SC-015-D3SC06280E-s001.pdf]

Electronic Supplementary Material (ESI) for Chemical Science.
This journal is © The Royal Society of Chemistry 2024

# Electronic Supplementary Information

## Molecular imine cages with π-basic $Au_3(pyrazolate)_3$ faces

Noga Eren,[a] Farzaneh Fadaei-Tirani,[a] Rosario Scopelliti,[a] and Kay Severin*[a]

[a] Institut des Sciences et Ingénierie Chimiques, Ecole Polytechnique Fédérale de Lausanne (EPFL), 1015 Lausanne, Switzerland. E-mail: kay.severin@epfl.ch

# Table of contents


1 General ........ 3
2 Syntheses ........ 4
2.1 Synthesis of complex 3 ........ 4
2.2 Synthesis of complex 4 ........ 9
2.3 Synthesis of cage 5 ........ 14
2.4 Synthesis of cage 6 ........ 17
2.5 Synthesis of cage 7 ........ 21
2.6 Screening of different condensation reactions ........ 25
3 Host-guest chemistry ........ 26
3.1 $C_{60}$⊂7 ........ 26
3.2 $C_{70}$⊂7 ........ 30
3.3 Competition experiment ........ 34
3.4 Host-guest chemistry with the cages 5 and 6 ........ 35
4 Crystallographic Data ........ 40
4.1 Crystal structure of 3 ........ 40
4.2 Crystal structure of 4 ........ 42
4.3 Crystal structure of 5 ........ 45
4.4 Crystal structure of 6 ........ 48
4.5 Crystal structure of 7 ........ 51
4.6 Crystal structure of $C_{60}$⊂7 ........ 54
4.7 Crystal structure of $C_{70}$⊂7 ........ 57
5 References ........ 60

# 1 General

All reagents were obtained from commercial sources and used without further purification unless stated otherwise. 4-Bromo-3,5-diisopropyl-2-(p-toluenesulfonyl)pyrazole was prepared according to a literature procedure.[1] The ligands **1** and **2**, as well as the complexes **3** and **4**, were synthesized in analogy to reported procedures.[1,2]

NMR spectra were measured on a Bruker Avance II spectrometer ($^{1}$H: 800 MHz, $^{13}$C: 200 MHz) equipped with a CPTCl$_{xyz}$ 5 mm cryoprobe, Bruker Avance III HD spectrometer ($^{1}$H: 600 MHz, $^{13}$C: 150 MHz) equipped with a CPPBBO$_{z}$ 5 mm probe a Bruker Avance III spectrometer ($^{1}$H: 400 MHz, $^{13}$C: 100 MHz, $^{19}$F: 376 MHz) equipped with a BBFO$_{z}$ 5 mm probe and a Bruker Avance III spectrometer ($^{1}$H: 400 MHz, $^{13}$C: 100 MHz) equipped with a Prodigy BBO 5 mm cryoprobe. The chemical shifts are reported in part per million (ppm) using the solvent residual signal as a reference.

Mass spectrometry analyses were performed on a LTQ Orbitrap FTMS instrument (LTQ Orbitrap Elite FTMS, Thermo Scientific, Bremen, Germany) operated in the positive mode coupled to HESI-II probe in an Ion Max ion Source. Samples were injected within an infusion rate of 5 µL/min. The experimental conditions for the ionization voltage was –1.4kV whereas the temperature of ion transfer capillary was 80 °C. FTMS spectra were obtained using the high mass range between 200–3000 *m/z* range in the reduce profile mode with a resolution set to 120 K. In all spectra 10 microscan was acquired with a maximum injection time value of 1000 ms.

# 2 Syntheses

## 2.1 Synthesis of complex 3

4-(*o*-Formylphenyl)-3,5-diisopropyl-1-(*p*-toluenesulfonyl)-pyrazole (**1Ts**)

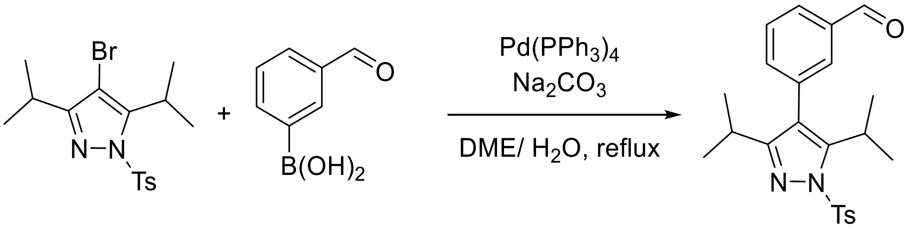


**1Ts**

**Scheme S1**. Synthesis of precursor **1Ts**.

4-Bromo-3,5-diisopropyl-1-(p-toluenesulfonyl)pyrazole (771 mg, 2 mmol, 1 eq.), 3-formylphenylboronic acid (904 mg, 6.03 mmol, 3.02 eq.), and $Pd(PPh_3)_4$ (196 mg, 170 µmol, 0.085 eq.) were dissolved in DME (25 mL) under an atmosphere of $N_2$. Saturated aqueous $Na_2CO_3$ (10 mL) was added and the mixture was heated under reflux for 22 h. After cooling to RT, the product was extracted with DCM (3 × 30 mL). The organic phase was washed with water, saturated $NaHCO_3$, and brine, dried over $MgSO_4$, and evaporated to dryness. The product was purified by column chromatography on silica gel with DCM as eluent. Yield: 589 mg, 72%. $^1$H NMR (400 MHz, $CDCl_3$) δ 10.02 (s, 1H), 7.93 – 7.83 (m, 3H), 7.66 (t, *J* = 1.7 Hz, 1H), 7.55 (t, *J* = 7.6 Hz, 1H), 7.44 (dt, *J* = 7.6, 1.5 Hz, 1H), 7.33 (d, *J* = 7.9 Hz, 2H), 3.82 (hept, *J* = 7.1 Hz, 1H), 2.62 (hept, *J* = 6.9 Hz, 1H), 2.45 (s, 3H), 1.07 (d, *J* = 6.9 Hz, 6H), 1.02 (d, *J* = 7.1 Hz, 6H). $^{13}$C NMR (101 MHz, $CDCl_3$) δ 191.97, 161.02, 150.39, 145.12, 136.88, 136.33, 135.68, 134.49, 131.55, 129.73, 129.35, 128.95, 127.87, 121.05, 26.70, 26.22, 22.42, 21.73, 21.65. ESI-MS: *m*/*z* calculated for $C_{23}H_{26}N_2NaO_3S^+$ $[M+Na]^+$ 433.1556, found 433.1558.

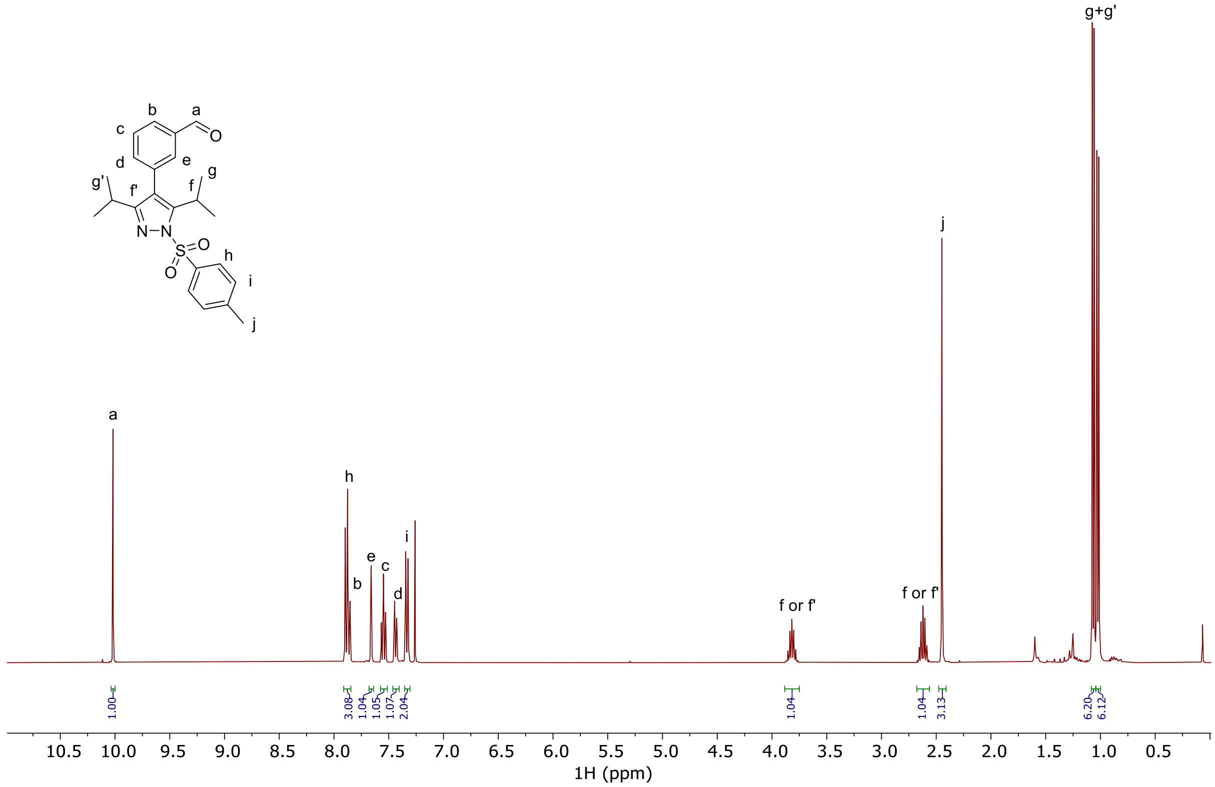


**Figure S1.** $^{1}$H NMR spectrum (400 MHz, $CDCl_3$, 298 K) of **1Ts**.

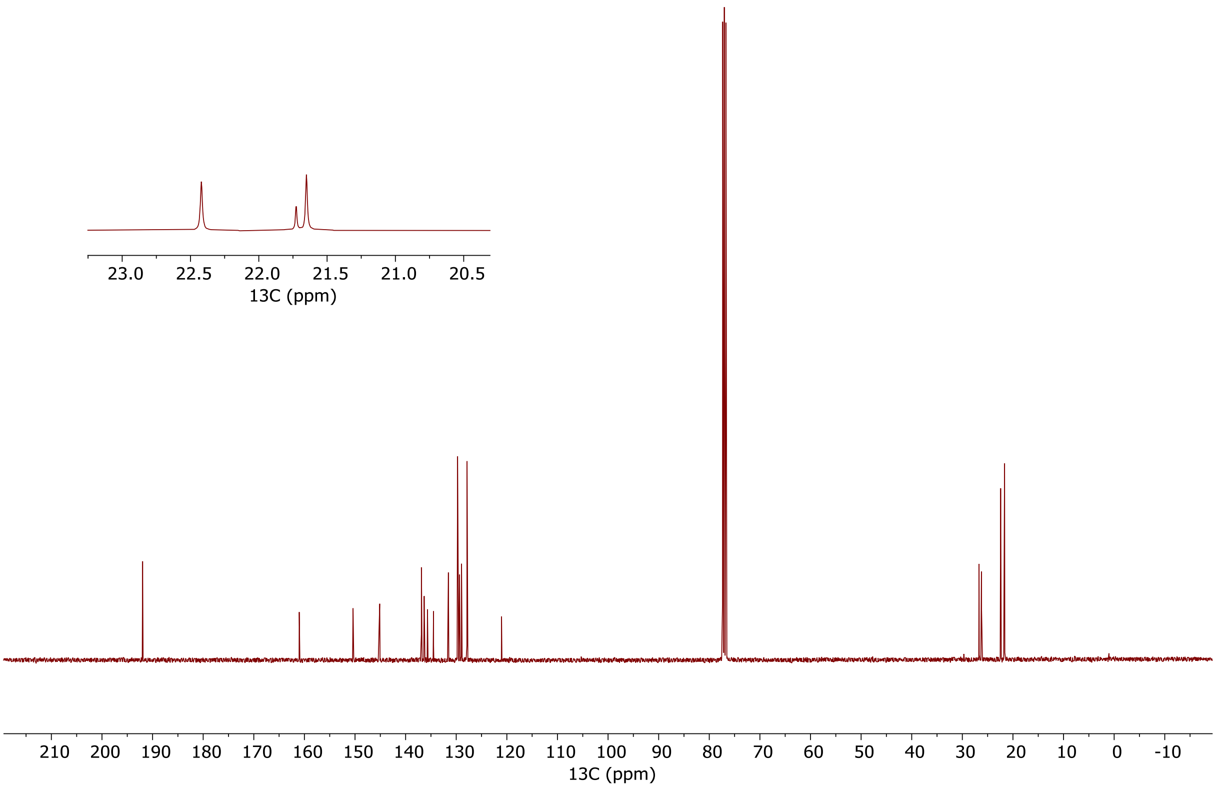


**Figure S2.** $^{13}$C NMR spectrum (101 MHz, $CDCl_3$, 298 K) of **1Ts**.

4-(*o*-Formylphenyl)-3,5-diisopropyl-1H-pyrazole (**1**)

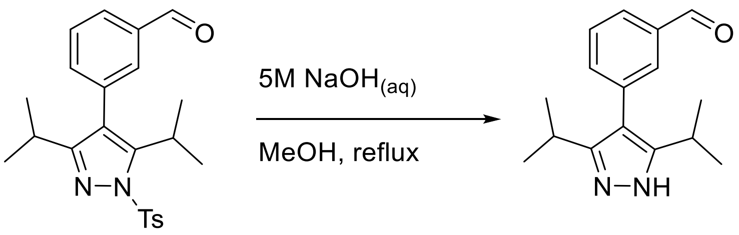

**1**

**Scheme S2.** Synthesis of ligand **1**.

**1Ts** (585 mg, 1.42 mmol) was dissolved in MeOH (18 mL) and a solution of 5M NaOH in $H_2O$ (9 mL) was added. The mixture was heated under reflux for 5 h. After cooling to RT, the product was extracted with DCM (3 × 20 mL). The organic phase was washed with brine, dried over $MgSO_4$, and evaporated to dryness. The product was purified by column chromatography on silica gel with 1% MeOH in DCM as eluent. Yield: 338 mg, 92%. $^1$H NMR (400 MHz, $CDCl_3$) δ 10.06 (s,1H), 7.84 (dt, *J* = 7.5, 1.6 Hz, 1H), 7.79 – 7.73 (m, 1H), 7.58 (t, *J* = 7.5 Hz, 1H), 7.52 (dt, *J* = 7.6, 1.6 Hz, 1H), 2.98 (hept, *J* = 7.0 Hz, 2H), 1.21 (d, *J* = 7.0 Hz, 12H). $^{13}$C NMR (101 MHz, $CDCl_3$) δ 192.39, 151.71, 136.65, 136.32, 135.57, 131.25, 129.13, 128.11, 114.97, 25.52, 22.46. ESI-MS: *m*/*z* calculated for $C_{16}H_{21}N_2O^+$ $[M+H]^+$ 257.1648, found 257.1649.

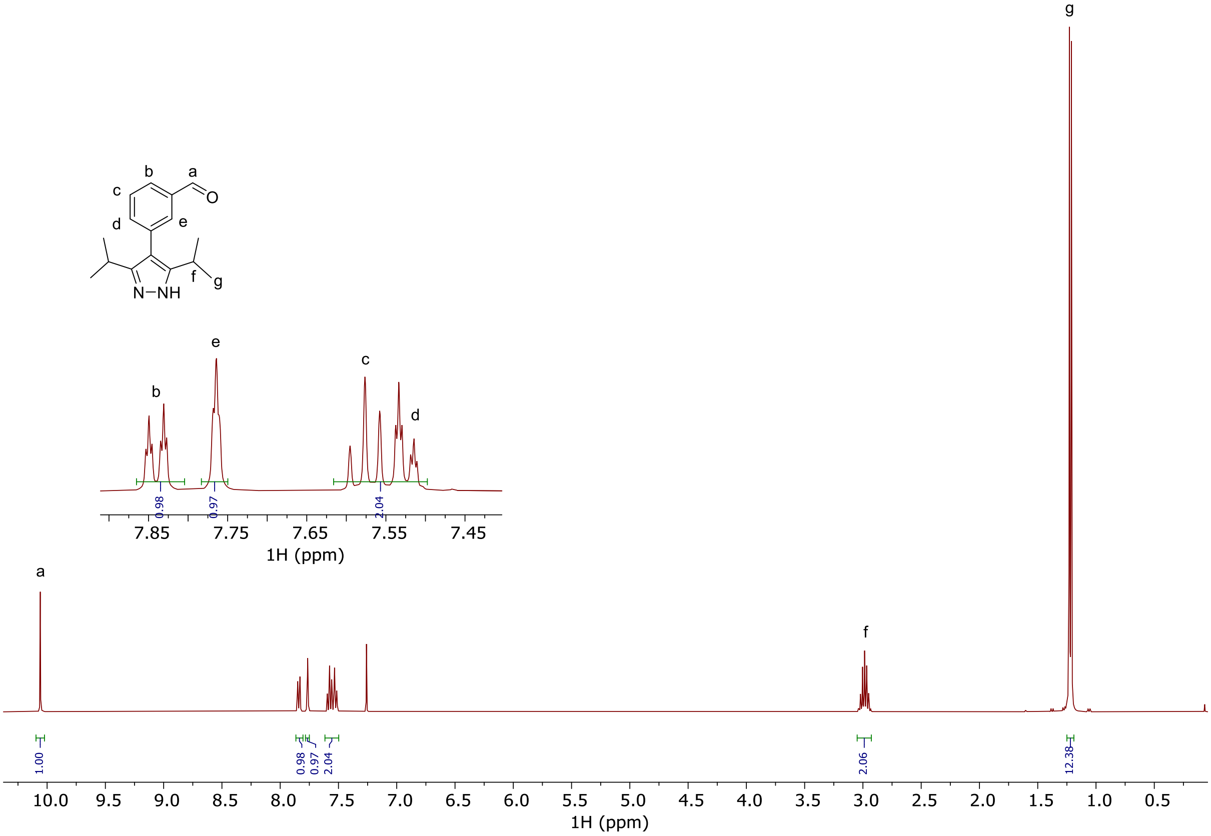


**Figure S3.** $^1$H NMR spectrum (400 MHz, $CDCl_3$, 298 K) of ligand **1.**

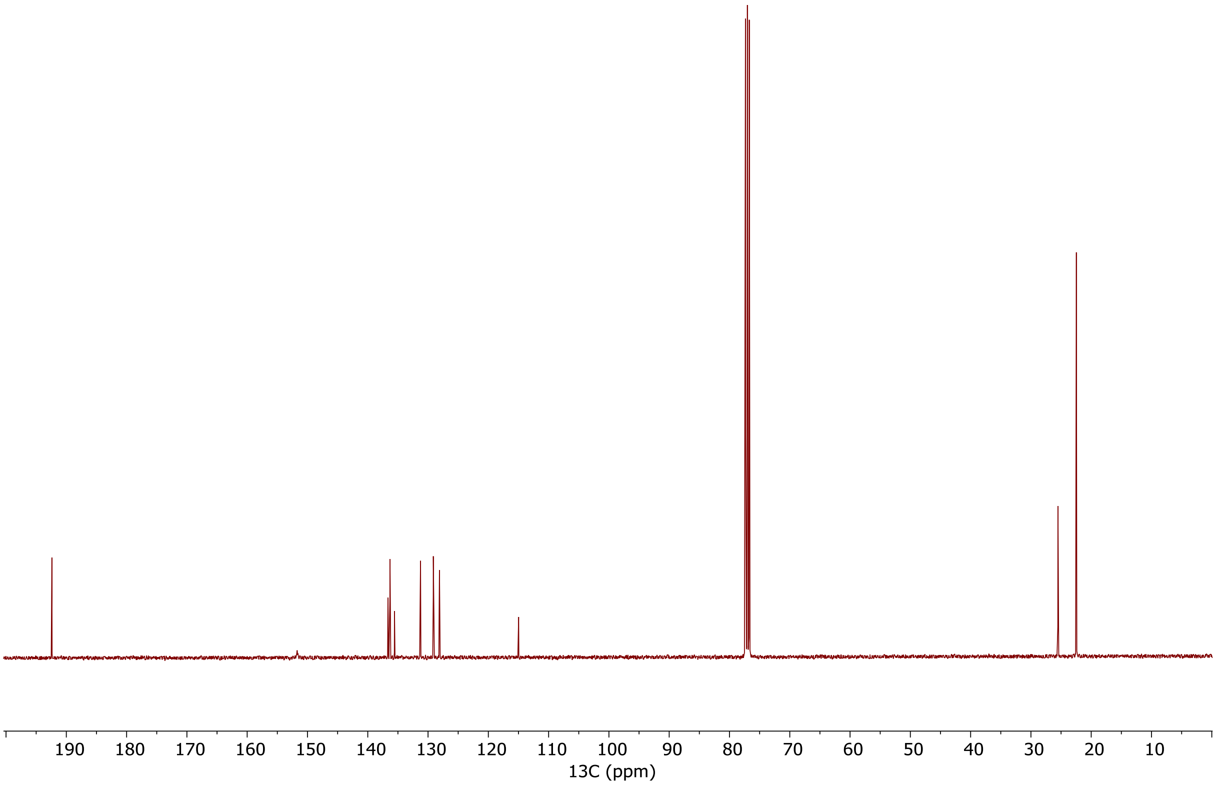


**Figure S4.** $^{13}C$ NMR spectrum (101 MHz, $CDCl_3$, 298 K) of ligand **1**.

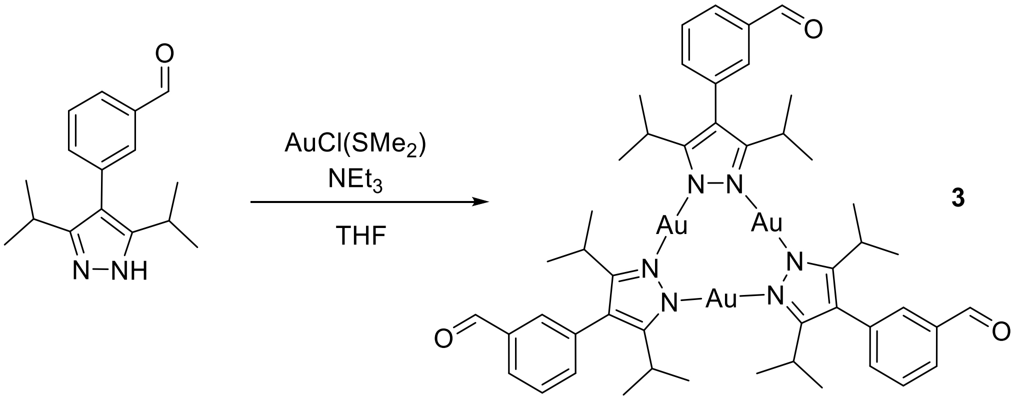


**Scheme S3.** Synthesis of complex **3.**

Ligand **1** (102 mg, 0.4 mmol, 1 eq.) and $AuCl(SMe_2)$ (120 mg, 0.4 mmol, 1 eq.) were dissolved in THF (5 mL) under an atmosphere of $N_2$. Once a clear solution was observed, $NEt_3$ (140 µL, 1 mmol, 2.5 eq.) was added. The solution turned turbid and the color changed to light purple. The mixture was stirred for 18 h at RT. Subsequently, MeOH (15 mL) was added to precipitate the product and dissolve the ammonium salt. The product was isolated by centrifugation and re-dissolved in DCM. After filtration over celite, the solvent was evaporated to give **3** as a white powder. Yield: 105 mg, 58%. $^1H$ NMR (400 MHz, $CDCl_3$, 298 K) δ 10.06 (s, 1H), 7.87 (dt, $J$ = 7.4, 1.6 Hz, 1H), 7.76 (t, $J$ = 1.7 Hz, 1H), 7.61 – 7.49 (m, 2H),3.22 (hept, $J$ = 7.2 Hz, 2H), 1.29 (d, $J$ = 7.2 Hz, 12H) $^{13}C$ NMR (101 MHz, $CDCl_3$, 298 K) δ 192.32, 155.45, 137.65, 136.58, 136.29, 132.77, 128.76, 128.31, 115.58, 28.10, 22.59. Attempts to detect the complex by HR-MS were not successful.

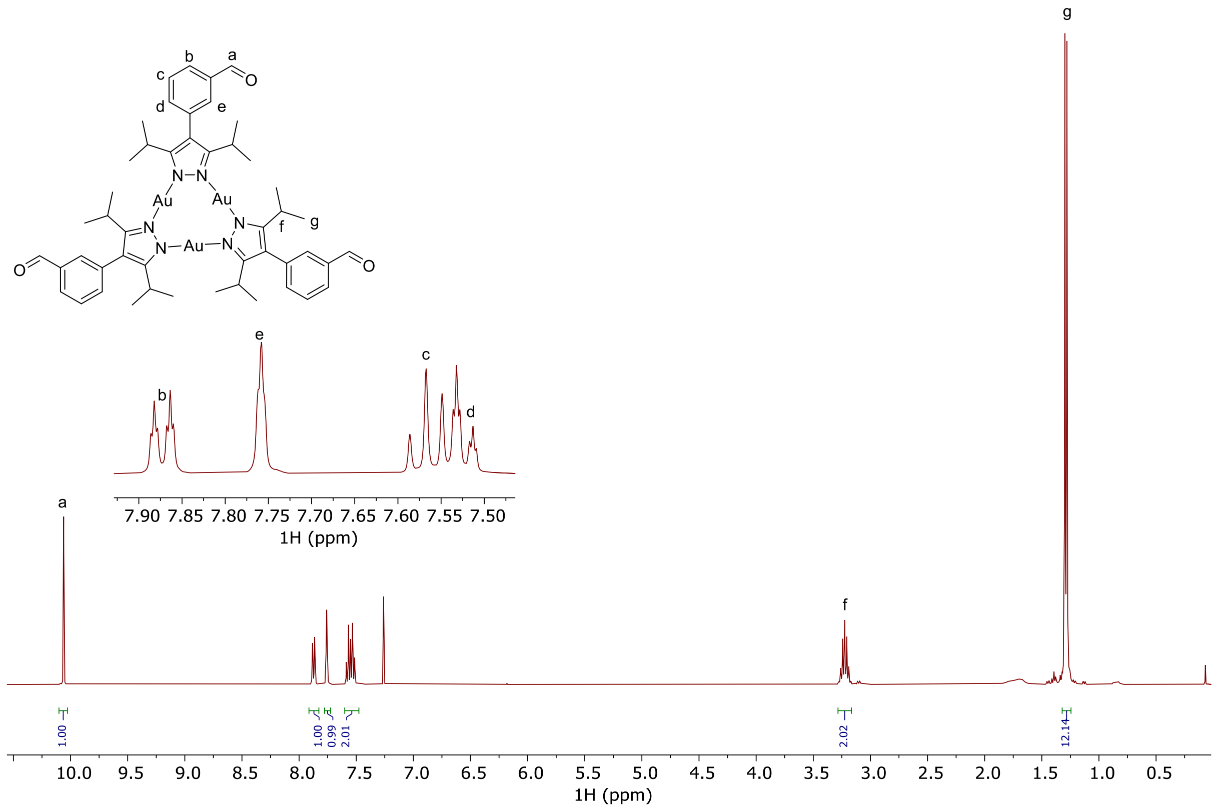


**Figure S5.** $^{1}H$ NMR spectrum (400 MHz, $CDCl_3$, 298 K) of complex **3**.

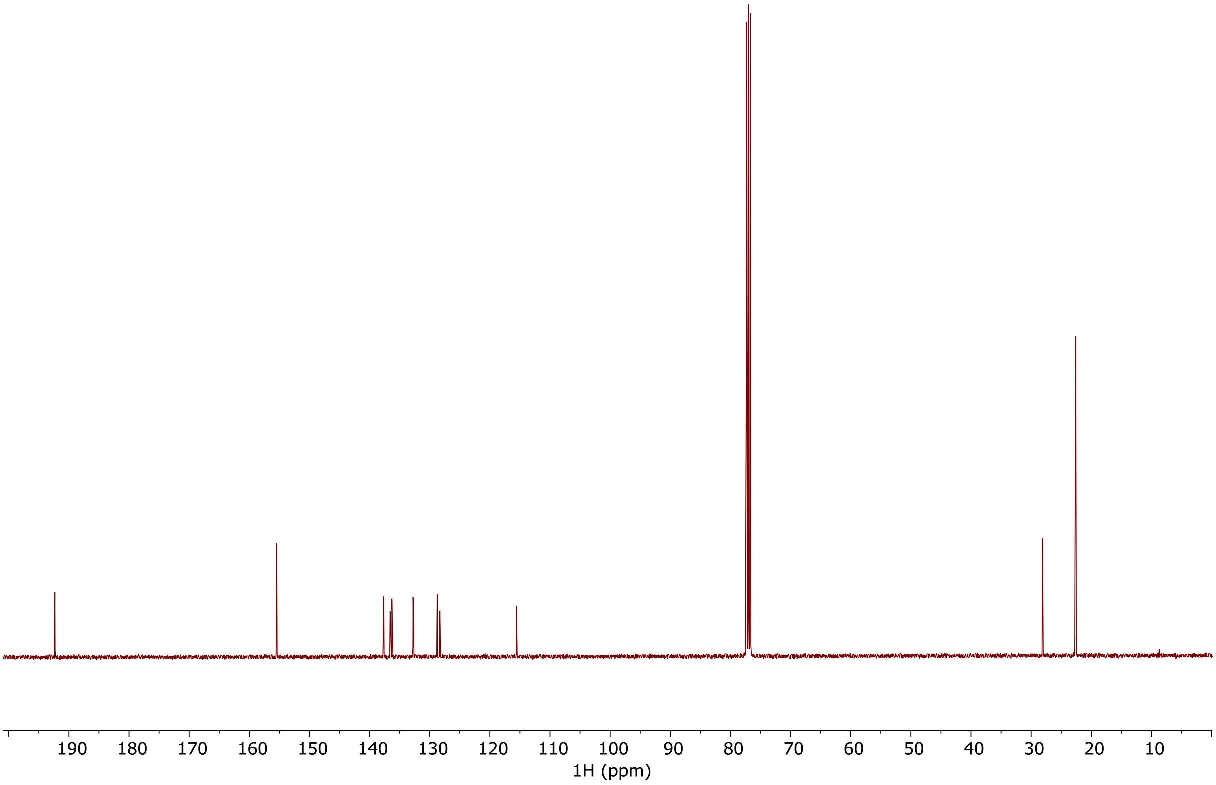


**Figure S6.** $^{13}C$ NMR spectrum (101 MHz, $CDCl_3$, 273 K) of complex **3**.

## 2.2 Synthesis of complex 4

4-(*p*-Formylphenyl)-3,5-diisopropyl-1-(*p*-toluenesulfonyl)-pyrazole (**2Ts**)

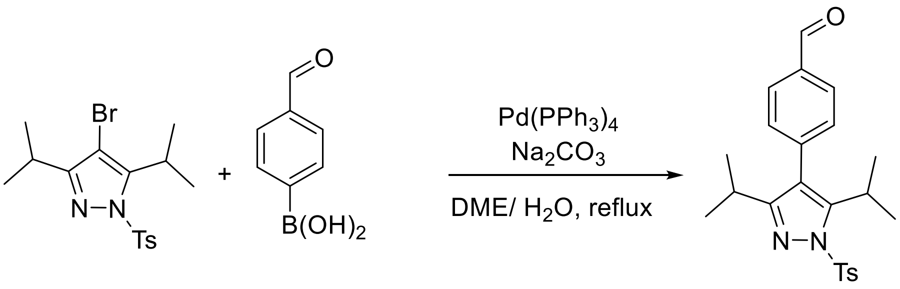


**2Ts**

**Scheme S4**. Synthesis of precursor **2Ts**.

4-Bromo-3,5-diisopropyl-1-(p-toluenesulfonyl)pyrazole (344 mg, 0.893 mmol, 1 eq.), 4-formylphenylboronic acid (268 mg, 1.79 mmol, 2 eq.), and $Pd(PPh_3)_4$ (87.7 mg, 75.9 µmol, 0.085 eq.) were dissolved in DME (15 mL) under an atmosphere of $N_2$. Saturated aqueous $Na_2CO_3$ (5 mL) was added and the mixture was heated under reflux for 18 h. After cooling to RT, the product was extracted with DCM (3 × 20 mL). The organic phase was washed with water (15 mL), saturated $NaHCO_3$ (15 mL), and brine (15 mL), dried over $MgSO_4$, and evaporated to dryness. The product was purified by column chromatography on silica gel with DCM as eluent, followed by recrystallization from cold acetonitrile. Yield: 198 mg, 54%. $^1$H NMR (400 MHz, $CDCl_3$) δ 10.05 (s, 1H), 8.12 – 7.74 (m, 4H), 7.52 – 7.28 (m, 4H), 3.81 (hept, *J* = 7.1 Hz, 1H), 2.63 (hept, *J* = 6.9 Hz, 1H), 2.44 (s, 3H), 1.44 – 0.62 (m, 12H). $^{13}$C NMR (101 MHz, $CDCl_3$) δ 191.73, 160.67, 150.19, 145.13, 140.11, 135.68, 135.62, 131.63, 129.71, 129.45, 127.95, 121.26, 26.75, 22.35, 21.72, 21.64. ESI-MS: *m*/*z* calculated for $C_{23}H_{27}N_2O_3S^+$ $[M+H]^+$ 257.1648, found 257.1648.

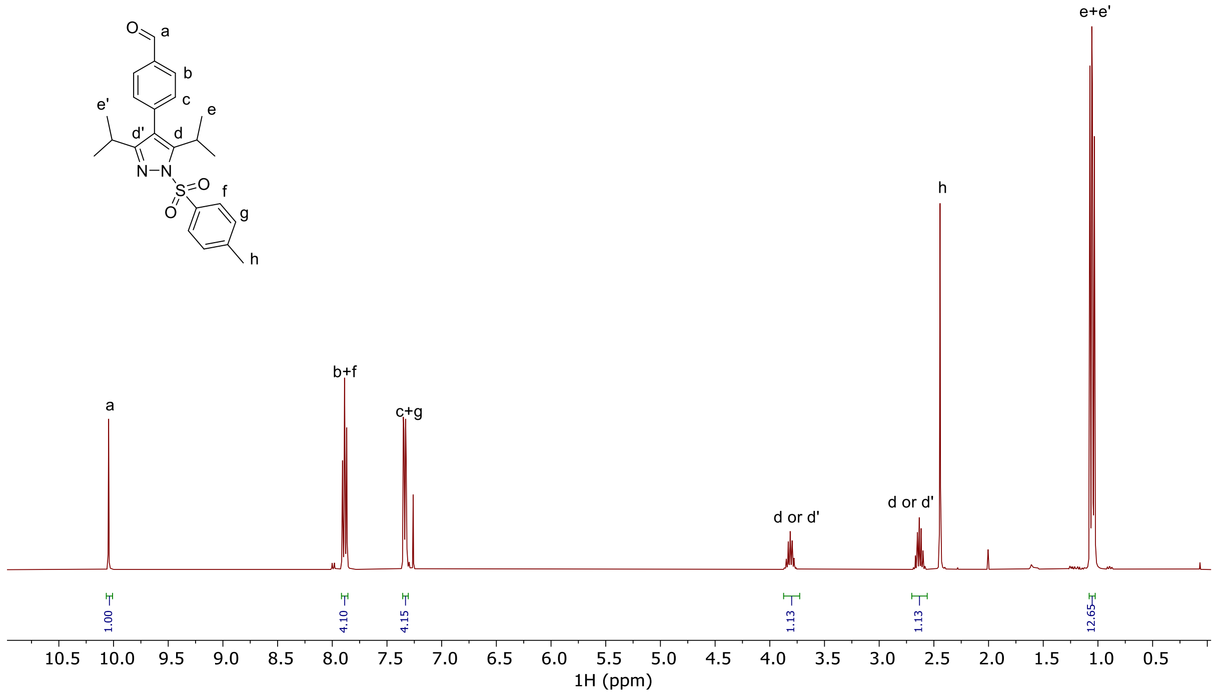


**Figure S7.** $^{1}H$ NMR spectrum (400 MHz, $CDCl_3$, 298 K) of **2Ts.**

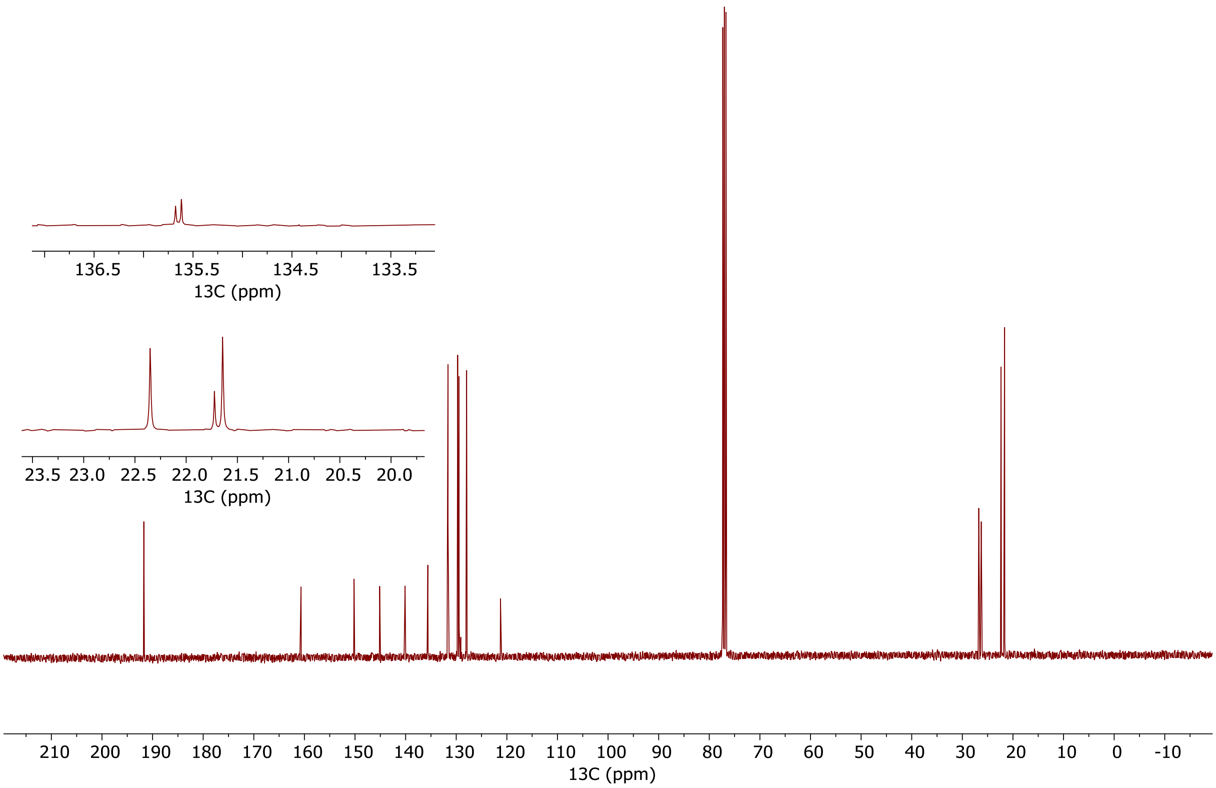


**Figure S8.** $^{13}C$ NMR spectrum (101 MHz, $CDCl_3$, 298 K) of **2Ts.**

4-(*p*-Formylphenyl)-3,5-diisopropyl-1H-pyrazole (**2**)

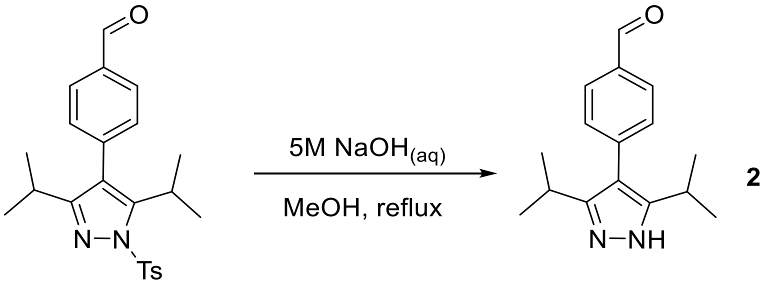


**Scheme S5.** Synthesis of ligand **2**.

5M NaOH in $H_2O$ (5 mL) was added to a solution of **2Ts** (150 mg, 0.365 mmol) in MeOH (10 mL), and. The mixture was heated under reflux for 5 h. The product was extracted with DCM (3 × 20 mL). The organic phase was washed with brine, dried over $MgSO_4$, and evaporated to dryness. The product was purified by column chromatography (1% MeOH in DCM). Yield: 88 mg, 93%. $^1H$ NMR (400 MHz, $CDCl_3$) δ 10.05 (s, 1H), 7.92 (d, $J$ = 8.3 Hz, 2H), 7.43 (d, $J$ = 8.2 Hz, 2H), 3.03 (hept, $J$ = 7.0 Hz, 2H), 1.23 (d, $J$ = 7.0 Hz, 12H). $^{13}C$ NMR (101 MHz, $CDCl_3$) δ 191.94, 141.37, 134.69, 130.61, 129.88, 115.27, 25.52, 22.48, *C*N was not detected. ESI-MS: *m*/*z* calculated for $C_{16}H_{21}N_2O^+$ $[M+H]^+$ 411.1737, found 411.1745.

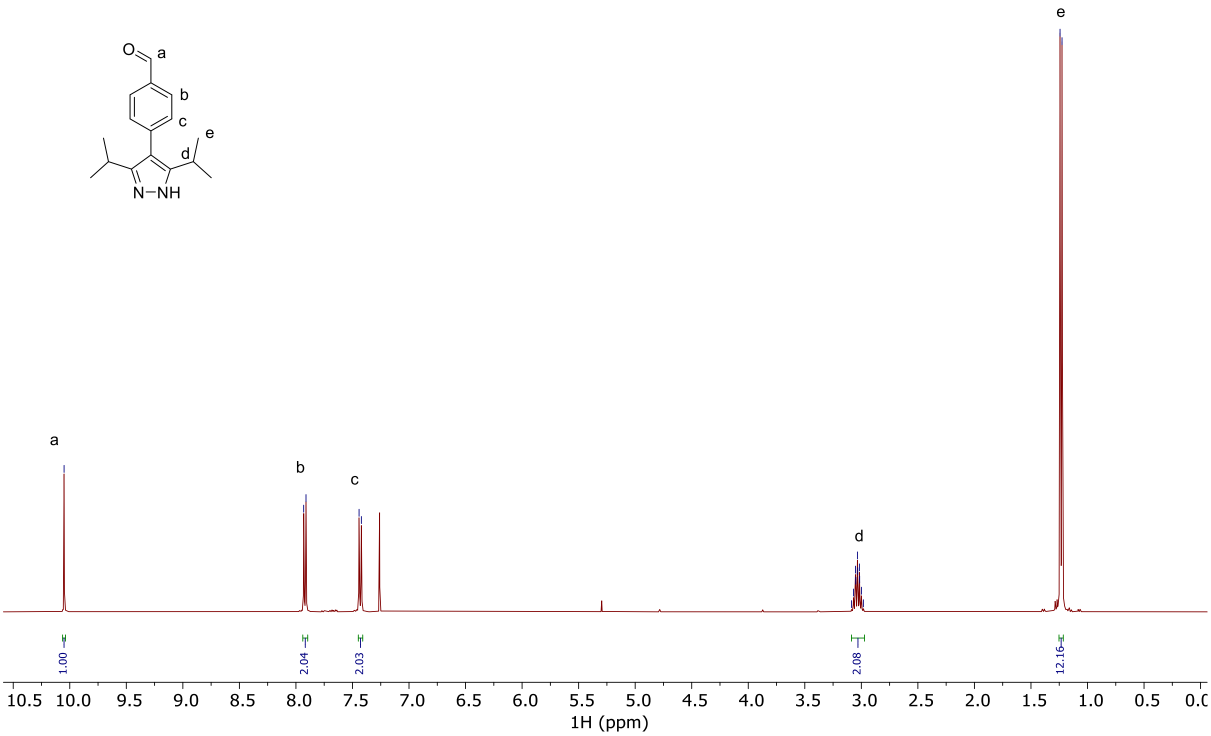


**Figure S9.** $^1H$ NMR spectrum (400 MHz, $CDCl_3$, 298 K) of ligand **2.**

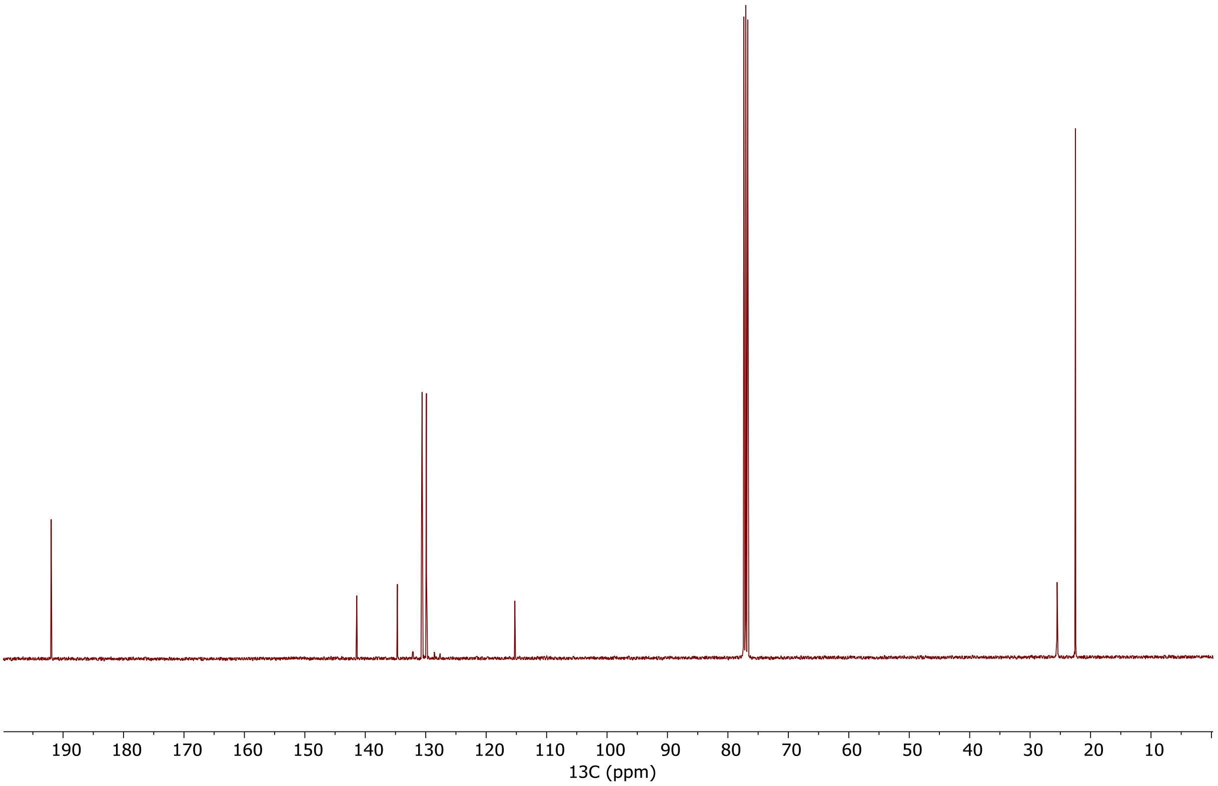


**Figure S10.** $^{13}C$ NMR spectrum (101 MHz, $CDCl_3$, 298 K) of ligand **2**.

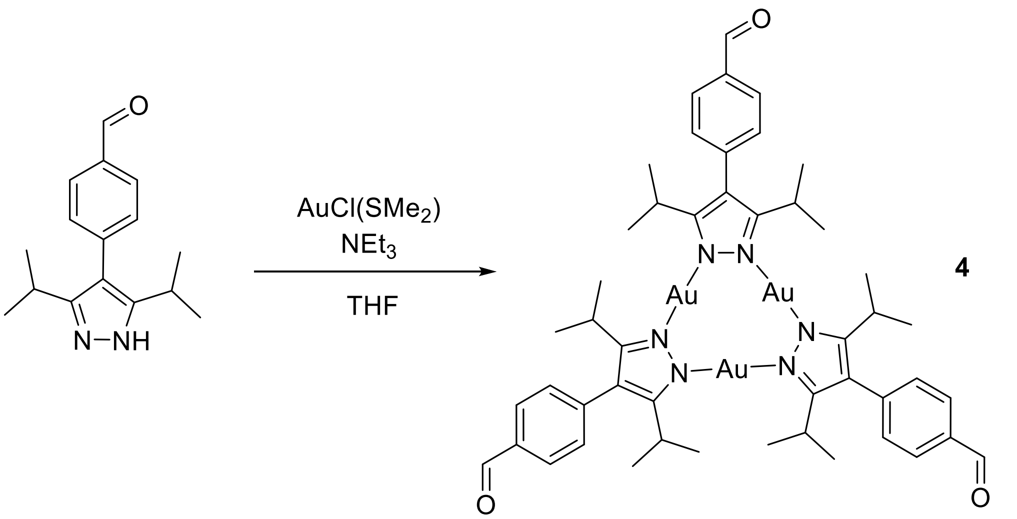


**Scheme S6.** Synthesis of complex **4**.

Ligand **2** (60 mg, 348 µmol, 1 eq.) and AuCl($SMe_2$) (103 mg, 348 µmol, 1 eq.) were dissolved in THF (8 mL) under an atmosphere of $N_2$. Once a clear solution was observed, $NEt_3$ (96.9 µL, 697 µmol, 2 eq.) was added. The solution turned turbid and the color changed to light purple. The mixture was stirred for 18 h at RT. MeOH (15 mL) was added to precipitate the product and dissolve the ammonium salt. The product was isolated by centrifugation and re-dissolved in DCM (5 × 25 mL). The solvent was evaporated to give **4** as a white powder. Yield: 95 mg, 60%. $^{1}H$ NMR (400 MHz, $CDCl_3$, 298 K) δ 10.07 (s, 1H), 7.91 (d, $J$ = 8.2 Hz, 2H), 7.43 (d, J = 8.1 Hz, 2H), 3.24 (hept, J = 7.1 Hz, 2H), 1.30 (d, J = 7.1 Hz, 12H). $^{13}C$ NMR (101 MHz, $CDCl_3$, 298K) δ 191.94,

155.27, 142.43, 135.14, 132.23, 129.39, 116.03, 28.08, 22.54. Attempts to detect the compound by HR-MS did not succeed.

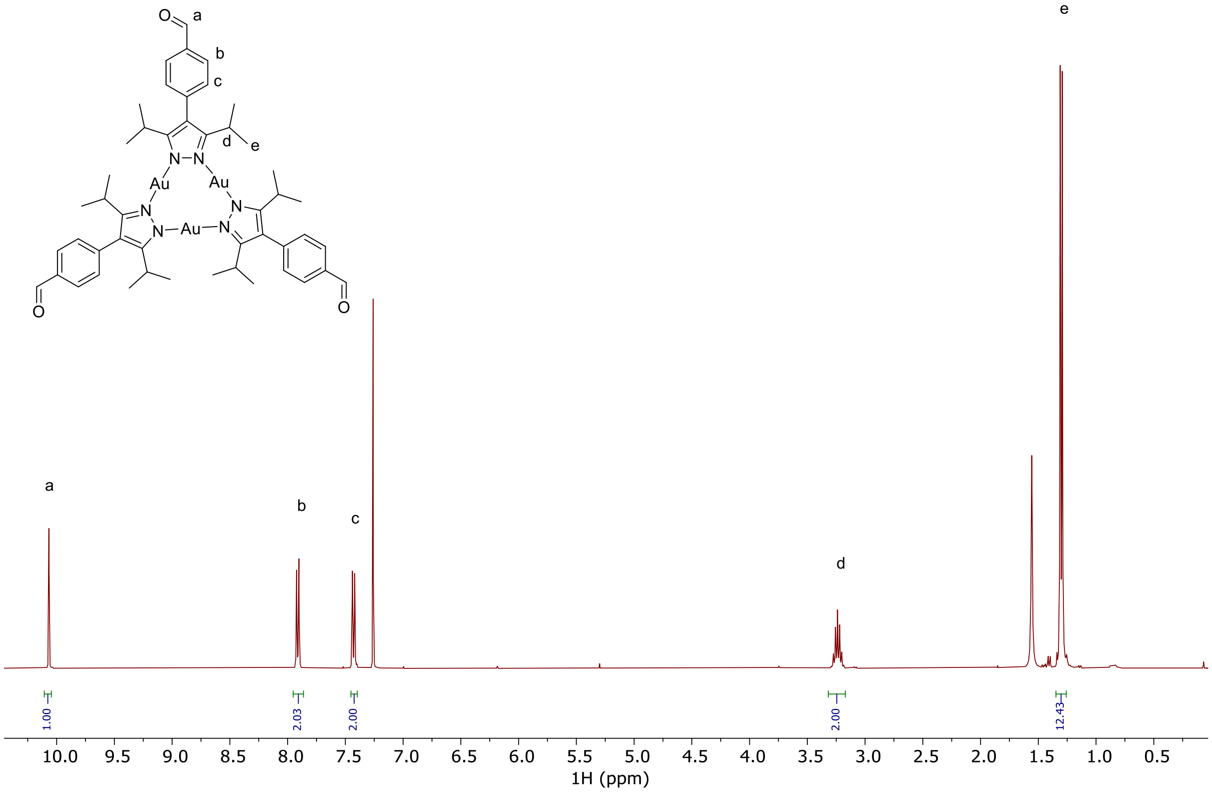


**Figure S11.** $^{1}H$ NMR spectrum (400 MHz, $CDCl_3$, 298 K) of complex **4**.

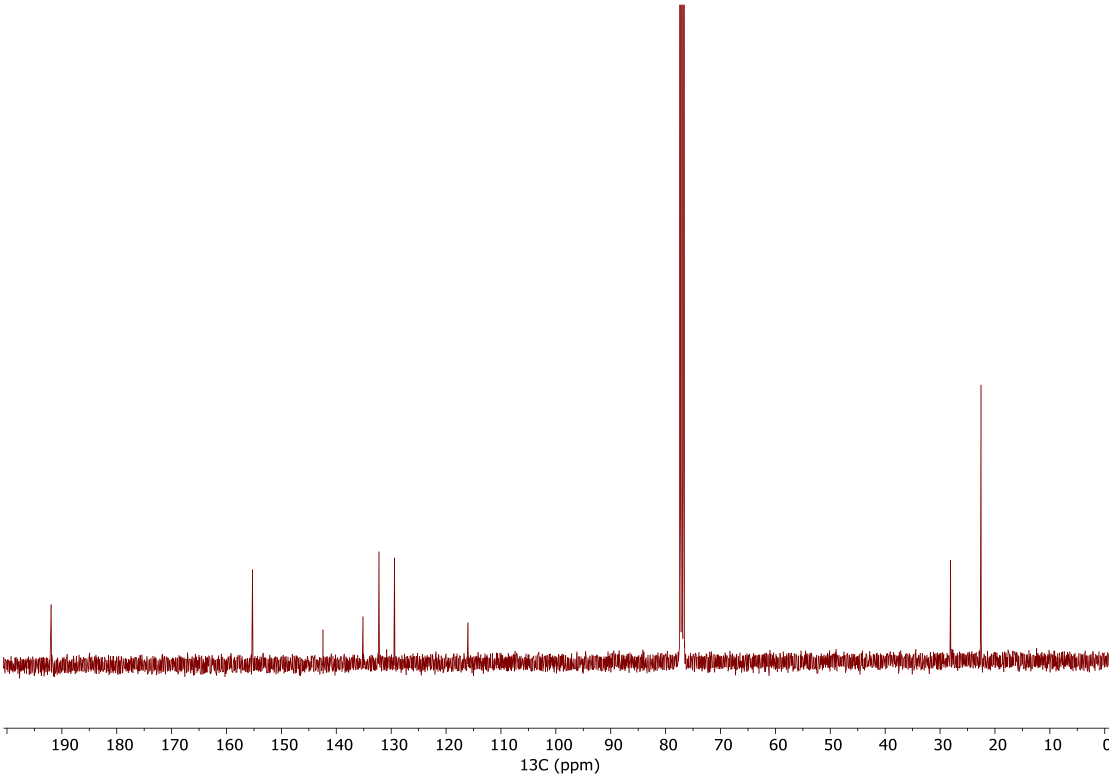


**Figure S12.** $^{13}C$ NMR spectrum (101 MHz, $CDCl_3$, 298 K) of complex **4**.

## 2.3 Synthesis of cage 5

A solution of 1,3-diaminopropane (22.5 µmol, 1.9 µL, 3 eq.) in a mixture of DCM (1.05 mL) and MeOH (1 mL) was added dropwise over 30 min to a stirred solution of complex **3** (15 µmol, 20.3 mg, 2 eq.) in a mixture of DCM (2.1 mL) and MeOH (1 mL). The solution was kept without stirring for 3 days. Subsequently, the solution was concentrated under reduced pressure. The resulting precipitate was isolated by centrifugation and dried under vacuum. Yield: 19.2 mg, 90 %. $^1$H NMR (800 MHz, $CDCl_3$) δ 8.30 (s, 1H), 7.80 (td, *J* = 7.8, 1.4 Hz, 1H), 7.57 (t, *J* = 1.8 Hz, 1H), 7.42 (t, *J* = 7.7 Hz, 1H), 7.30 (dt, *J* = 7.5, 1.5 Hz, 1H), 3.69 (t, *J* = 6.0 Hz, 2H), 3.24 (hept, *J* = 7.2 Hz, 2H), 2.18 (h, *J* = 5.8 Hz, 1H), 1.29 (dd, *J* = 7.2, 1.6 Hz, 12H).$^{13}$C NMR (201 MHz, $CDCl_3$) δ 161.56, 155.34, 136.22, 136.06, 133.92, 132.20, 128.38, 125.95, 116.51, 57.97, 31.65, 28.19, 22.77, 22.68.

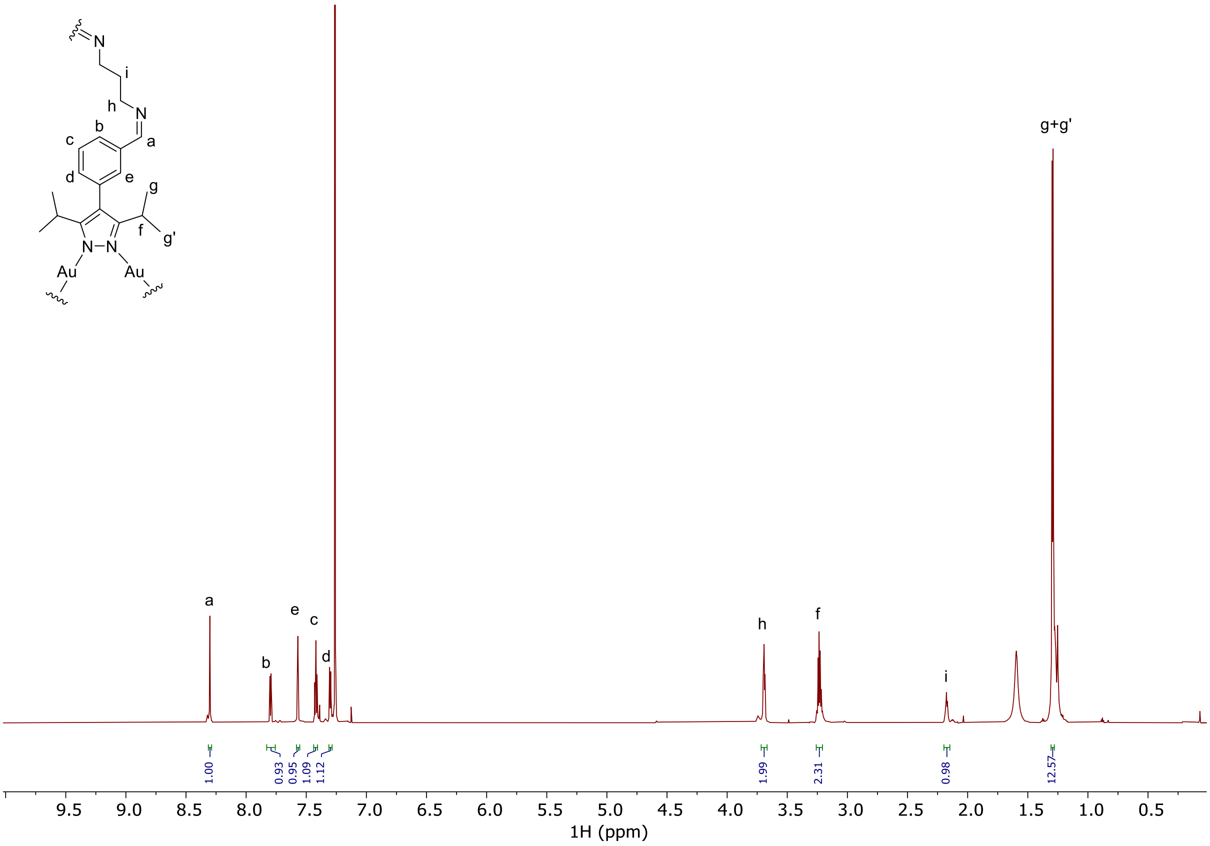


**Figure S13.** $^1$H NMR spectrum (800 MHz, $CDCl_3$, 298 K) of cage **5**.

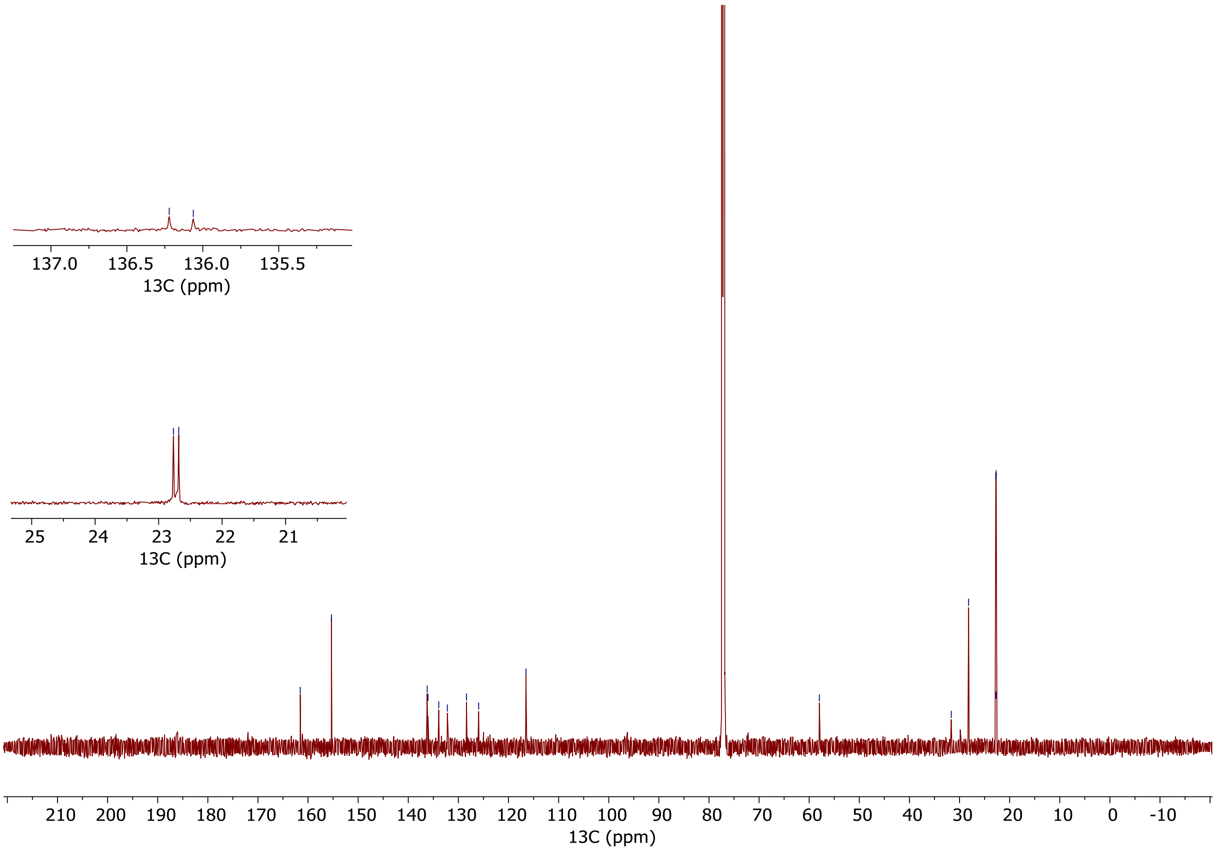


**Figure S14.** $^{13}$C NMR spectrum (201 MHz, $CDCl_3$, 298 K) of cage **5**.

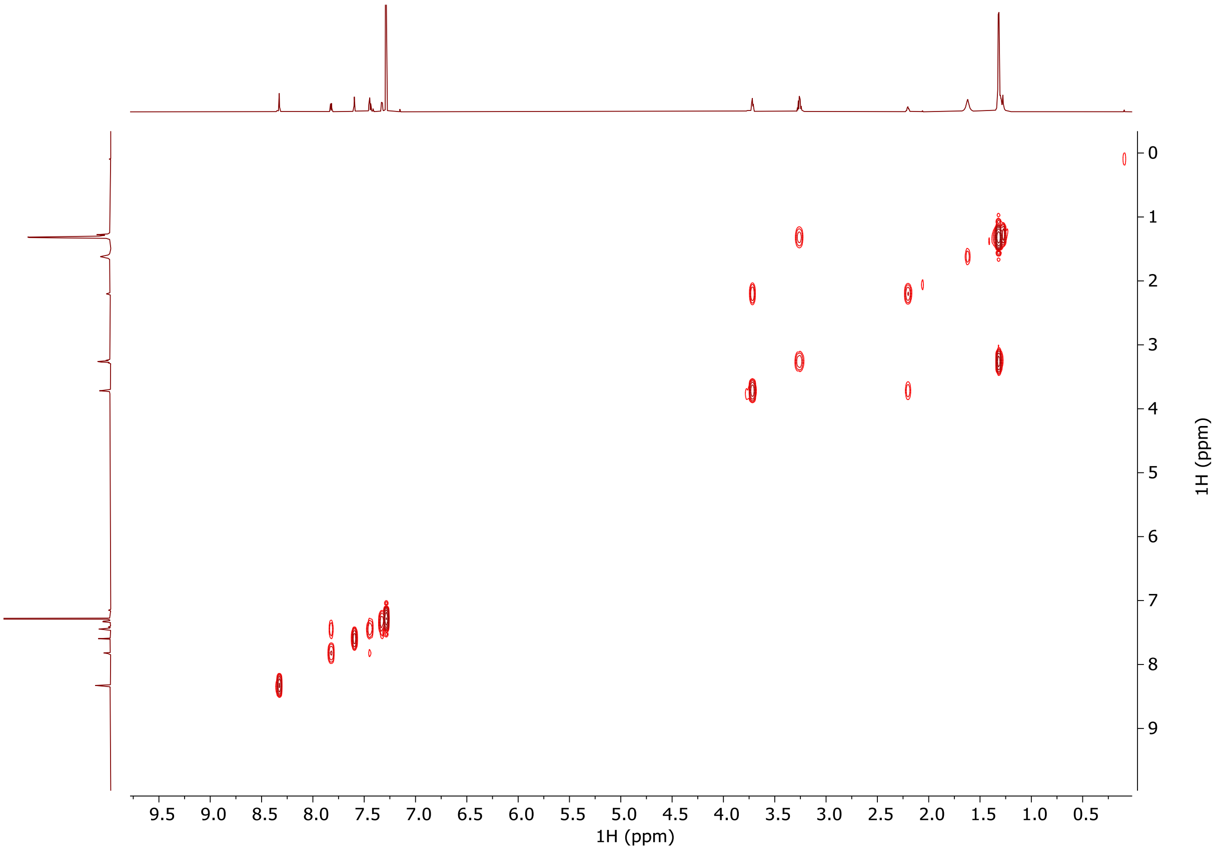


**Figure S15.** $^{1}$H-$^{1}$H COSY NMR spectrum (800 MHz, $CDCl_3$, 298 K) of cage **5**.

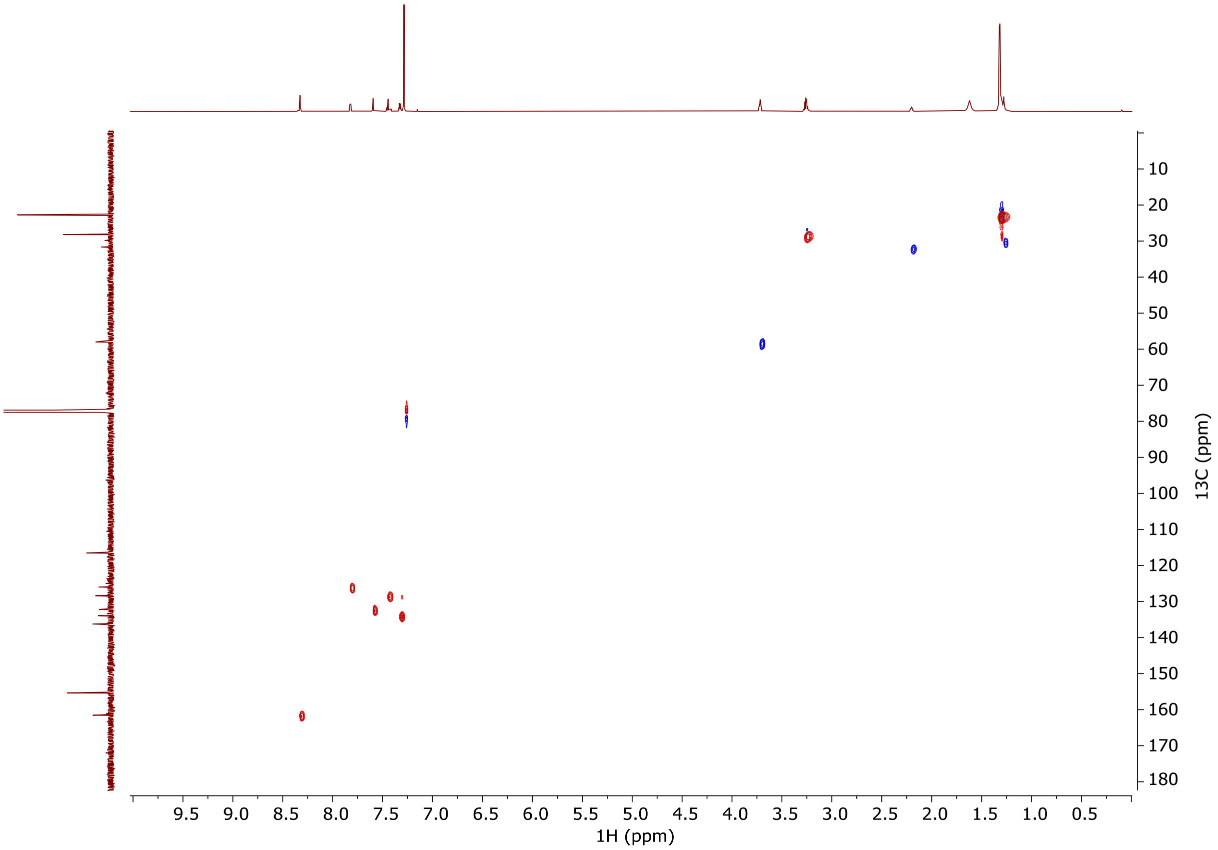


**Figure S16.** $^{1}H$-$^{13}C$ HSQC NMR spectrum (800 MHz, $CDCl_3$, 298 K) of cage **5**.

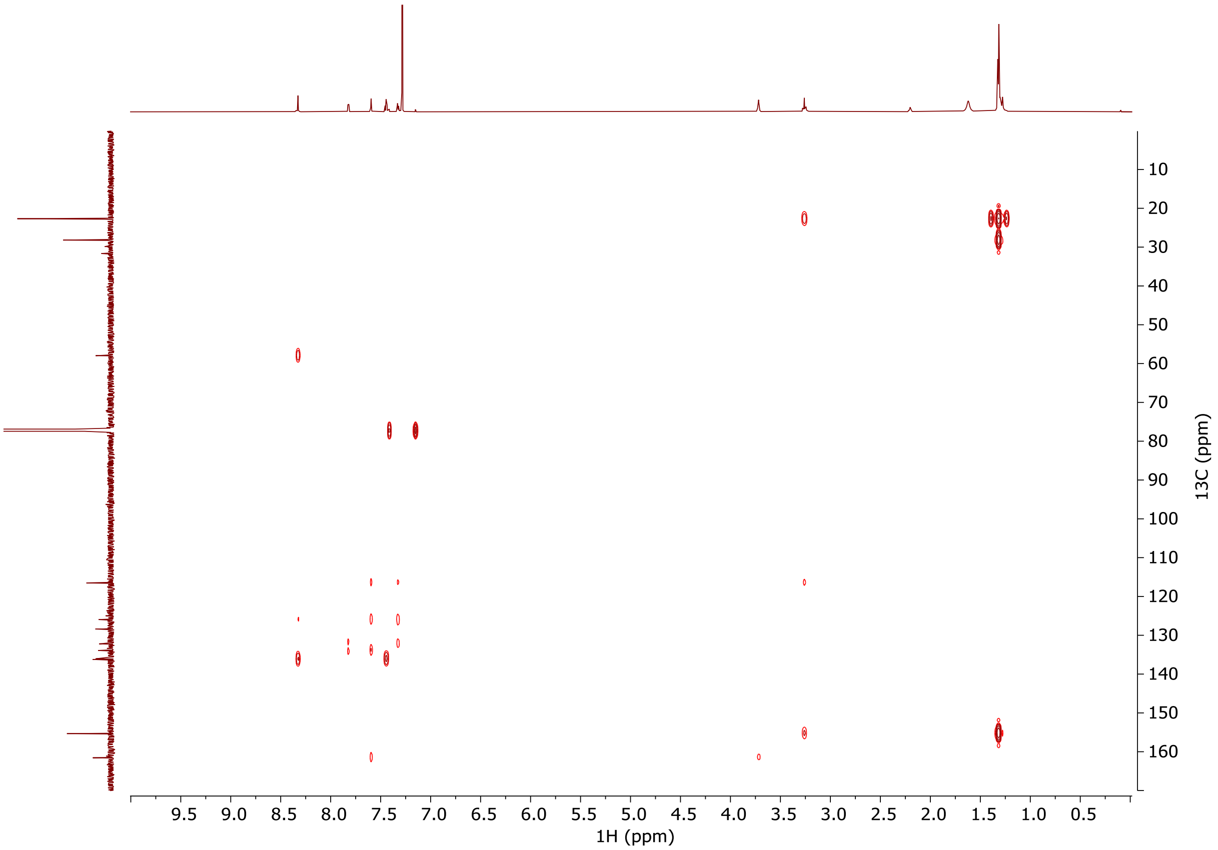


**Figure S17.** $^{1}H$-$^{13}C$ HMBC NMR spectrum (800 MHz, $CDCl_3$, 298 K) of cage **5**.

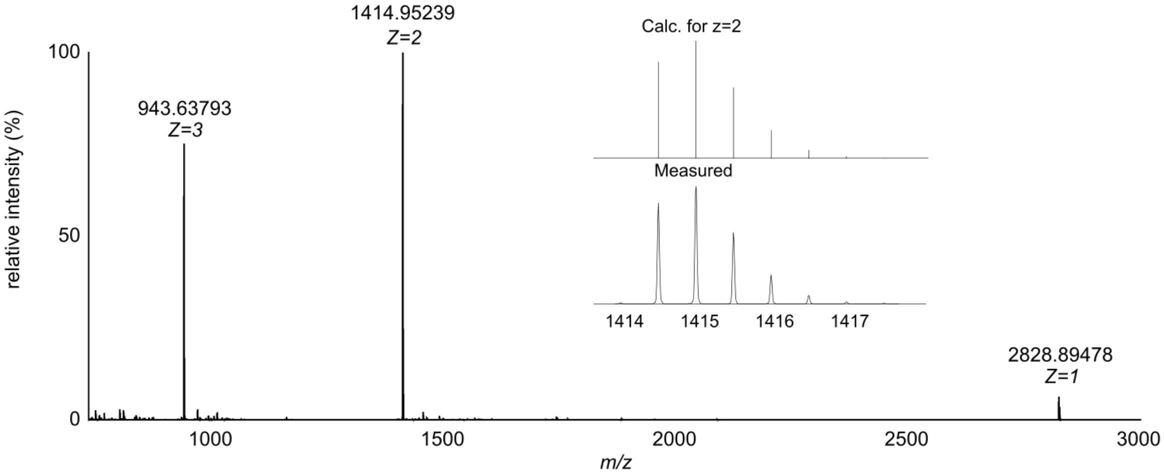


**Figure S18.** ESI-HRMS (positive mode) of cage **5** in $CHCl_3/CH_3CN$ 10:90 %v/v. Insert: calculated and measured ESI-HRMS spectrum for $C_{105}H_{134}Au_6N_{18}$ [**5**+2H]$^{2+}$.

## 2.4 Synthesis of cage 6

A solution of complex **4** (50 mg, 36.8 µmol, 2 eq.) in $C_2H_2Cl_4$ (10 mL) was added dropwise over 2 h to a stirred solution of *m*-xylylenediamine (7.43 µL, 56.2 µmol, 3.05 eq.) in $C_2H_2Cl_4$ (10 mL). The solution was stirred for 3 days. Subsequently, acetonitrile (80 mL) was added, resulting in the precipitation of the product. The product was isolated by centrifugation, washed with acetonitrile (2 × 15 mL) and pentane (15 mL), and dried under vacuum. Yield: 51 mg, 91%. $^{1}$H NMR (800 MHz, $C_2D_2Cl_4$) δ 8.46 (s, 2H), 7.78 (s, 4H), 7.54 (s, 1H), 7.34 (t, *J* = 7.6 Hz, 1H), 7.21 (m, 7H), 4.90 (s, 4H), 3.23 (h, *J* = 7.2 Hz, 4H), 1.24 (d, *J* = 7.2 Hz, 12H), 1.17 (d, *J* = 7.2 Hz, 12H). $^{13}$C NMR (201 MHz, $C_2D_2Cl_4$) δ 162.46, 155.29, 140.22, 138.58, 135.00, 132.13, 128.22, 126.02, 125.86, 123.63, 116.15, 64.11, 28.43, 22.40, 22.38.

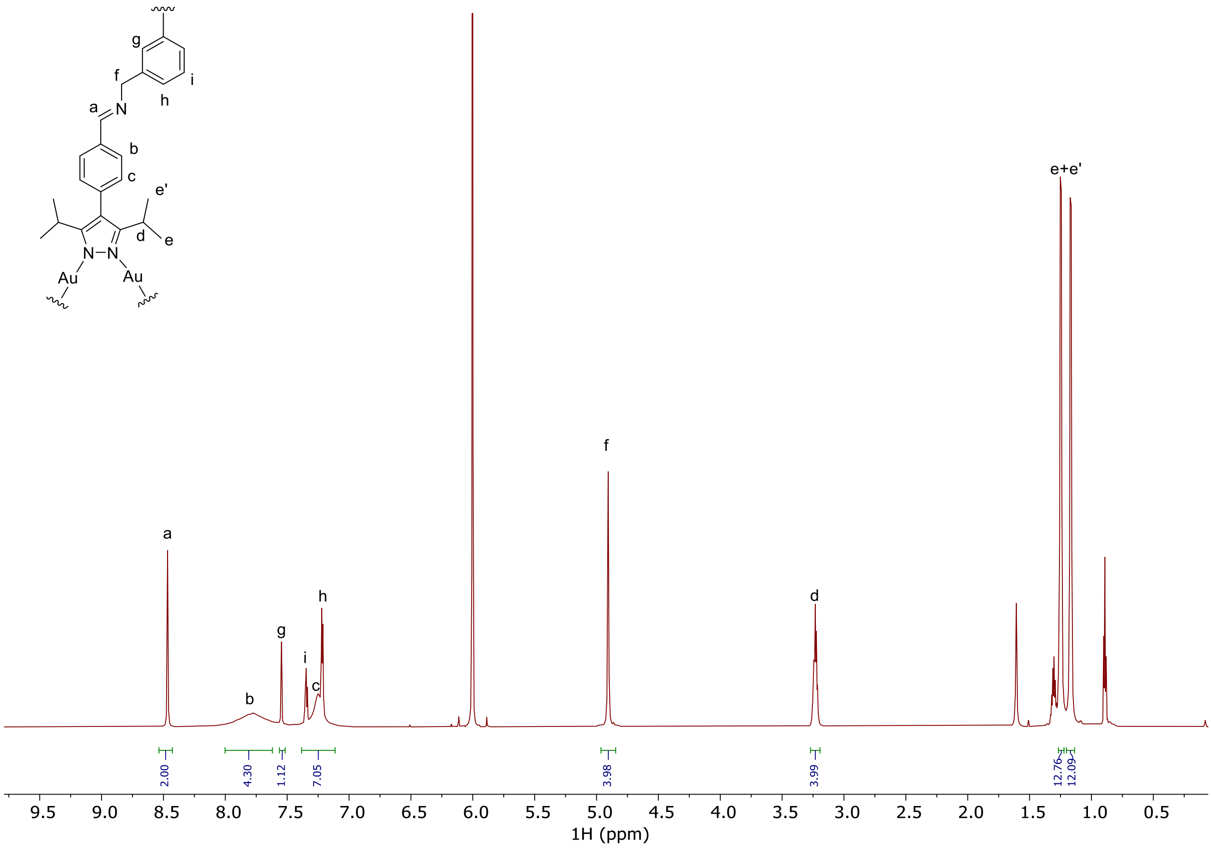


**Figure S19.** $^{1}H$ NMR spectrum (800 MHz, $C_2D_2Cl_4$, 298 K) of cage **6**.

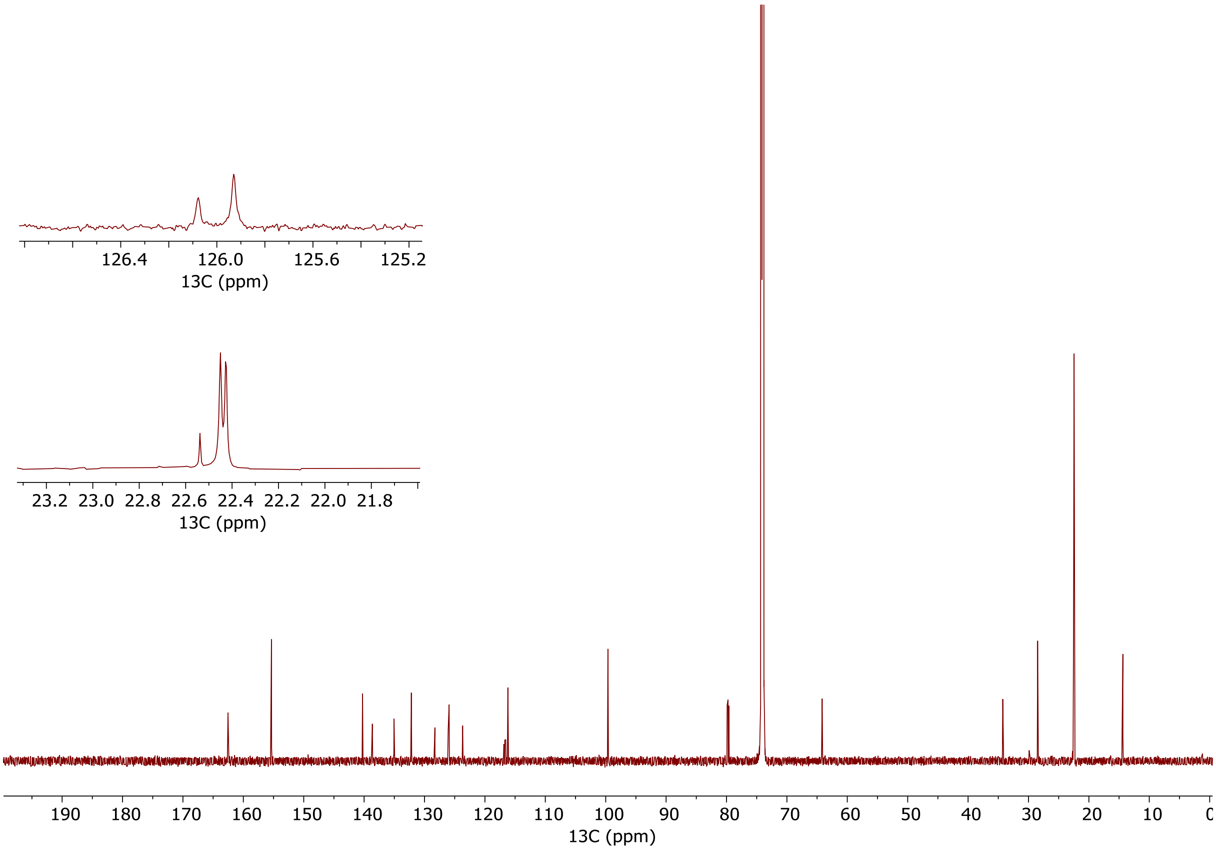


**Figure S20.** $^{13}C$ NMR spectrum (201 MHz, $C_2D_2Cl_4$, 298 K) of cage **6**.

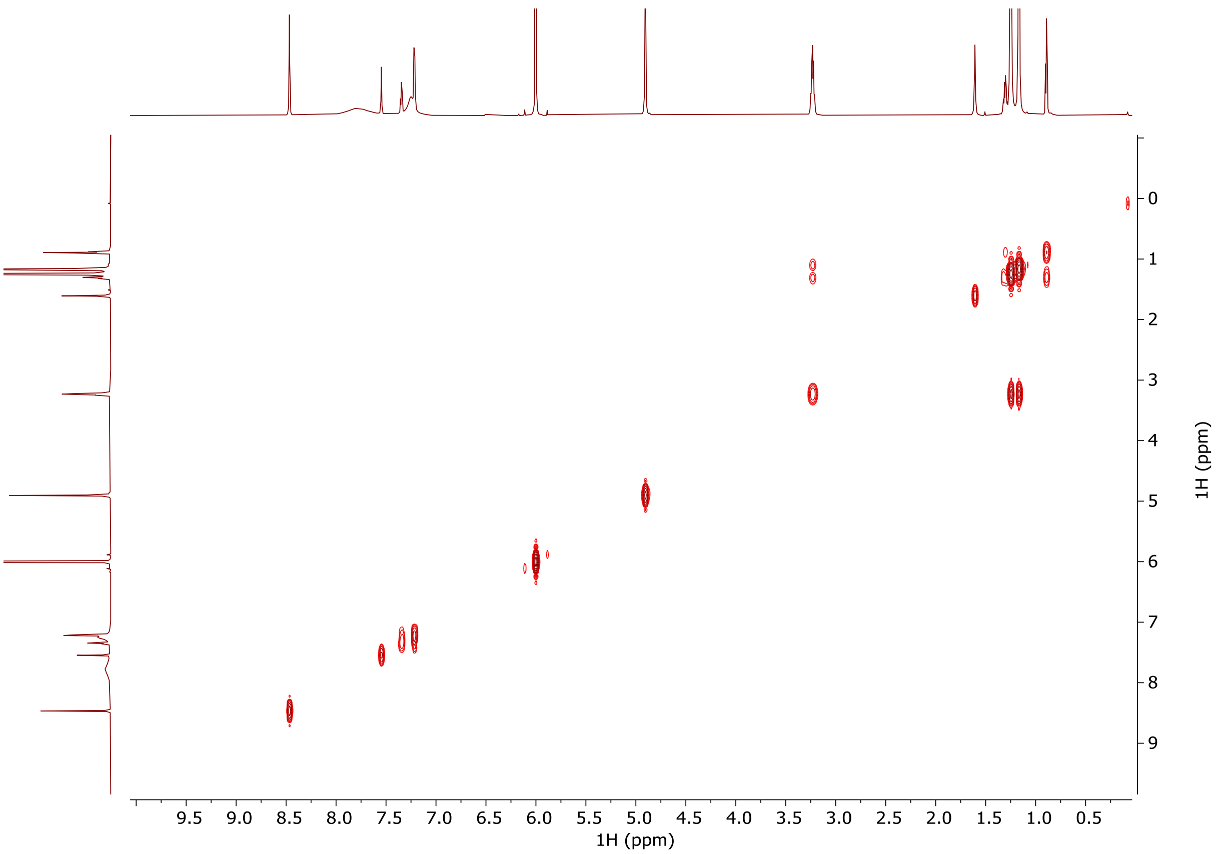


**Figure S21.** $^1$H-$^1$H COSY NMR spectrum (800 MHz, $C_2D_2Cl_4$, 298 K) of cage **6.**

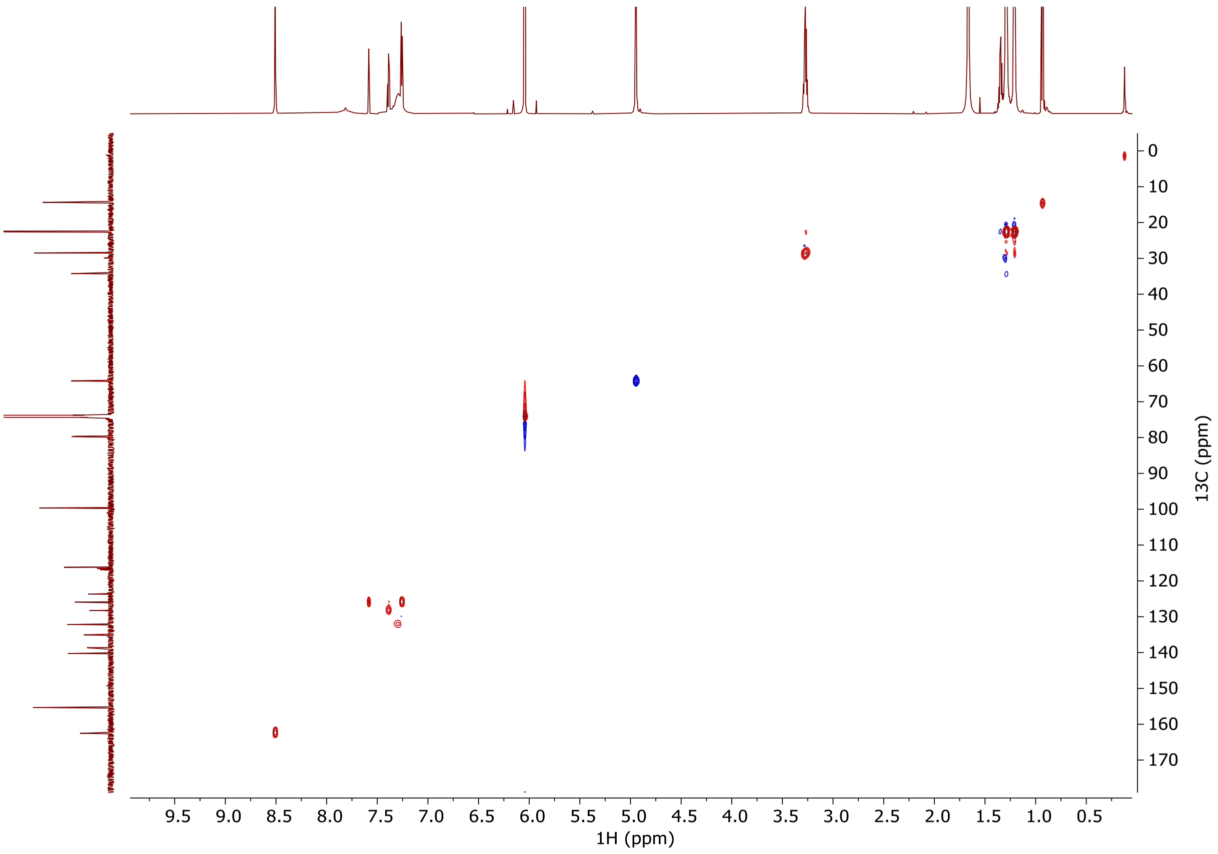


**Figure S22.** $^1$H-$^{13}$C HSQC NMR spectrum (800 MHz, $C_2D_2Cl_4$, 298 K) of cage **6.**

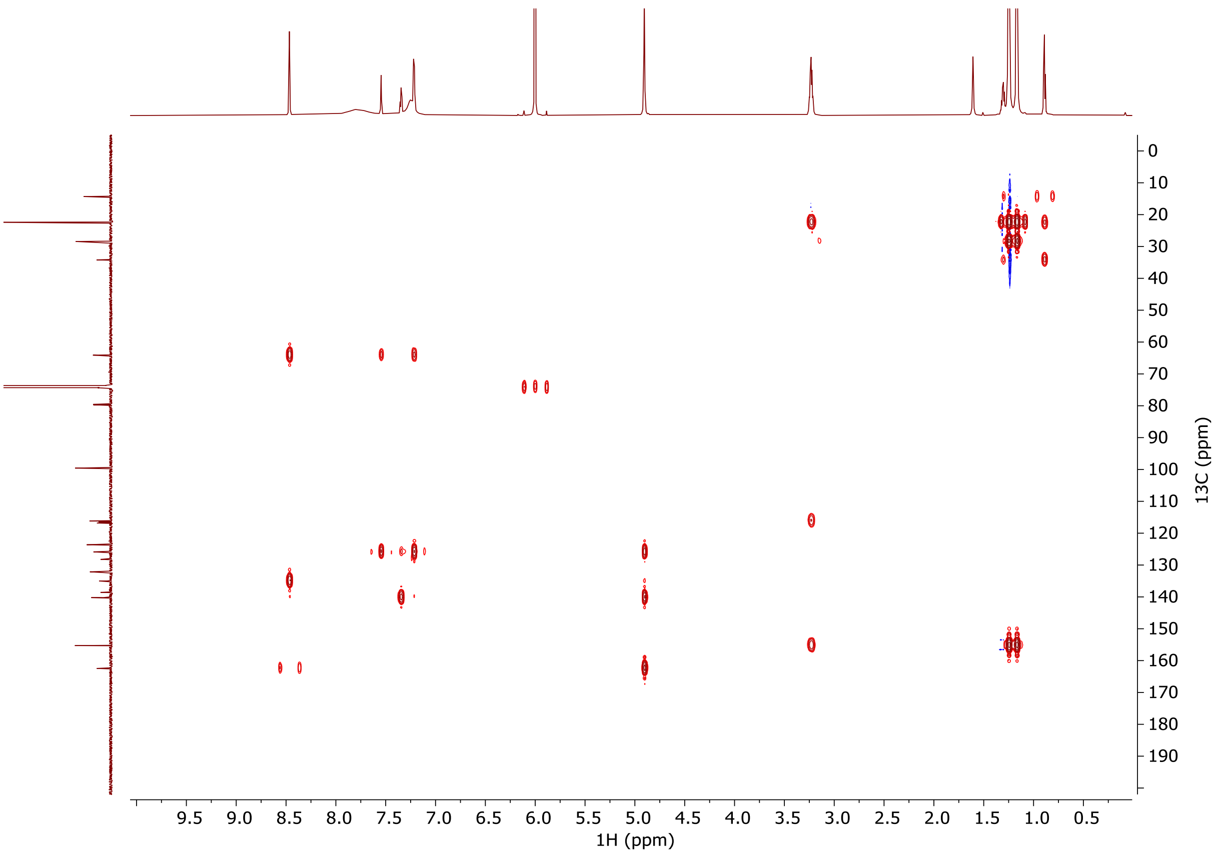


**Figure S23.** $^1H$-$^{13}C$ HMBC NMR spectrum (800 MHz, $C_2D_2Cl_4$, 298 K) of cage **6.**

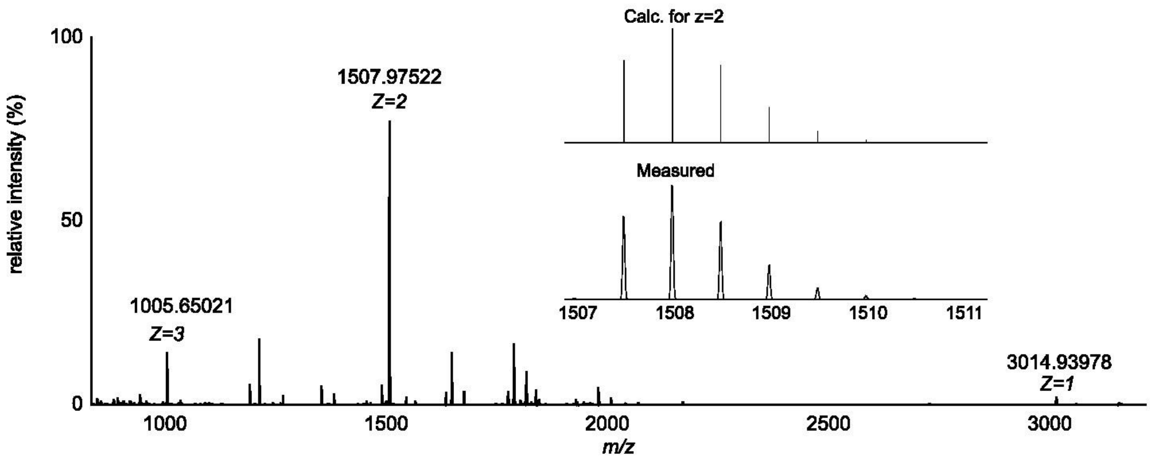


**Figure S24.** ESI-HRMS (positive mode) of cage **6** in $C_2H_2Cl_4$/$CH_3CN$ 10:90 %v/v. Insert: calculated and measured ESI-HRMS spectrum for $C_{120}H_{140}Au_6N_{18}$ [**6**+2H]$^{2+}$.

## 2.5 Synthesis of cage 7

A solution of TREN (5.88 µL, 39.3 µmol, 1.05 eq.) in $CHCl_3$ (5 mL) was added dropwise over 2 h to a stirred solution of complex **4** (50.8 mg, 37.4 µmol, 1 eq.) in $CHCl_3$ (8 mL). The solution was kept without stirring for 3 days. The mixture was concentrated under reduced pressure to a volume of approximately 2 mL, acetonitrile was added, and the product precipitated. The product was isolated by centrifugation and dried under vacuum. Yield: 49.2 mg, 91%. $^{1}$H NMR spectrum (400 MHz, $CDCl_3$, *273 K*) δ 8.12 (d, *J* = 7.8 Hz, 1H), 7.70 (s, 1H), 7.37 (d, *J* = 7.7 Hz, 1H), 7.03 (d, *J* = 7.2 Hz, 1H), 6.43 (s, 1H), 3.85 (d, *J* = 10.3 Hz, 1H), 3.36 (p, *J* = 7.1 Hz, 1H), 3.24 (t, 1H), 3.00 – 2.84 (m, 2H), 2.79 – 2.72 (m, 1H), 1.32 – 1.11 (m, 12H). $^{13}$C NMR (101 MHz, $CDCl_3$) δ 161.47, 155.79, 154.47, 138.17, 134.95, 132.16, 131.76, 130.65, 124.90, 116.20, 60.25, 56.83, 28.77, 27.16, 23.72, 22.90, 22.45.

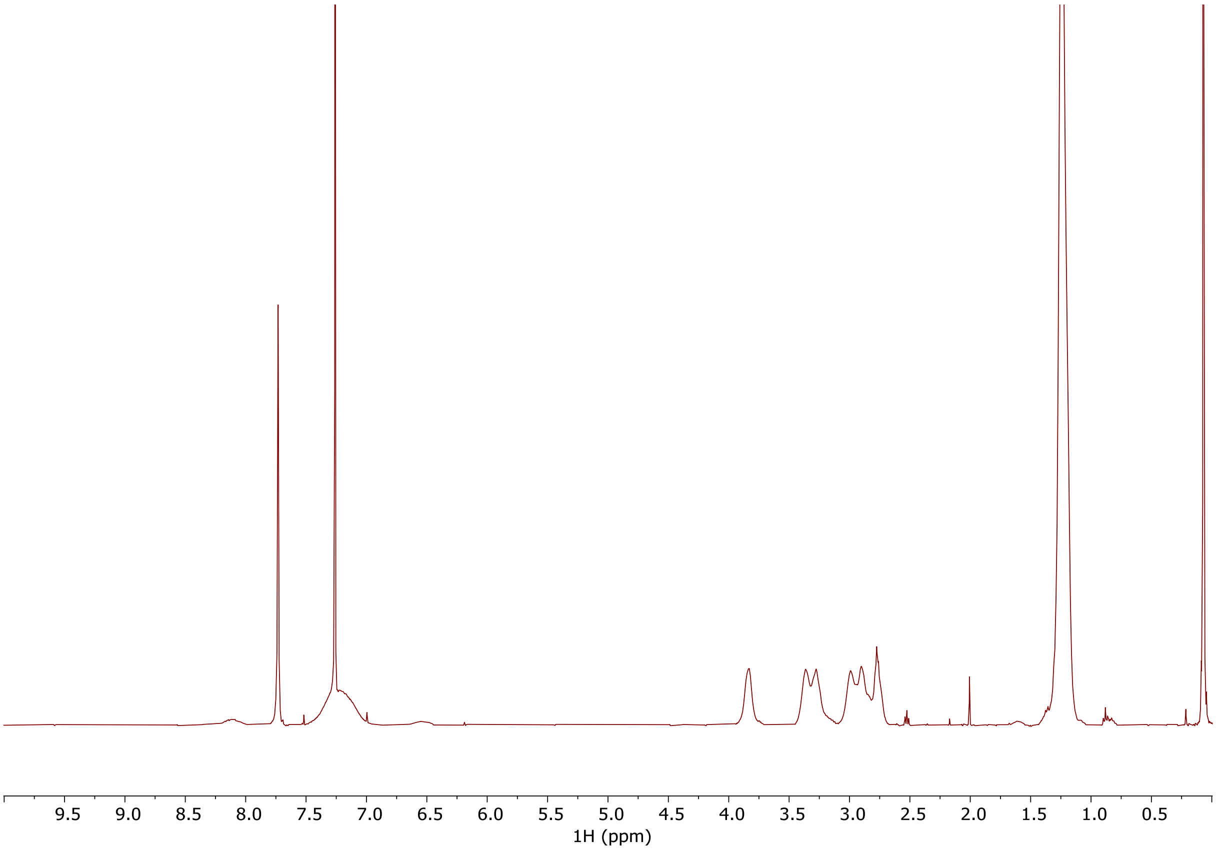


**Figure S25.** $^{1}$H NMR spectrum (400 MHz, $CDCl_3$, 298 K) of cage **7**.

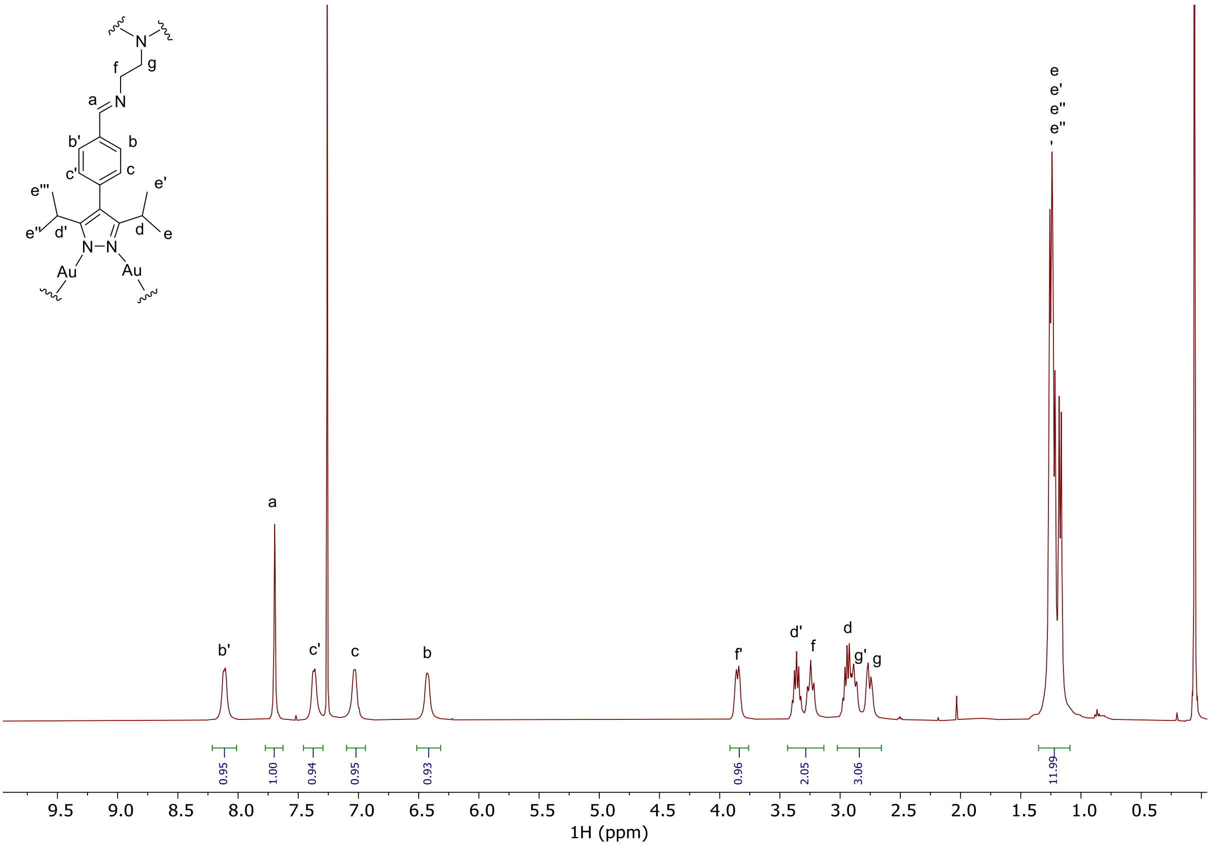


**Figure S26.** $^{1}H$ NMR spectrum (400 MHz, $CDCl_3$, 273 K) of cage **7**.

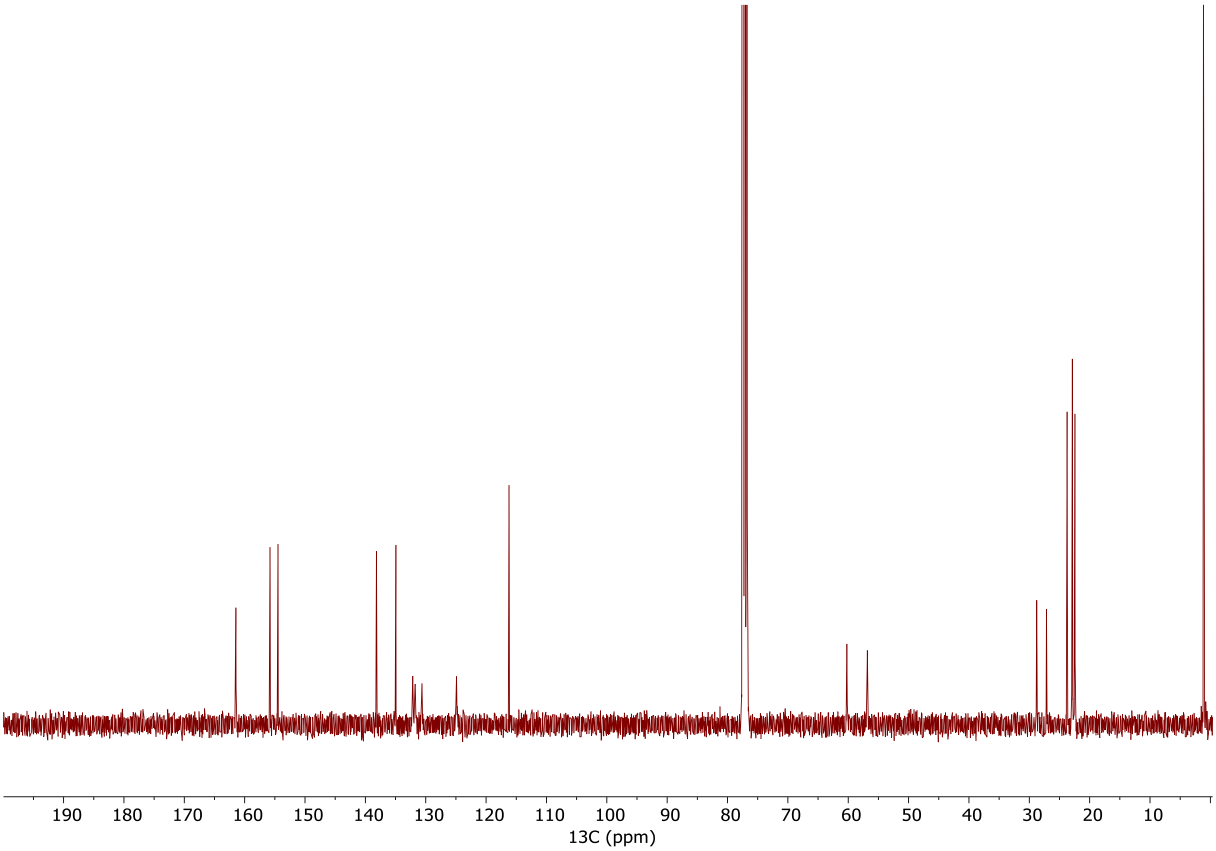


**Figure S27.** $^{13}C$ NMR spectrum (101 MHz, $CDCl_3$, 273 K) of cage **7**.

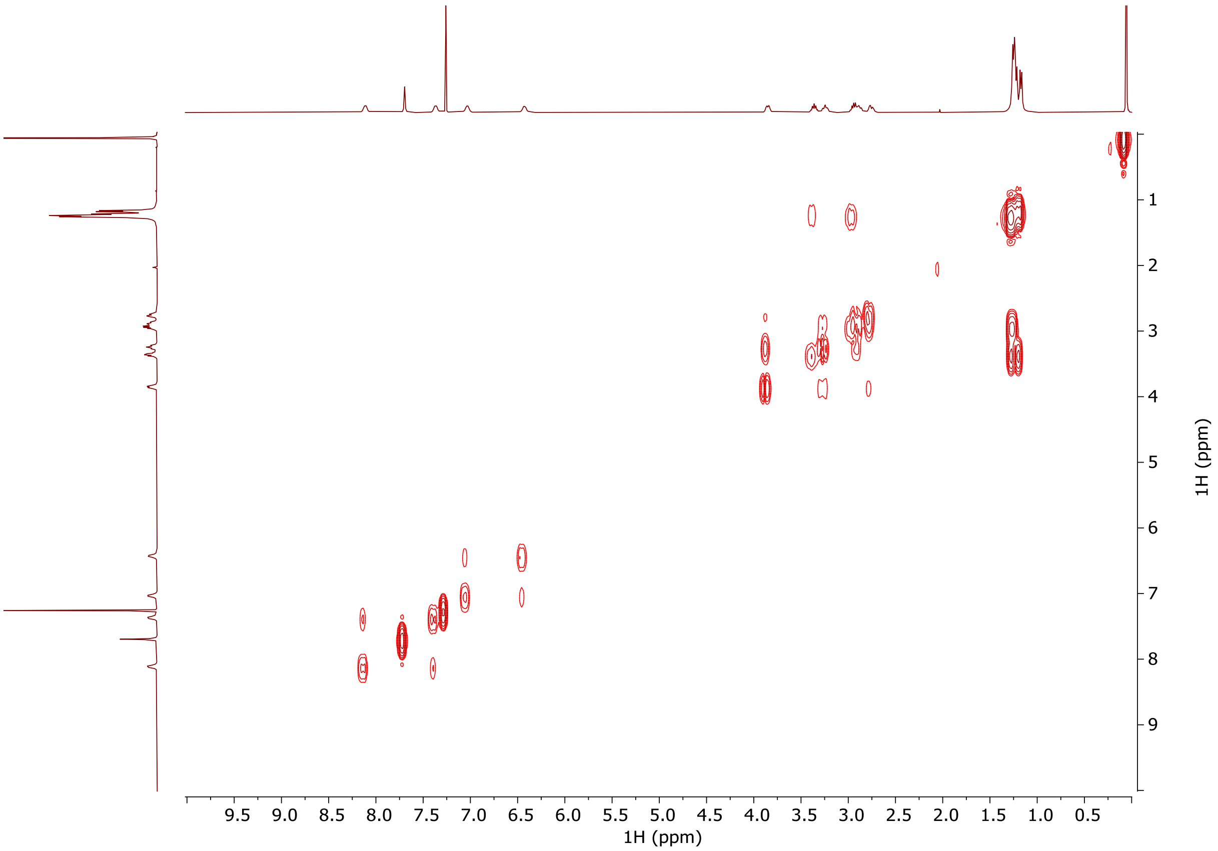


**Figure S28.** $^{1}H$-$^{1}H$ COSY NMR spectrum (400 MHz, $CDCl_3$, 273 K) of cage **7**.

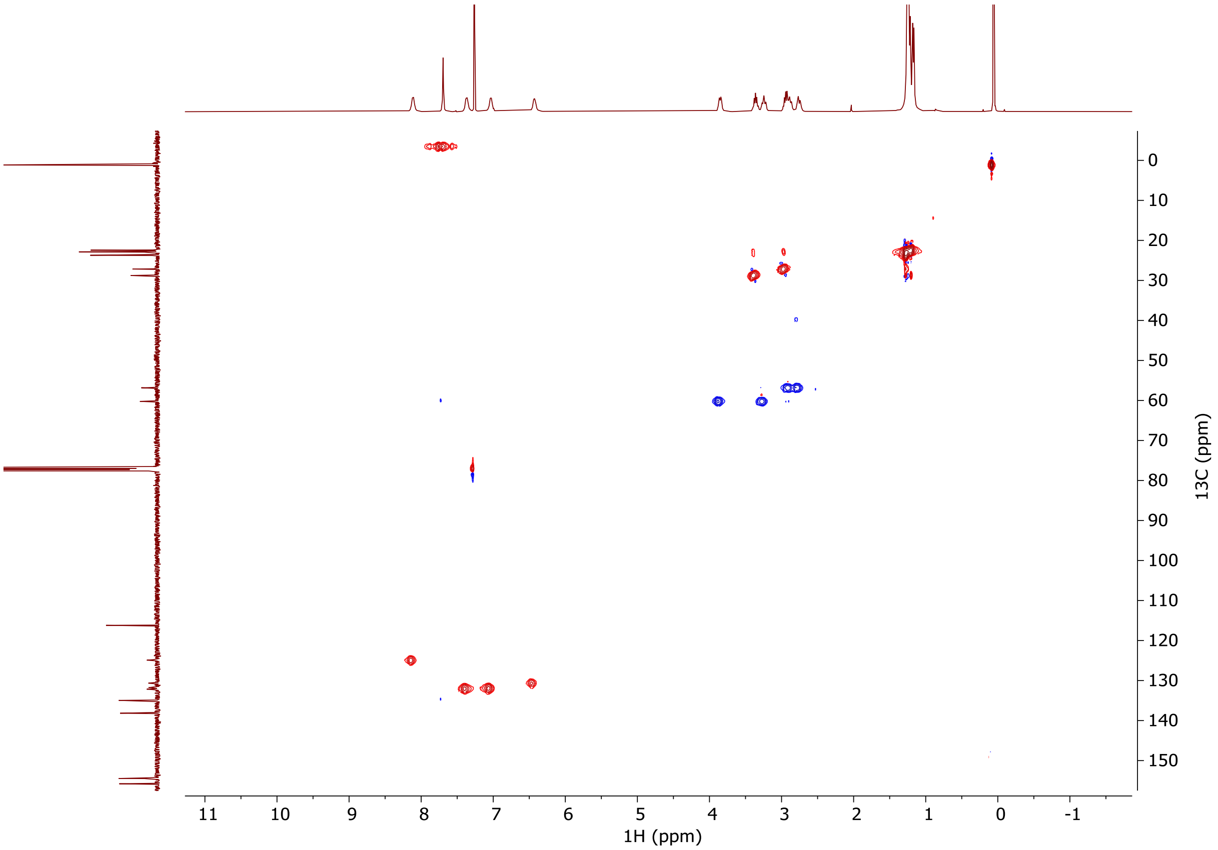


**Figure S29.** $^{1}H$-$^{13}C$ HSQC NMR spectrum (400 MHz, $CDCl_3$, 273 K) of cage **7**.

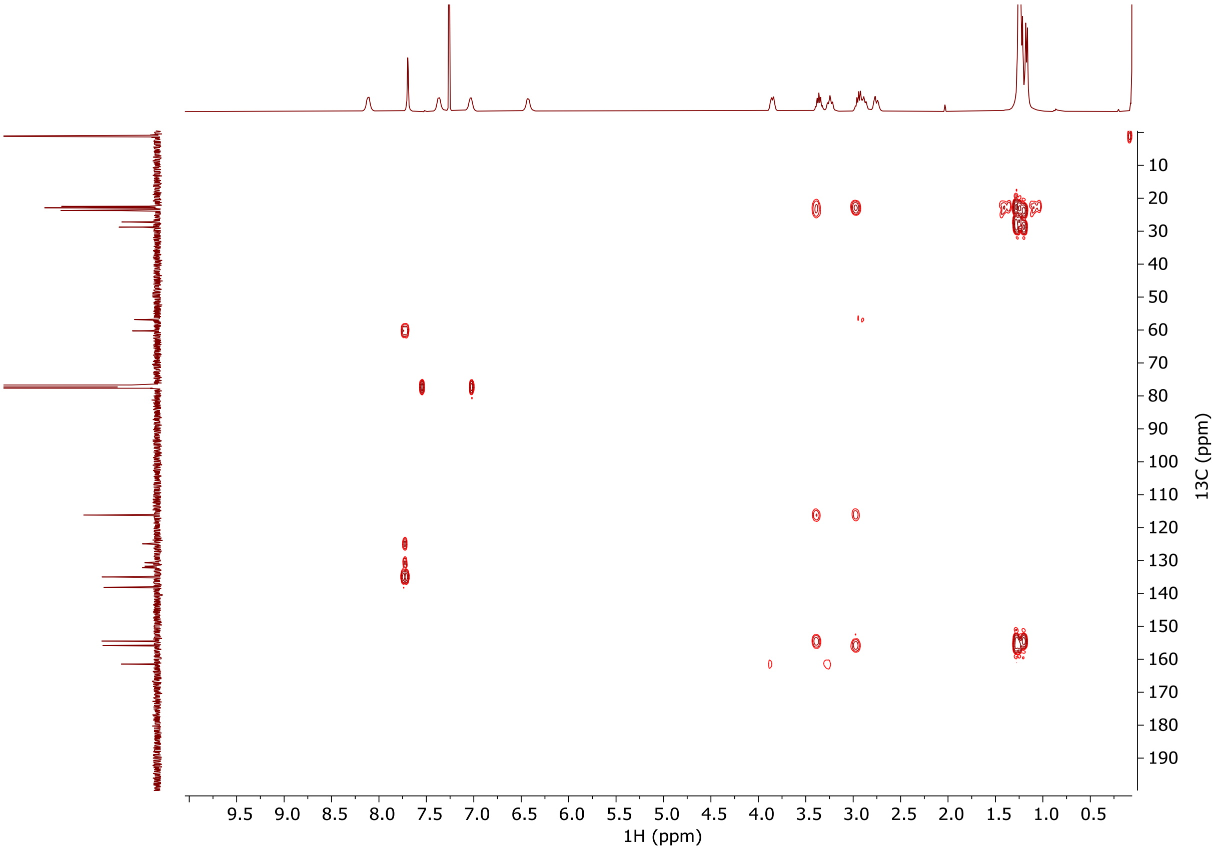


**Figure S30.** $^{1}H$-$^{13}C$ HMBC NMR spectrum (400 MHz, $CDCl_3$, 273 K) of cage **7**.

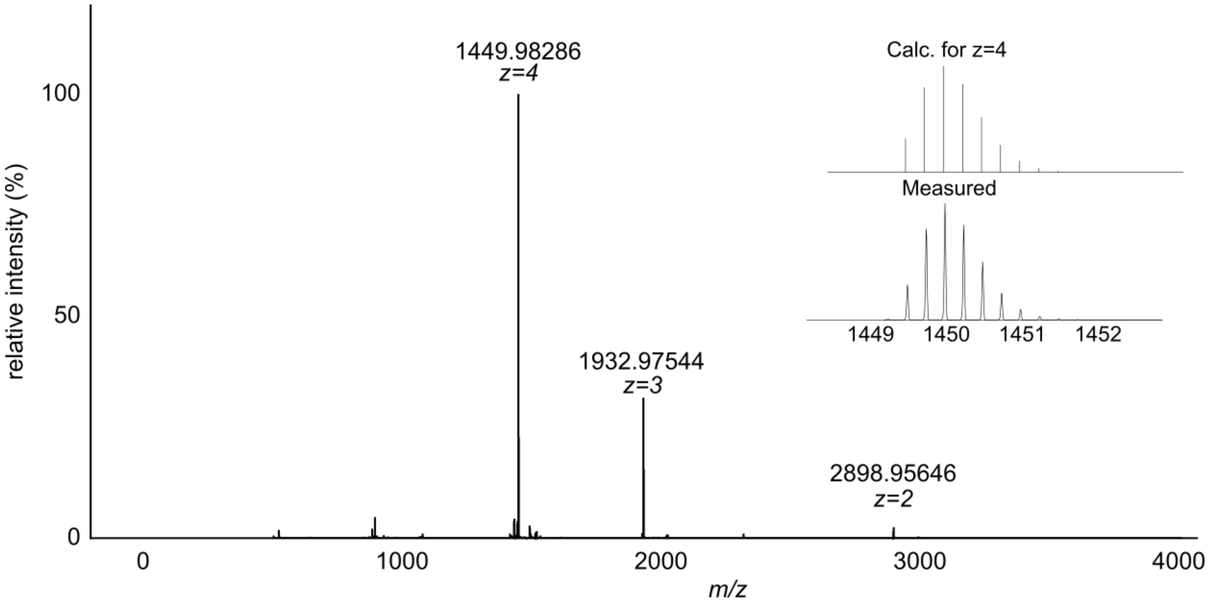


**Figure S31**. ESI-HRMS (positive mode) of cage **7** in $CHCl_3/CH_3CN$ 10:90 %v/v**.** Insert: calculated and measured ESI-HRMS spectrum for $C_{216}H_{280}Au_{12}N_{40}$ [**7**+4H]$^{4+}$.

## 2.6 Screening of different condensation reactions

All reaction were performed on an NMR scale, with 0.5 mL of solvent, 1.6 mM of complex **3** or **4**, and 2.4 mM of the respective diamine or 1.6 mM of tris(2-aminoethyl)amine (TREN). The results are summarized below.

Reactions with complex **3:**

| Amine | Solvent | Result |
|---|---|---|
| *m*-xylylenediamine | $CD_2Cl_2$ | Multiple products |
| | $CDCl_3$ | Multiple products |
| 1,3-diaminopropane | $CD_2Cl_2$ | Clean product |
| | $CDCl_3$ | Clean product |
| (1*R*,2*R*)-(-)-1,2-diaminocyclohexane | $CD_2Cl_2$ | Multiple products, partial conversion |
| | $CDCl_3$ | Multiple products, partial conversion |
| ethylenediamine | $CD_2Cl_2$ | Precipitation |
| | $CDCl_3$ | Precipitation |
| TREN | $CD_2Cl_2$ | Precipitation, multiple products in solution |
| | $CDCl_3$ | Precipitation |

Reactions with complex **4:**

| Amine | Solvent | Result |
|---|---|---|
| *m*-xylylenediamine | $CD_2Cl_2$ | Multiple products |
| | $CDCl_3$ | Multiple products |
| | $C_2D_2Cl_4$ | Clean product |
| 1,3-diaminopropane | $CD_2Cl_2$ | Multiple products |
| | $CDCl_3$ | Multiple products |
| (1*R*,2*R*)-(-)-1,2-diaminocyclohexane | $CD_2Cl_2$ | Multiple products |
| | $CDCl_3$ | Multiple products |
| ethylenediamine | $CD_2Cl_2$ | Multiple products, partial conversion |
| | $CDCl_3$ | Partial conversion |
| TREN | $CD_2Cl_2$ | Precipitation, clean product in solution |
| | $CDCl_3$ | Clean product |

# 3 Host-guest chemistry

## 3.1 $C_{60}$⊂7

$C_2D_2Cl_4$ was passed throw basic $Al_2O_3$. A stock solution of $C_{60}$ (3.1 mg, 4.31 µmol, 5 eq.) in $C_2D_2Cl_4$ (1 mL) and a stock solution of cage **7** (16 mg, 2.76 µmol, 3.2 eq.) in $C_2D_2Cl_4$ (0.96 mL) was prepared. 300 µL from each solution were combined in an NMR tube and the reaction was followed by NMR. Complete conversion to $C_{60}$⊂7 was observed after 12 h.

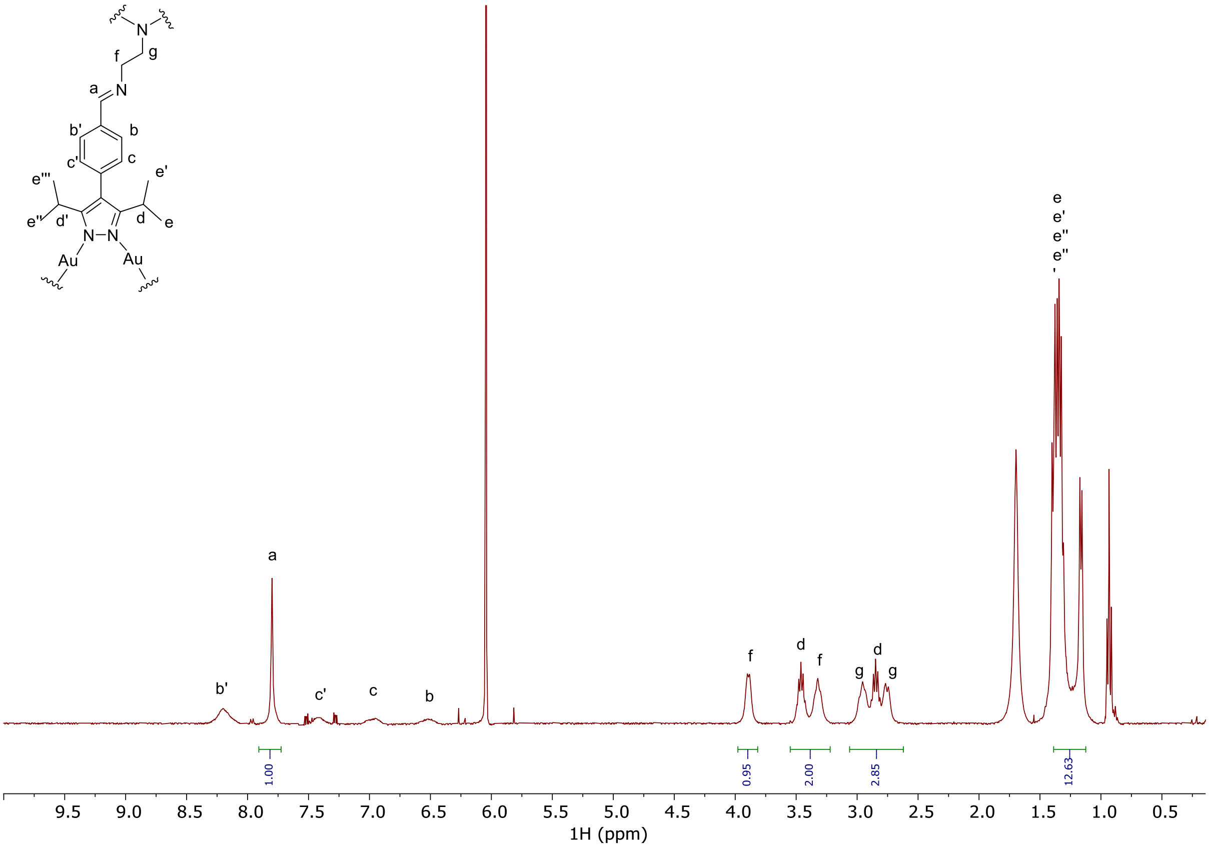


**Figure S32.** $^1$H NMR spectrum (400 MHz, $C_2D_2Cl_4$, 298 K) of **$C_{60}$⊂7**.

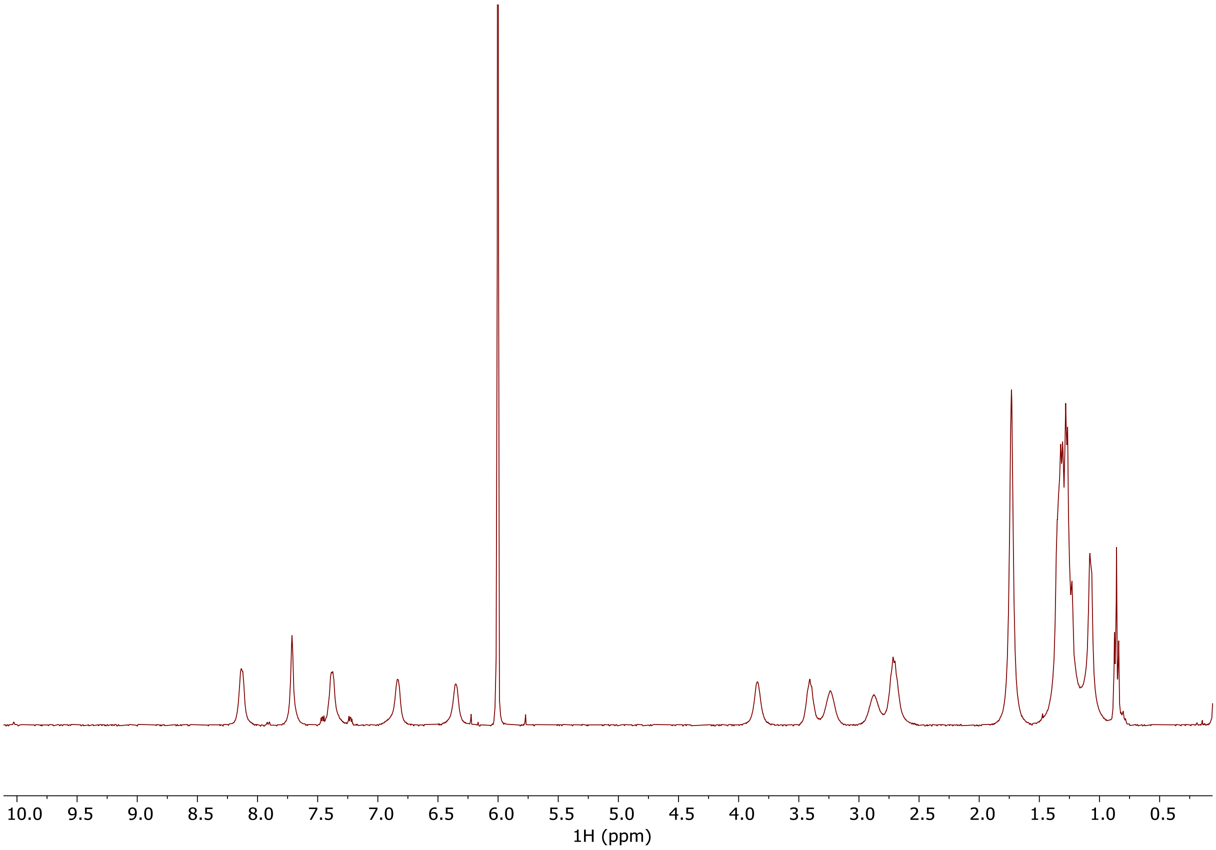


**Figure S33.** $^{1}H$ NMR spectrum (400 MHz, $C_2D_2Cl_4$, 273 K) of **$C_{60}$⊂7**.

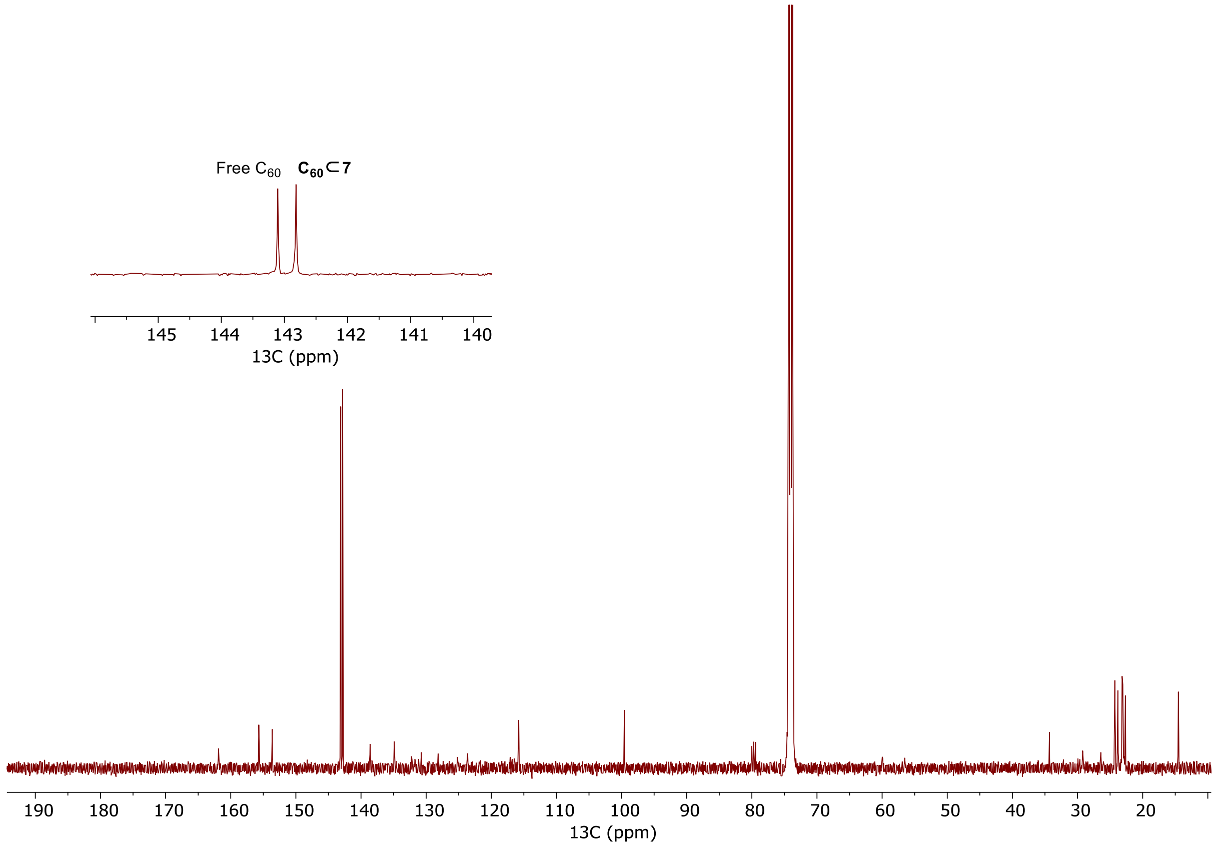


**Figure S34.** $^{13}C$ NMR spectrum (101 MHz, $C_2D_2Cl_4$, 273 K) of **$C_{60}$⊂7**.

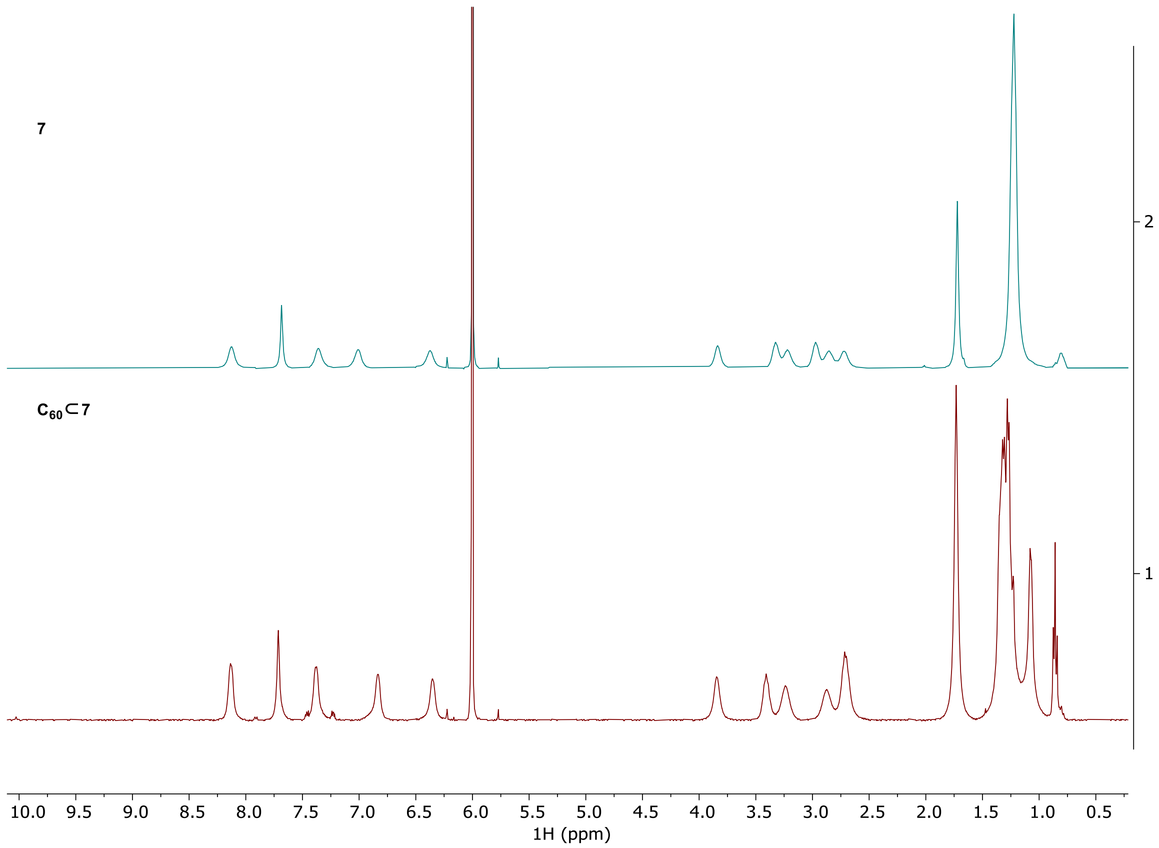


**Figure S35.** Stack plot of $^{1}$H NMR spectra (400 MHz, $C_2D_2Cl_4$, 273 K) of **7** and **$C_{60}$⊂7.**

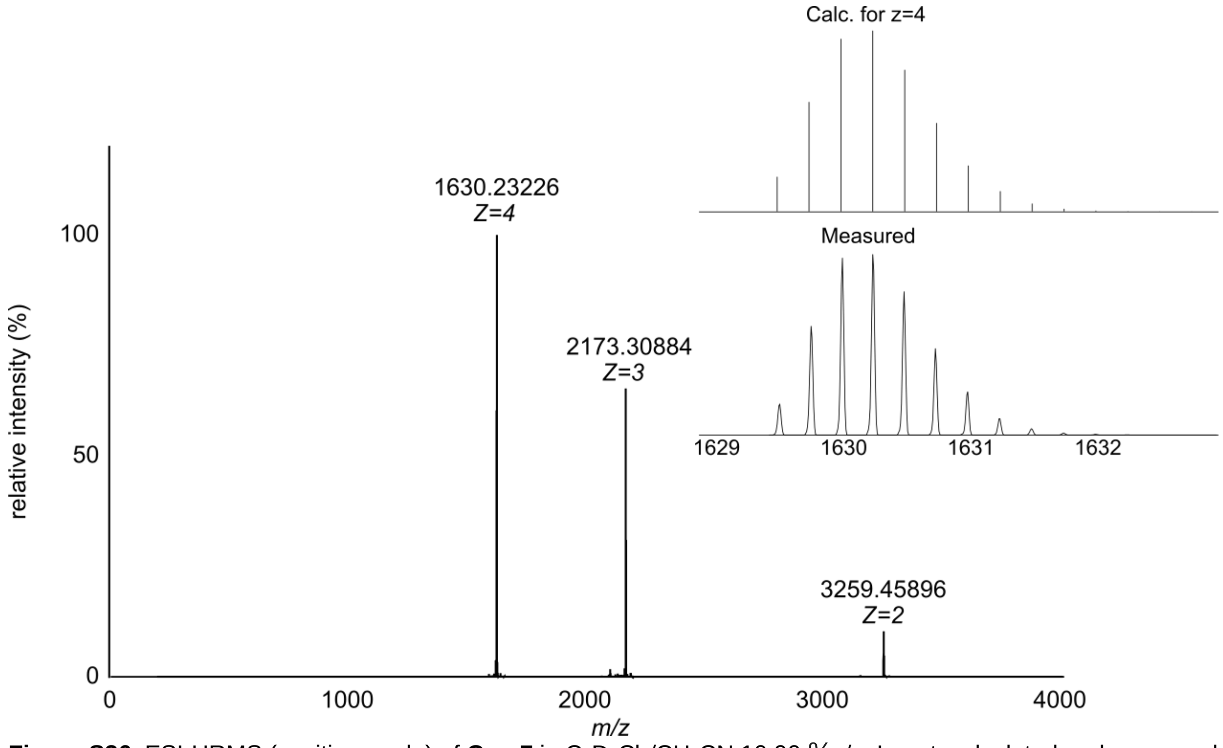


**Figure S36.** ESI-HRMS (positive mode) of **$C_{60}$⊂7** in $C_2D_2Cl_4/CH_3CN$ 10:90 %v/v. Insert: calculated and measured ESI-HRMS spectrum for $C_{276}H_{280}Au_{12}N_{40}$ [**$C_{60}$⊂7** +4H]$^{4+}$.

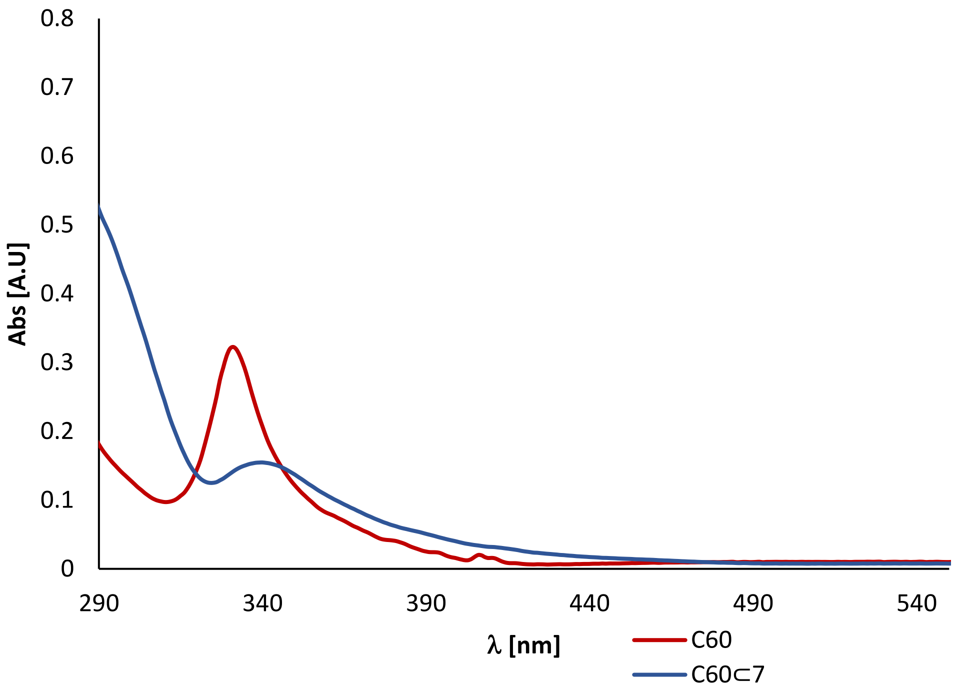


**Figure S37.** UV-vis spectra (298 K) of $C_{60}$ (4 µM) and of **$C_{60}$⊂7** (4 µM) in $C_2H_2Cl_4$.

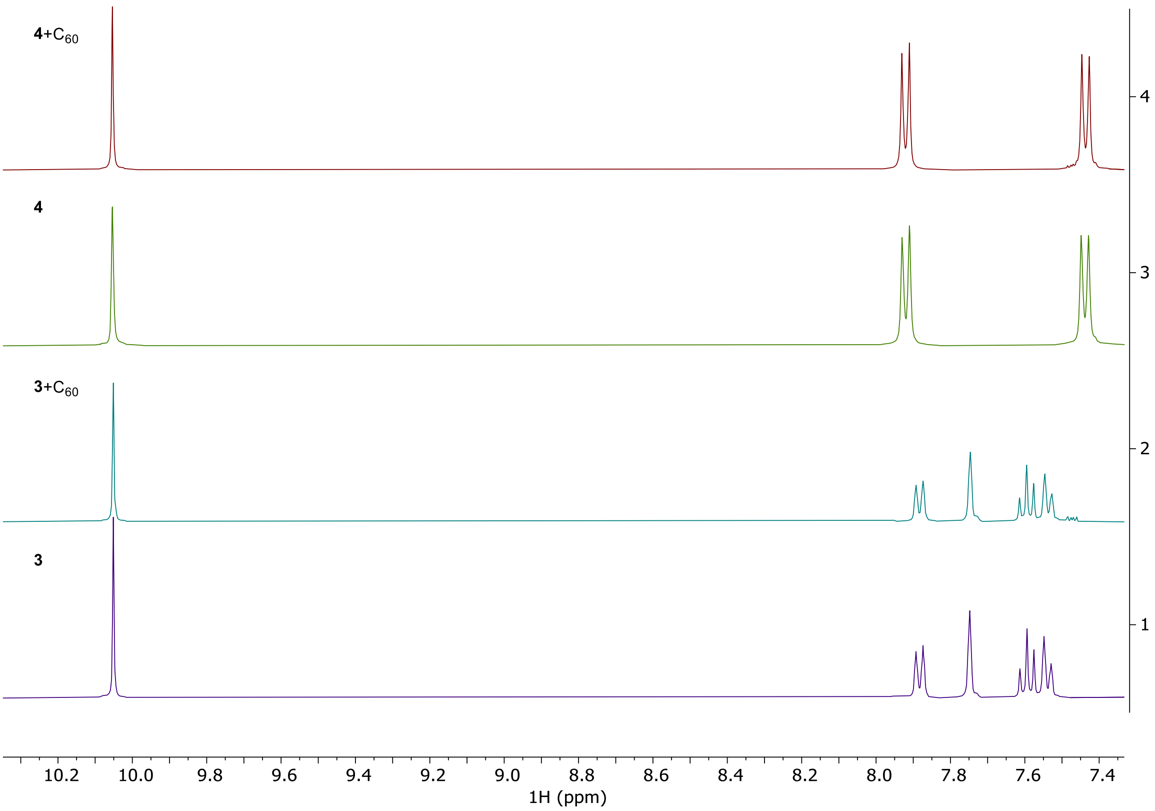


**Figure S38**. Partial $^1$H-NMR spectra (400 MHz, $C_2D_2Cl_4$, 298 K) of complex **3** (1 mM), complex **3** with $C_{60}$ (5 mM), complex **4** (1 mM), and complex **4** with $C_{60}$ (5 mM)**.** An interaction between complexes **3** and **4** and the fullerene cannot be detected.

## 3.2 $C_{70}$⊂7

$C_2D_2Cl_4$ was passed through basic $Al_2O_3$. A stock solution of $C_{70}$ (3.6 mg, 4.31 µmol, 5 eq.) in $C_2D_2Cl_4$ (1 mL) and a stock solution of cage **7** (16 mg, 2.76 µmol, 3.2 eq.) in $C_2D_2Cl_4$ (0.96 mL) was prepared. 300 µL from each solution were combined in an NMR tube and the reaction was followed by NMR. Complete conversion to **$C_{70}$⊂7** was observed after 12 h.

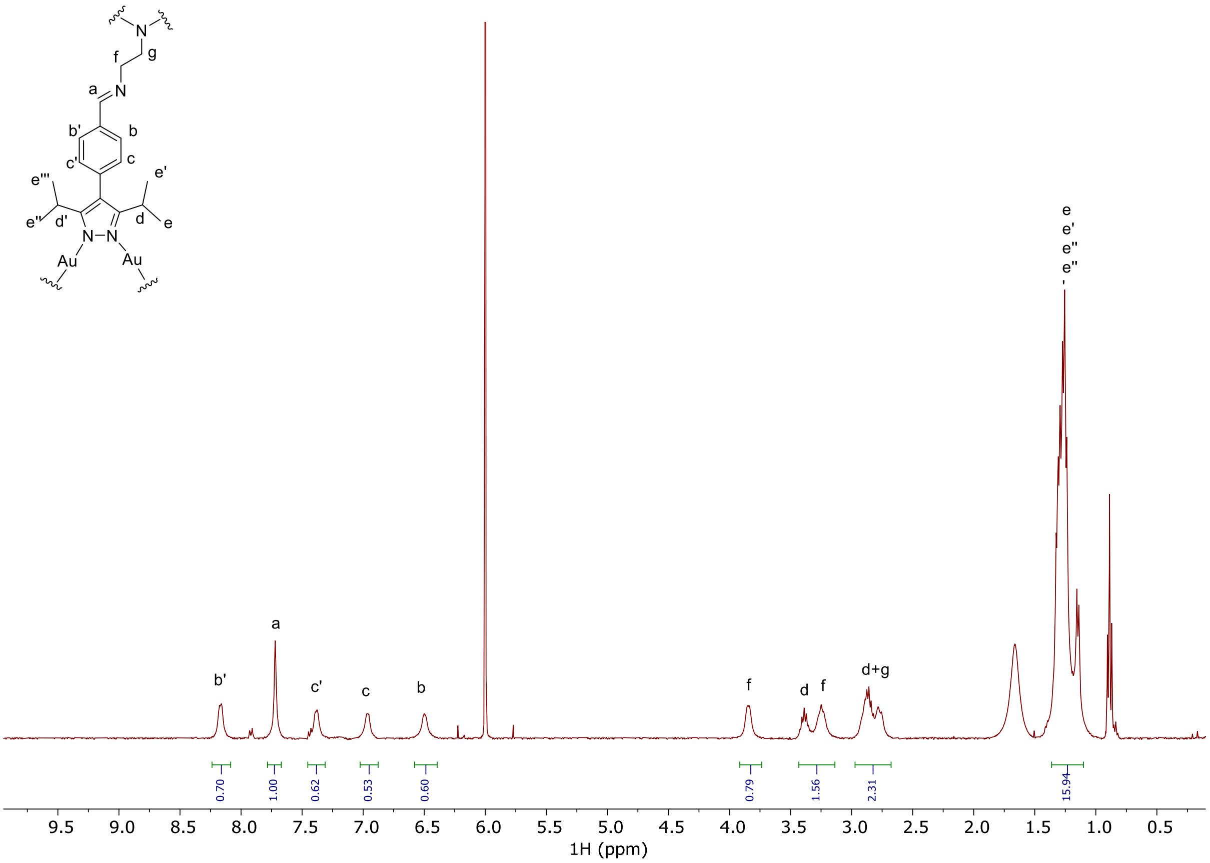


**Figure S39.** $^{1}$H NMR spectrum (400 MHz, $C_2D_2Cl_4$, 298 K) of **$C_{70}$⊂7**.

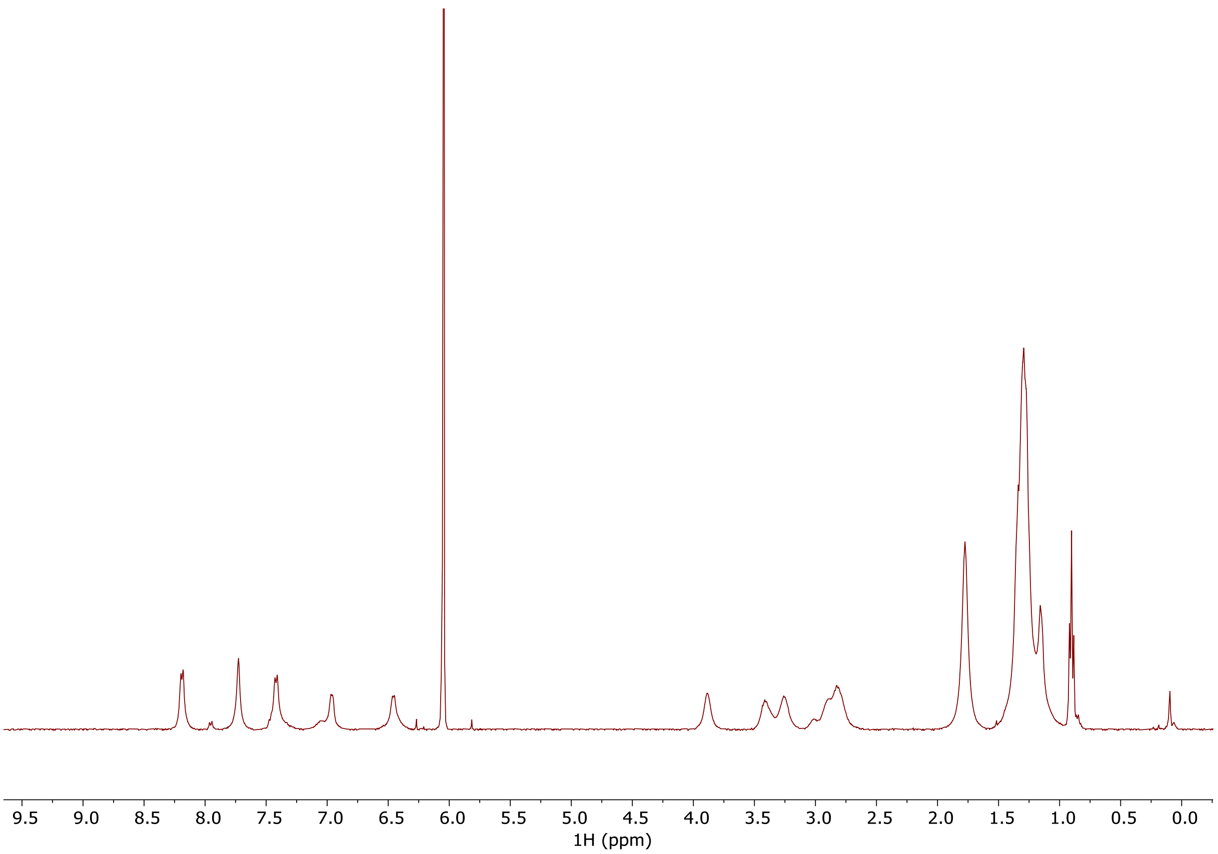


**Figure S40.** $^{1}H$ NMR spectrum (400 MHz, $C_2D_2Cl_4$, 273 K) of **$C_{70}\subset$7**.

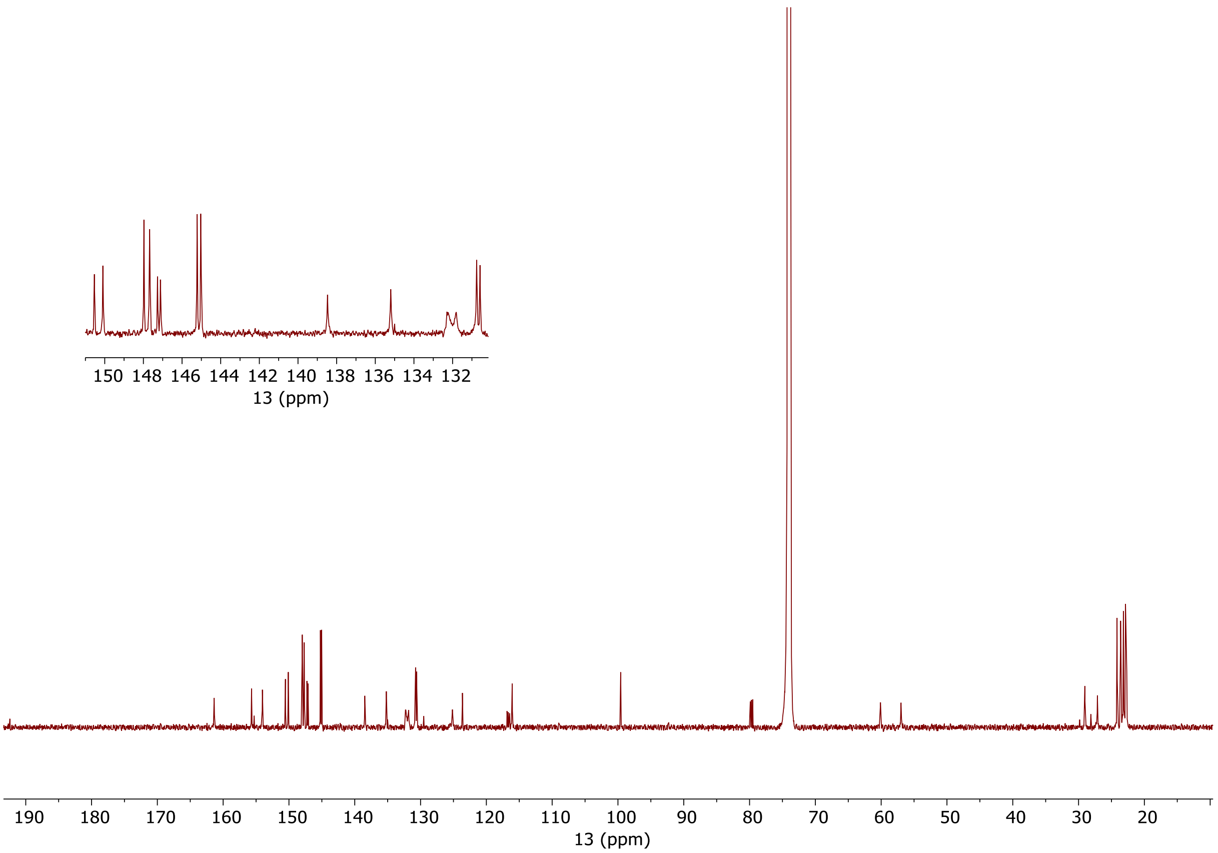


**Figure S41.** $^{13}C$ NMR spectrum (150 MHz, $C_2D_2Cl_4$, 298 K) of **$C_{70}\subset$7**. Two sets of signals for all 5 $C_{70}$ peaks are showed in the insert.

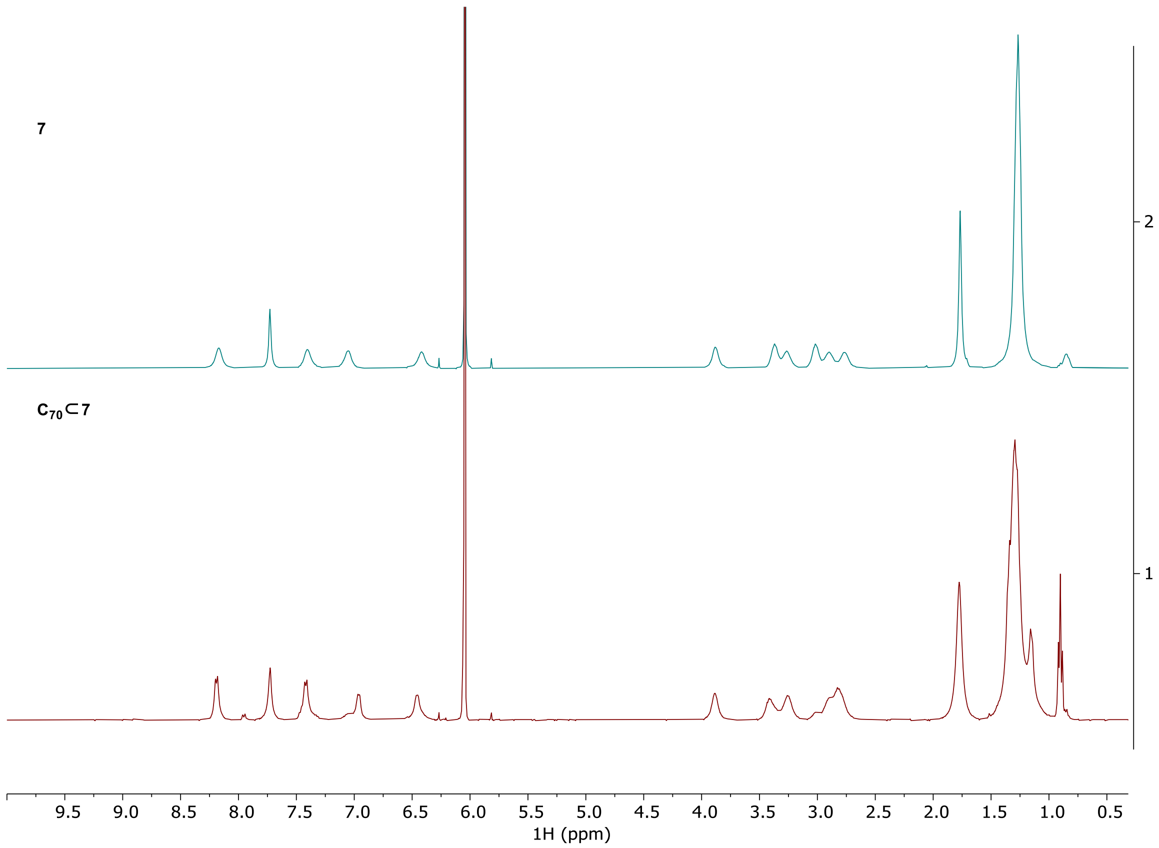


**Figure S42.** Stack plot of $^{1}$H NMR spectra (400 MHz, $C_2D_2Cl_4$, 273 K) of **7** and **$C_{70}$⊂7.**

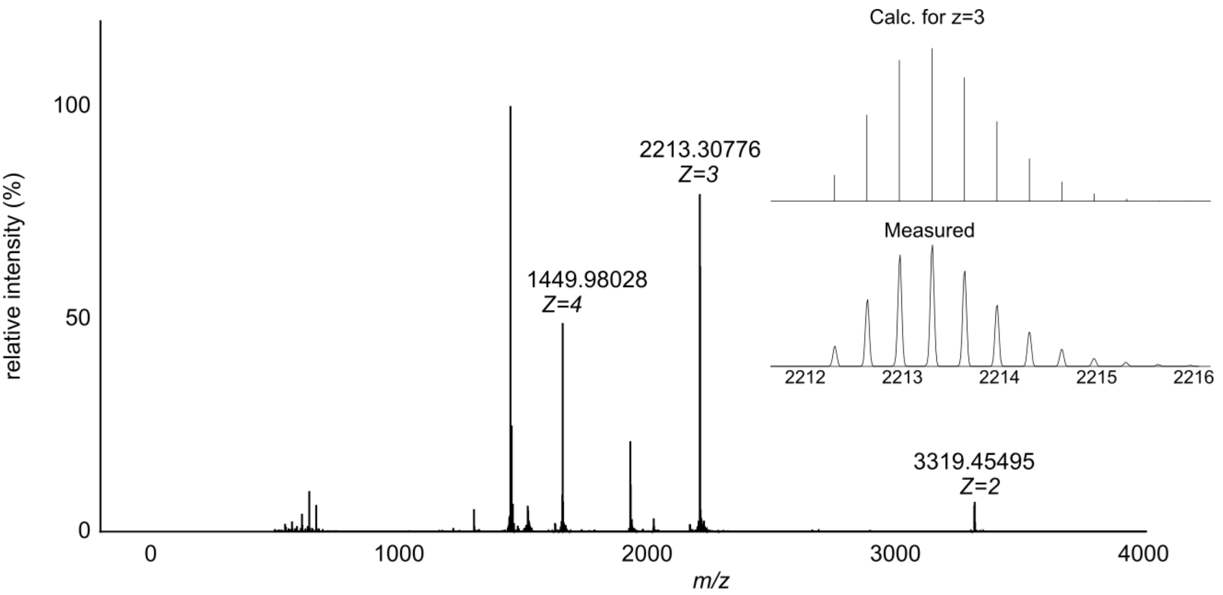


**Figure S43.** ESI-HRMS (positive mode) of **$C_{70}$⊂7** in $C_2D_2Cl_4/CH_3CN$, 10:90 %v/v. Insert: calculated and measured ESI-HRMS spectrum for $C_{286}H_{279}Au_{12}N_{40}$ [**$C_{70}$⊂7** +3H]$^{3+}$.

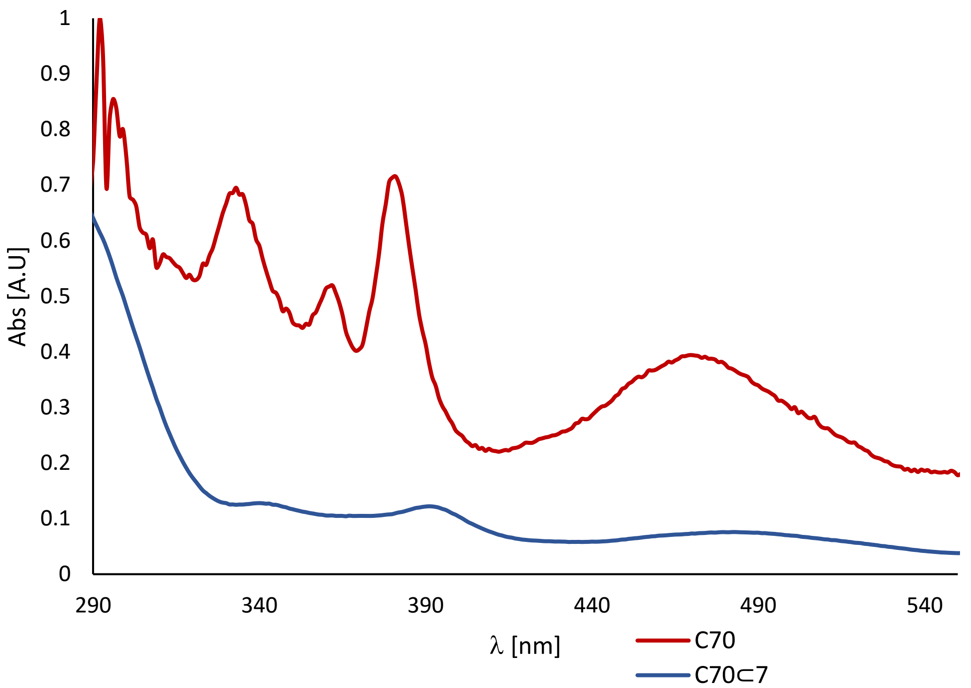


**Figure S44.** UV-vis spectra (298 K) of $C_{70}$ (4 µM) and **$C_{70}$⊂7** (4 µM) in $C_2H_2Cl_4$.

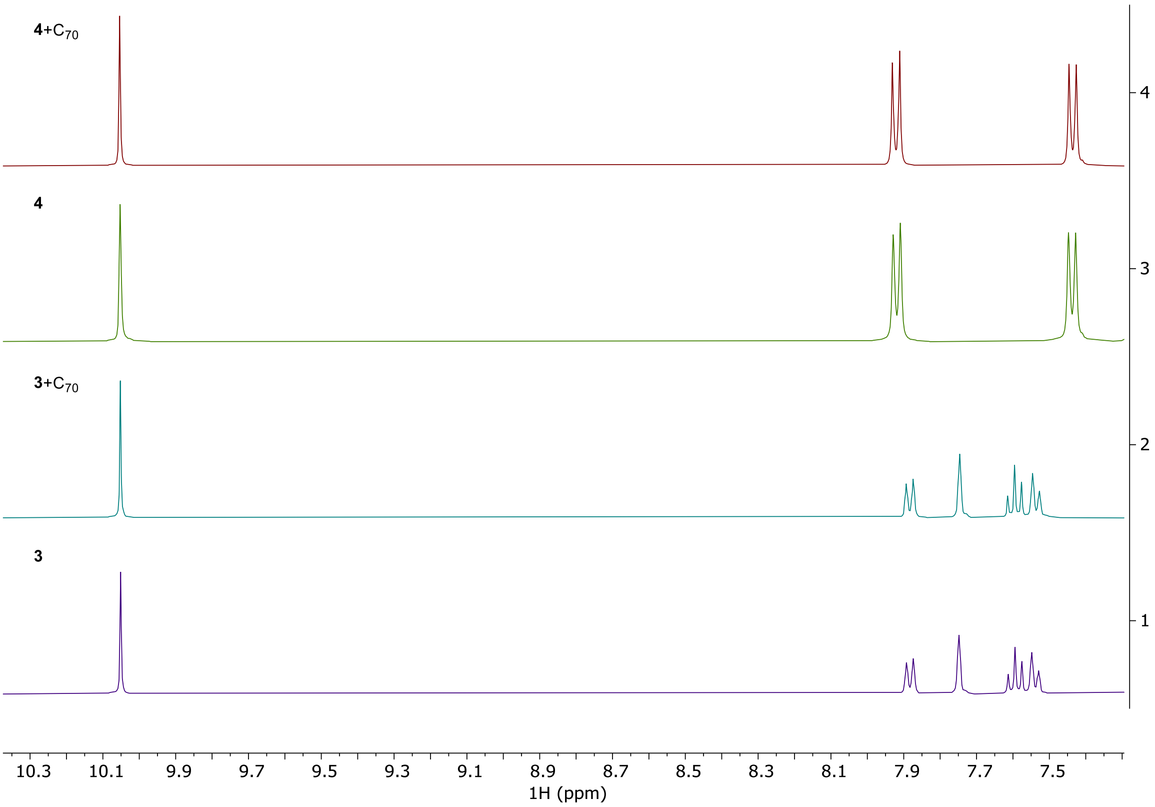


**Figure S45.** Partial $^1$H-NMR spectra (400 MHz, $C_2D_2Cl_4$, 298 K) of complex **3** (1 mM), complex **3** with $C_{70}$ (5 mM), complex **4** (1 mM), and complex **4** with $C_{70}$ (5 mM)**.** An interaction between complexes **3** and **4** and the fullerene cannot be detected.

## 3.3 Competition experiment

$C_2D_2Cl_4$ (300 µL) was added to the respective fullerene ($C_{60}$: 541 µg, 0.751 µmol; $C_{70}$: 635 µg, 0.755 µmol). The suspensions were sonication for 5 min and then equilibrated for 24 h, resulting in clear solutions. An aliquot of the solution of $C_{60}$ (276 µL) and an aliquot of the solution of $C_{70}$ (274 µL) was added to a vial containing cage **7** (3.99 mg, 0.688 µmol), resulting in a 1:1:1 mixture. After equilibration for 3 h, the solution was analyzed by $^1$H NMR spectroscopy.

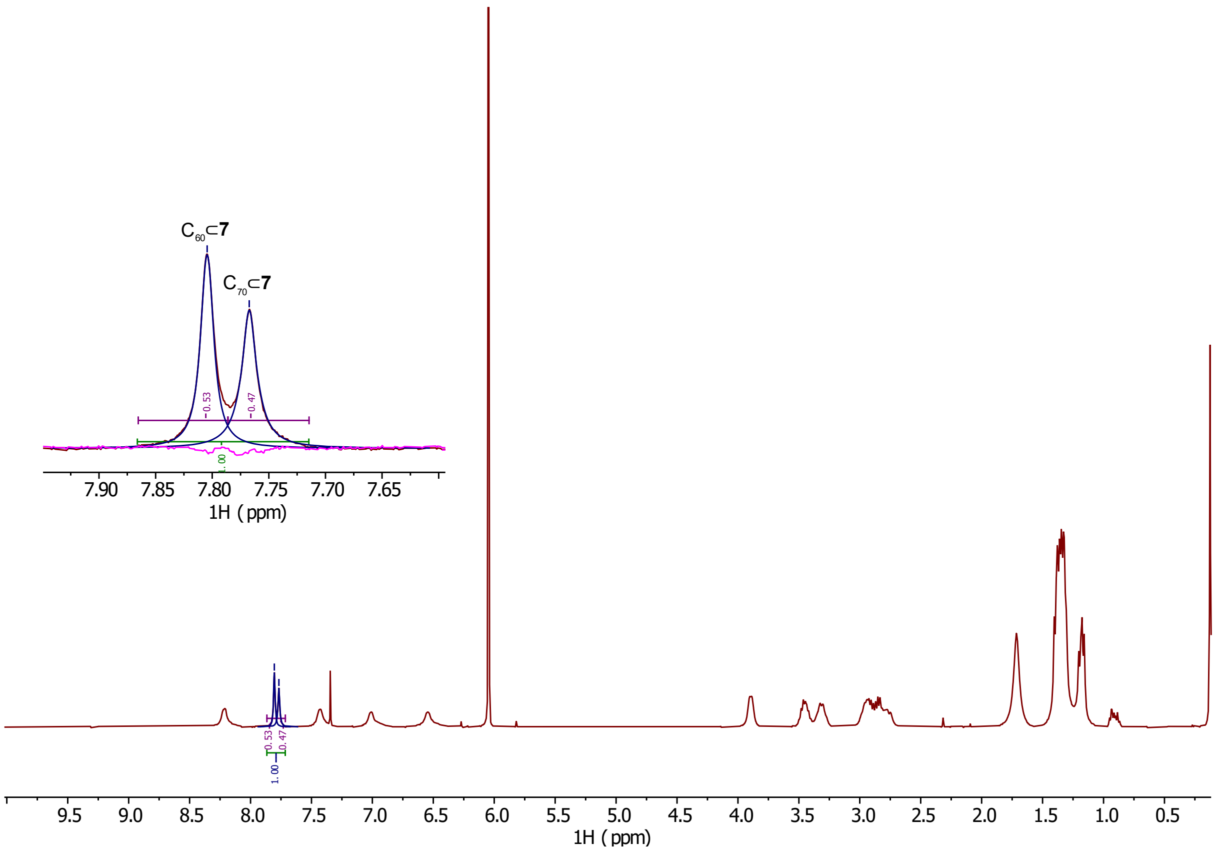


**Figure S46.** $^1$H NMR spectrum (400 MHz, $C_2D_2Cl_4$, 298 K) of a 1:1:1 mixture of cage **7**, $C_{60}$, and $C_{70}$ after equilibration for 3 h. Insert: zoom in on the imine peaks. blue: deconvolution of the peak, pink: peaks residual.

## 3.4 Host-guest chemistry with the cages 5 and 6

All host-guest studies for cages **5** and **6**, as well as the control experiments with compounds **3** and **4** were performed in $CD_2Cl_2$ for comparison reasons, regardless of the solvent used for characterization.

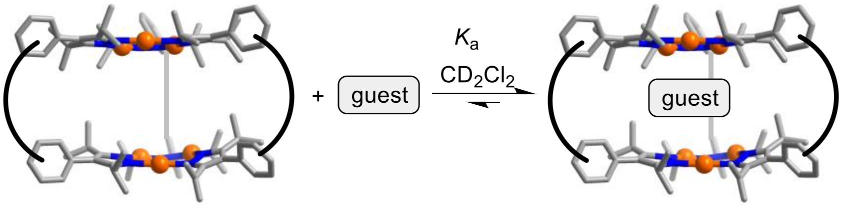


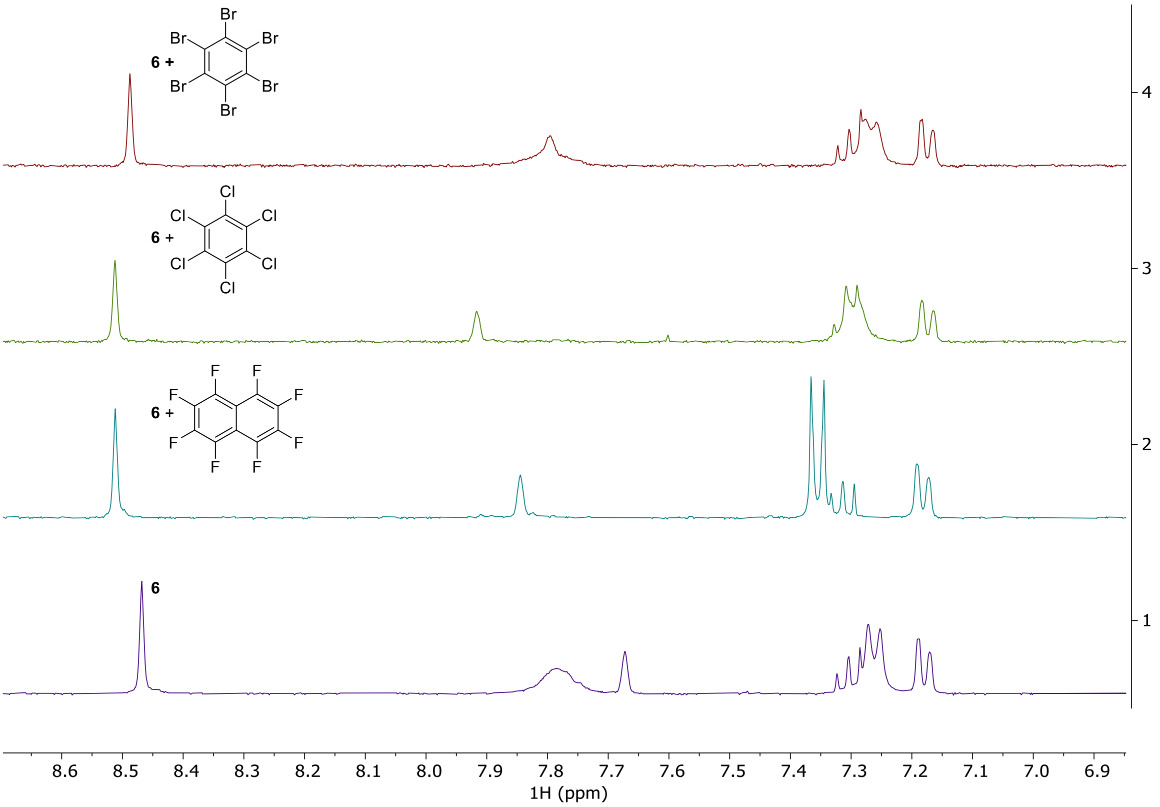


**Figure S47.** Aromatic parts of the $^1$H-NMR spectra (400 MHz, $CD_2Cl_2$, 298 K) of cage **6** (0.5 mM) and of cage **6** with octafluoronaphthalene (5 mM), with hexachlorobenzene (5 mM), or with hexabromobenzene (5 mM). The differences of the chemical shifts indicate binding of the halogenated compounds.

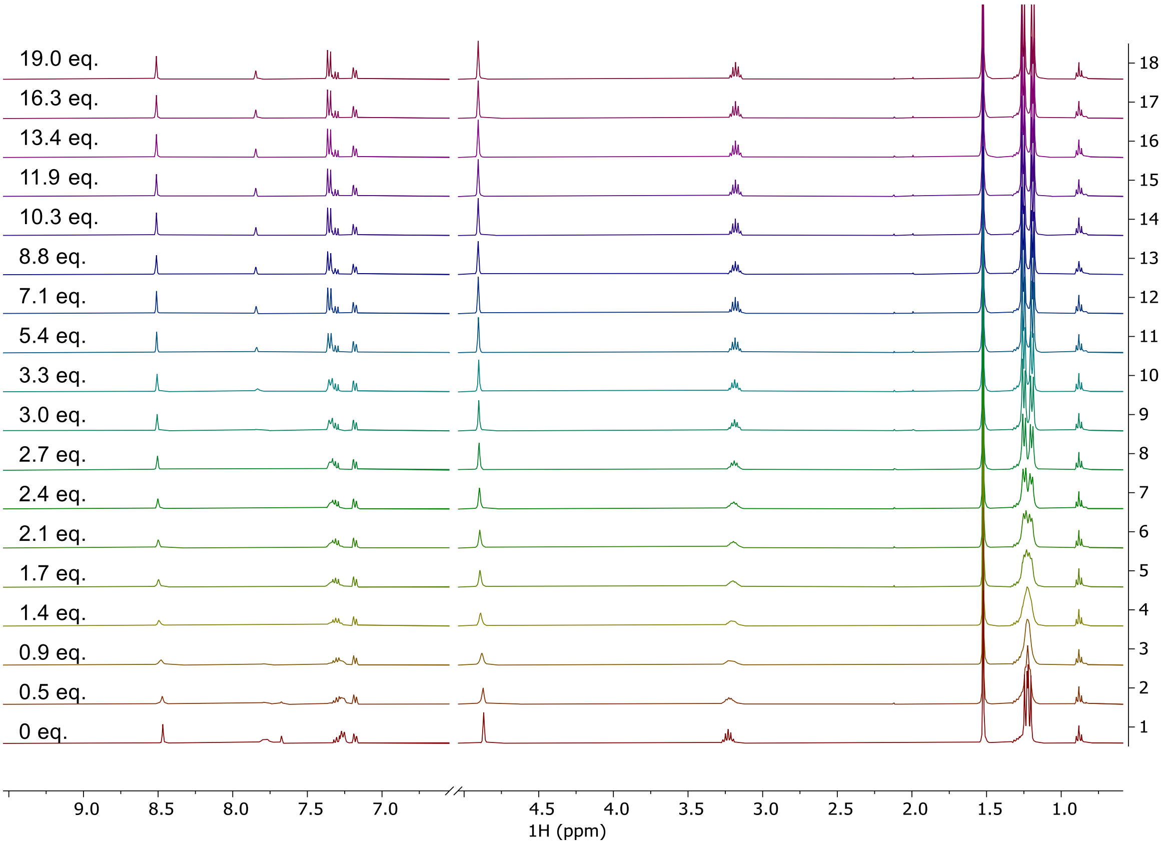


**Figure S48.** NMR titration of cage **6** with octafluoronaphthalene (400 MHz, $CD_2Cl_2$, 298 K). The peak at 8.5 ppm was used for fitting (see Figure S47).

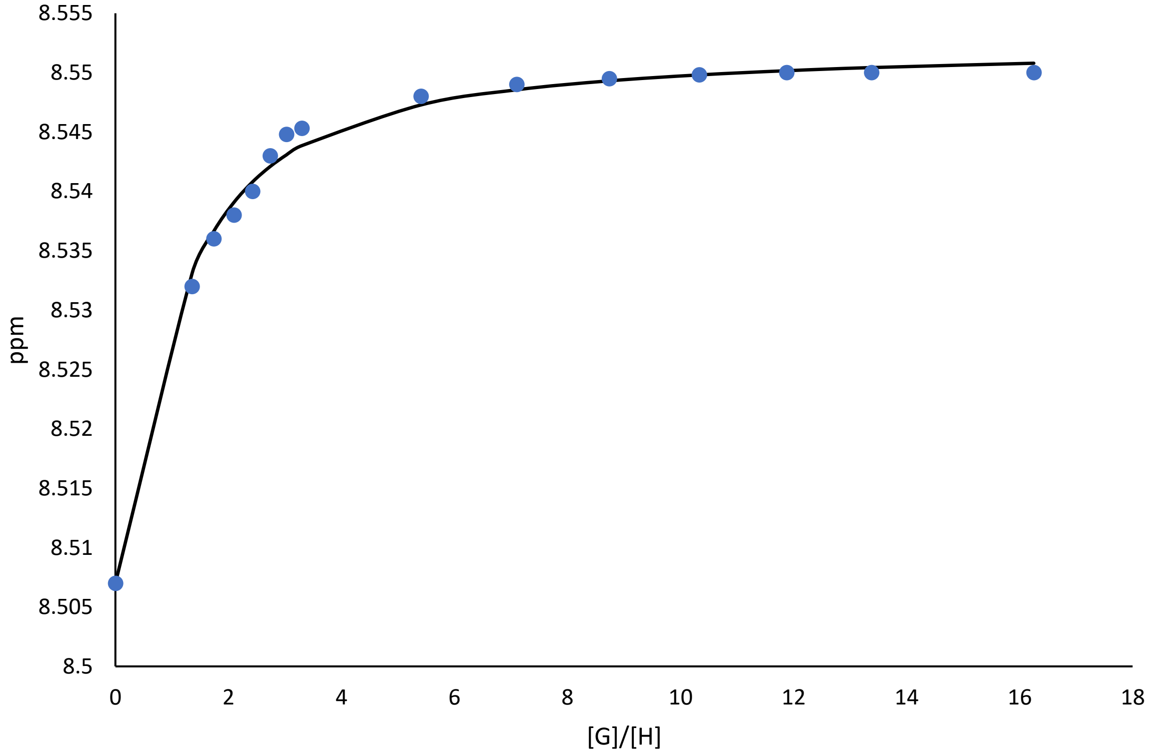


**Figure S49.** Binding isotherm for a titration of cage **6** with octafluoronaphthalene. Fitting of the isotherm gave an apparent association constant of $K_a = 4.0 \pm 0.4 \times 10^3$ $M^{-1}$.

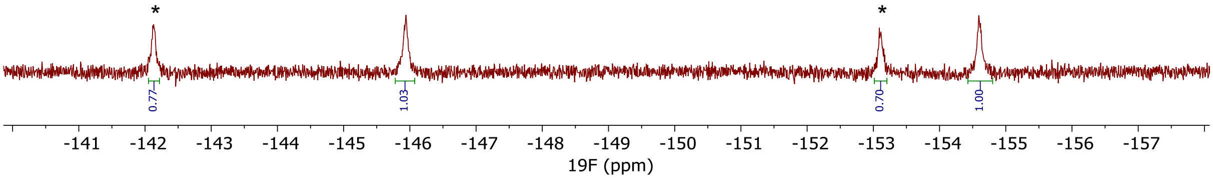


**Figure S50.** $^{19}$F-NMR spectrum (376 MHz, $CD_2Cl_2$, 298 K) of a mixture of cage **6** (0.3 mM) and octafluoronaphthalene (0.46 mM). The peaks corresponding to the bound guest are labelled with an asterisk. By integration, we were able to derive apparent binding constants of $K_a = 7.3{\times}10^3$ $M^{-1}$ (signals at –142 and –146 ppm) and $K_a = 6.3{\times}10^3$ $M^{-1}$ (signals at –153 and –155 ppm). The average value is $K_a = 6.8{\times}10^3$ $M^{-1}$ with an estimated error of 10%.

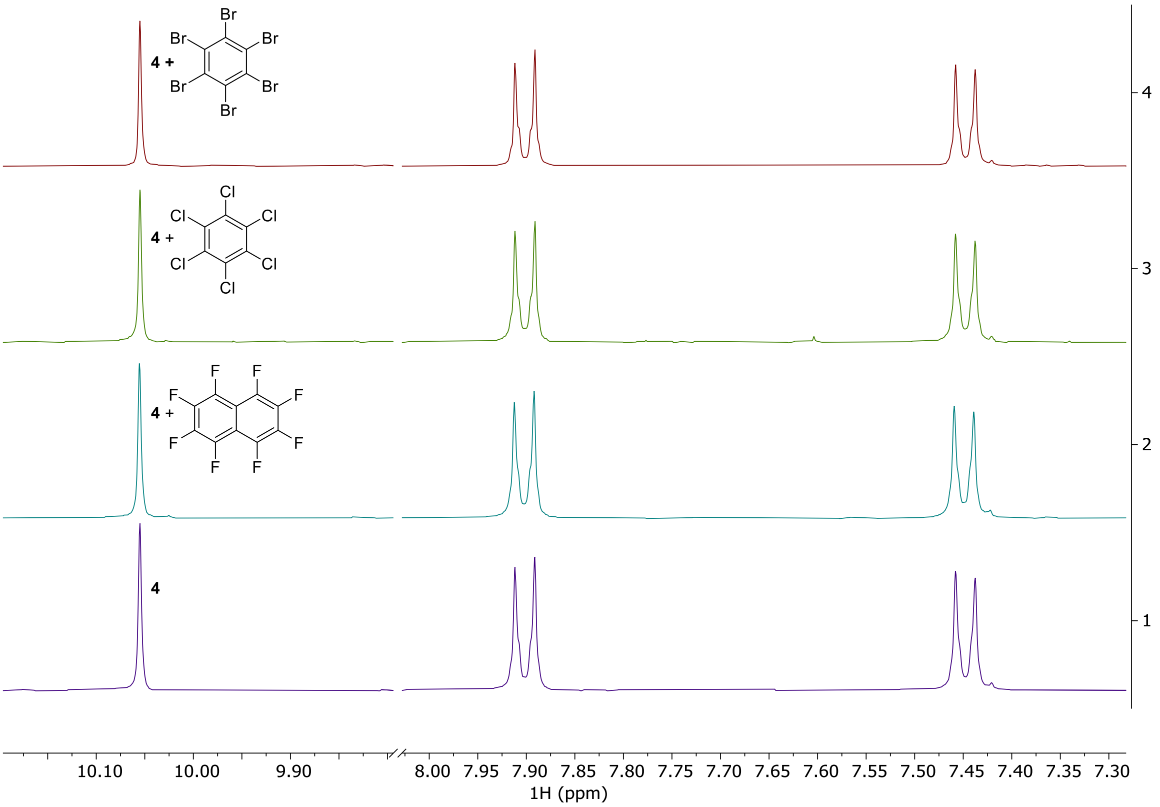


**Figure S51.** $^{1}$H-NMR spectra (400 MHz, $CD_2Cl_2$, 298 K) of complex **4** (0.5 mM) and of complex **4** with octafluoronaphthalene (5 mM), with hexachlorobenzene (5 mM), or with hexabromobenzene (5 mM). An interaction between complex **4** and the halogenated compounds cannot be detected.

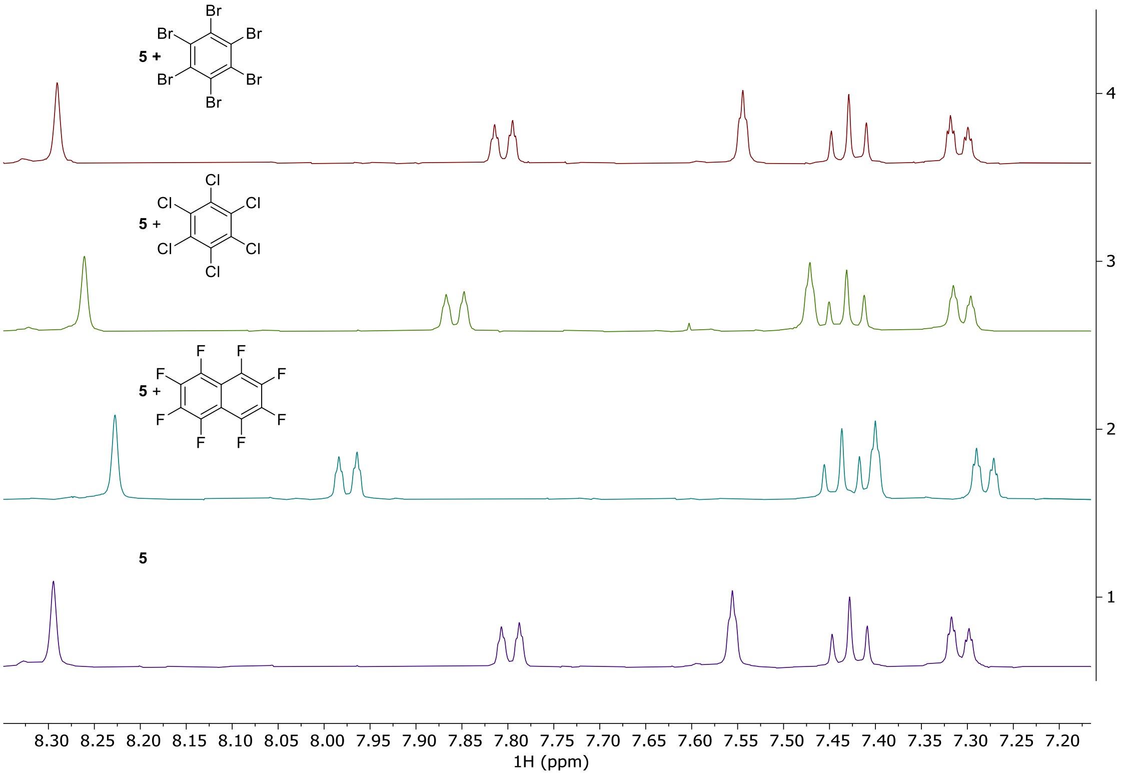


**Figure S52.** $^{1}$H-NMR spectra (400 MHz, $CD_2Cl_2$, 298 K) of cage **5** (1 mM) and of cage **5** with octafluoronaphthalene (5 mM), with hexachlorobenzene (5 mM), or with hexabromobenzene (5 mM). The differences of the chemical shifts indicate binding of the halogenated compounds.

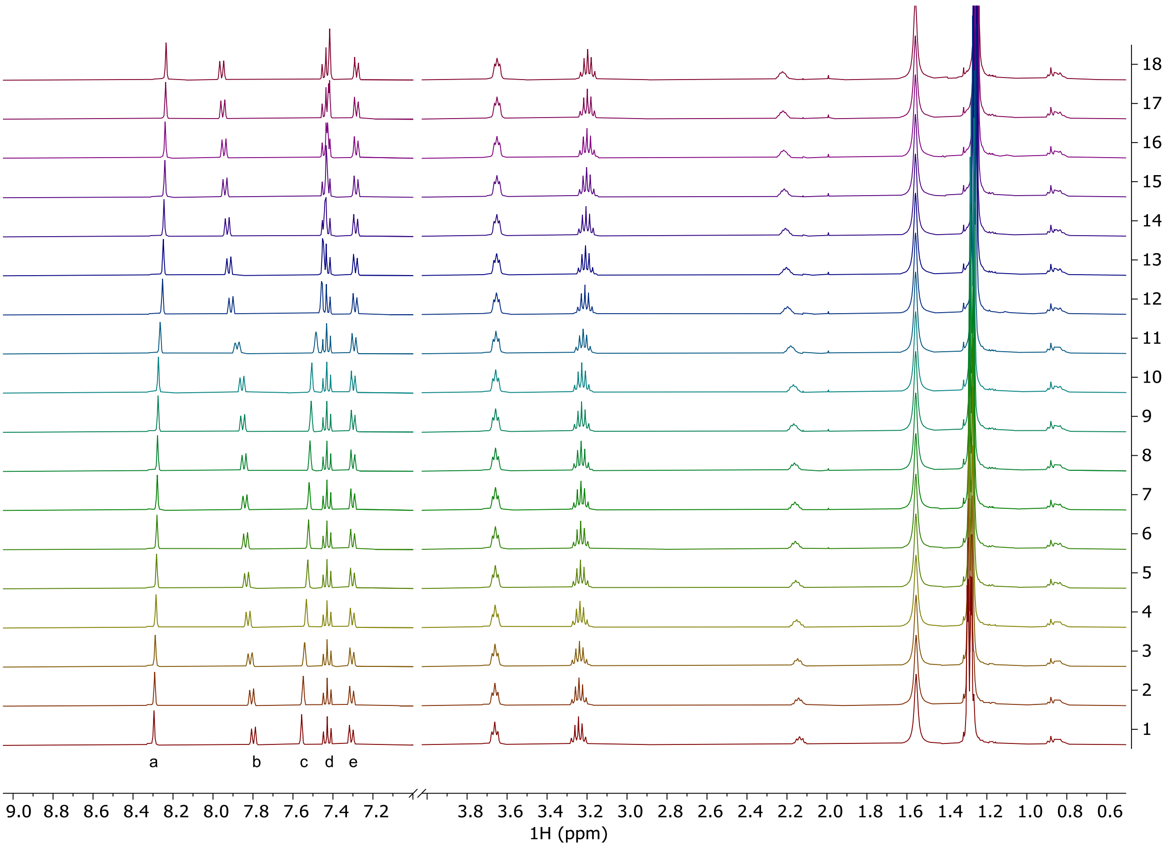


**Figure S53.** NMR titration of **5** with octafluoronaphthalene (400 MHz, $CD_2Cl_2$, 298 K). The peaks labeled **a**–**e** were used for fitting (see Figure S52).

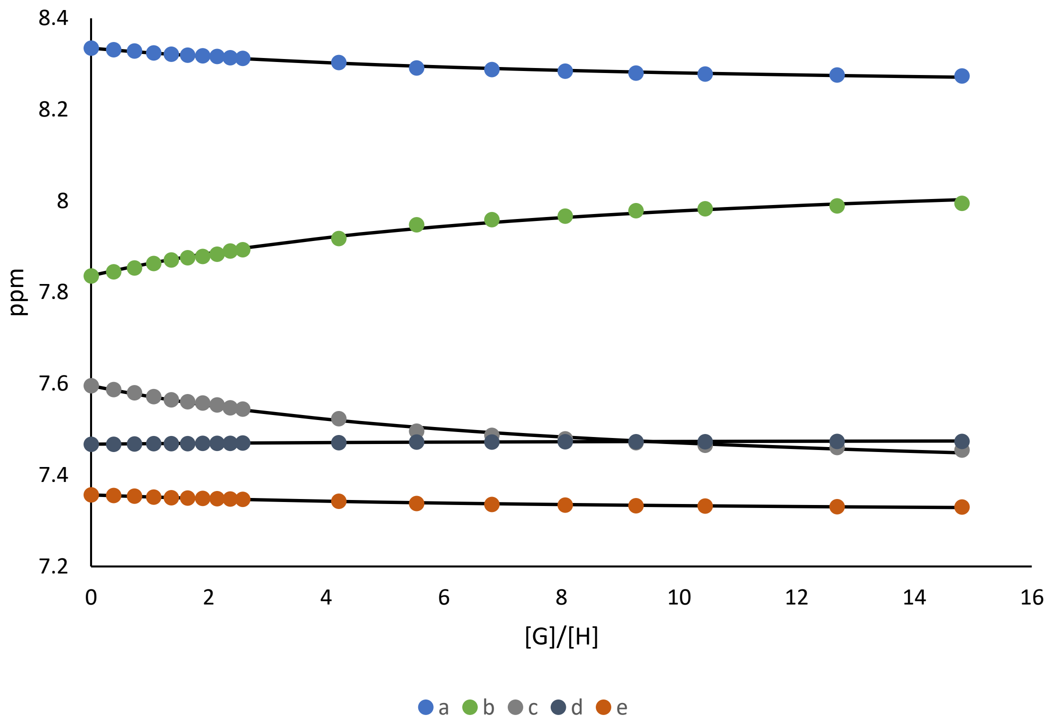


**Figure S54.** Binding isotherms for a titration of cage **5** with octafluoronaphthalene. Fitting of the isotherm gave an apparent association constant of $K_a = 3.0 \pm 0.1 \times 10^2$ $M^{-1}$.

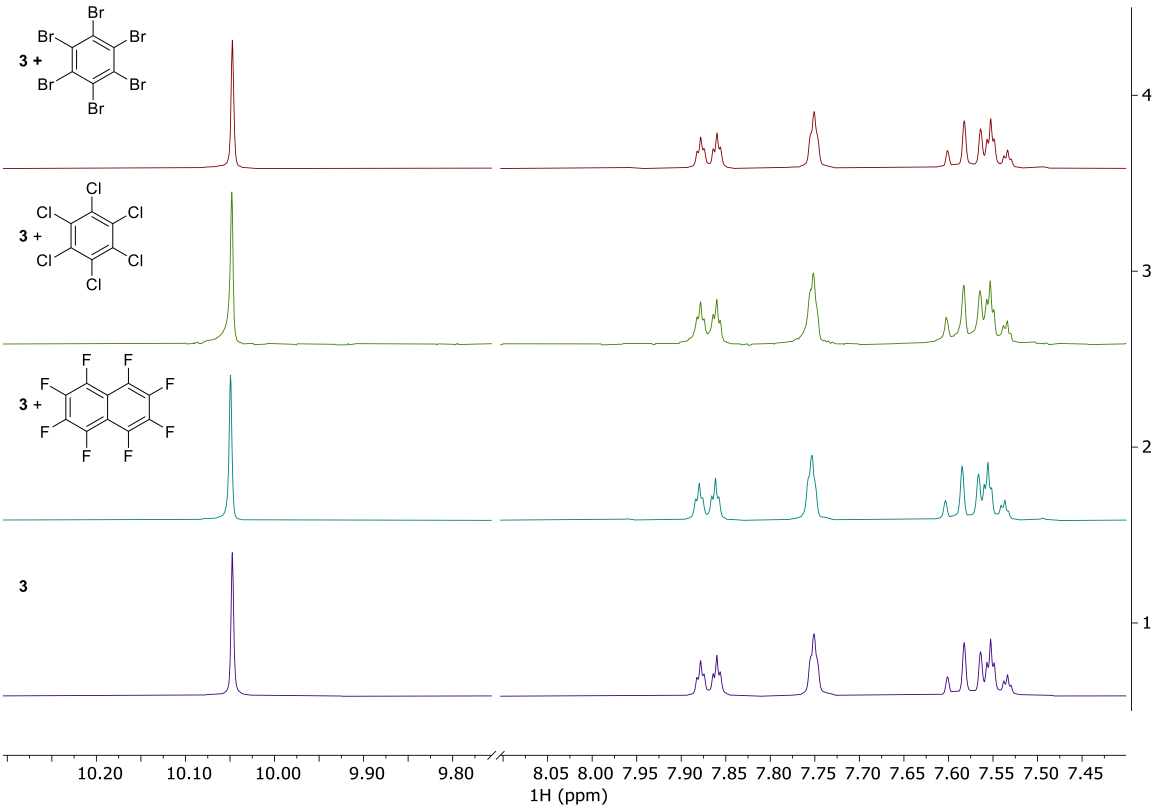


**Figure S55.** $^{1}$H-NMR spectra (400 MHz, $CD_2Cl_2$, 298 K) of complex **3** (0.5 mM) and of complex **3** with octafluoronaphthalene (5 mM), with hexachlorobenzene (5 mM), or with hexabromobenzene (5 mM). An interaction between complex **3** and the halogenated compounds cannot be detected.

# 4 Crystallographic Data

## 4.1 Crystal structure of 3

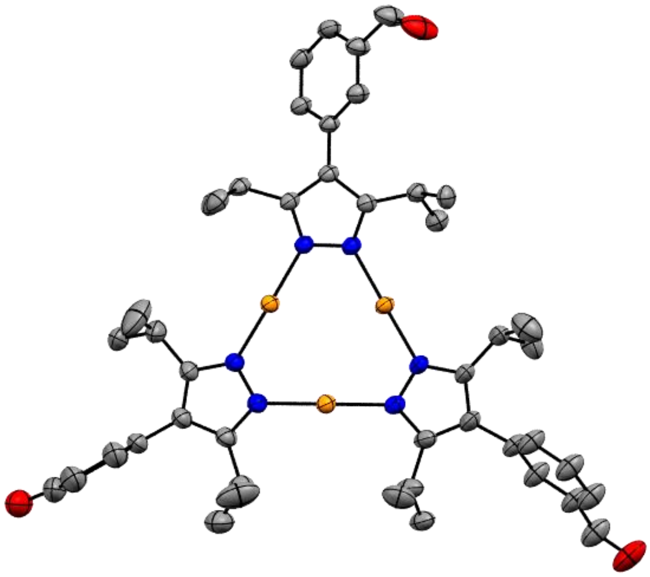

**Figure S56.** OTRP plot at 50% probability of **3**; hydrogen atoms are omitted for clarity.

Crystals suitable for SCXRD analysis were obtained by layering acetonitrile onto a solution of complex **3** in DCM.

A colorless needle-shaped crystal with dimensions 0.41 × 0.05 × 0.04 $mm^3$ was mounted. Data were collected using an XtaLAB Synergy R, DW system, HyPix-Arc 150 diffractometer operating at $T$ = 140.00(10) K.

Data were measured using *w* scans with Mo$K\alpha$ radiation. The diffraction pattern was indexed and the total number of runs and images was based on the strategy calculation from the program CrysAlis$^{Pro}$.[3] The maximum resolution achieved was $\Theta$ = 30.508° (0.7 Å).

The unit cell was refined using CrysAlis$^{Pro}$ on 18163 reflections, 34% of the observed reflections.

Data reduction, scaling and absorption corrections were performed using CrysAlis$^{Pro}$. The final completeness is 100.00 % out to 30.508° in $\Theta$. A Gaussian absorption correction was performed using CrysAlis$^{Pro}$. Numerical absorption correction based on Gaussian integration over a multifaceted crystal model. Empirical absorption correction using spherical harmonics as implemented in SCALE3 ABSPACK scaling algorithm. The absorption coefficient $\mu$ of this material is 9.414 $mm^{-1}$ at this wavelength ($\lambda$ = 0.71073Å) and the minimum and maximum transmissions are 0.361 and 0.832.

The structure was solved in the space group $P2_1/c$ (# 14) by the ShelXT[4] structure solution program using dual methods and refined by full matrix least squares minimization on $\boldsymbol{F^2}$ using version 2019/3 of ShelXL.[5] All non-hydrogen atoms were refined anisotropically. Hydrogen atom positions were calculated geometrically and

refined using the riding model. There are neither A- nor B-level alerts in the check CIF report.

There is a single formula unit in the asymmetric unit, which is represented by the reported sum formula. In other words: *Z* is 4 and *Z*' is 1.

**Table S1.** Crystal data and structure refinement for **3**

| | |
|---|---|
| Formula | $C_{48}H_{57}Au_3N_6O_3$ |
| $D_{calc.}$/ g cm$^{-3}$ | 1.923 |
| $\mu$/mm$^{-1}$ | 9.414 |
| Formula Weight | 1356.89 |
| Color | colorless |
| Shape | needle-shaped |
| Size/mm$^3$ | 0.41×0.05×0.04 |
| *T*/K | 140.00(10) |
| Crystal System | monoclinic |
| Space Group | $P2_1/c$ |
| *a*/Å | 20.0056(4) |
| *b*/Å | 29.2892(6) |
| *c*/Å | 8.03827(16) |
| $\alpha$/° | 90 |
| $\beta$/° | 95.696(2) |
| $\gamma$/° | 90 |
| V/Å$^3$ | 4686.76(17) |
| *Z* | 4 |
| *Z'* | 1 |
| Wavelength/Å | 0.71073 |
| Radiation type | Mo$K\alpha$ |
| $\Theta_{min}$/° | 2.046 |
| $\Theta_{max}$/° | 30.508 |
| Measured Refl's. | 54089 |
| Indep't Refl's | 14329 |
| Refl's I≥2$\sigma$(I) | 10387 |
| $R_{int}$ | 0.0378 |
| Parameters | 643 |
| Restraints | 564 |
| Largest Peak/e Å$^{-3}$ | 1.569 |
| Deepest Hole/e Å$^{-3}$ | –1.494 |
| GooF | 0.998 |
| $wR_2$ (all data) | 0.0575 |
| $wR_2$ | 0.0519 |
| $R_1$ (all data) | 0.0558 |
| $R_1$ | 0.0295 |
| *CCDC number* | 2284420 |

## 4.2 Crystal structure of 4

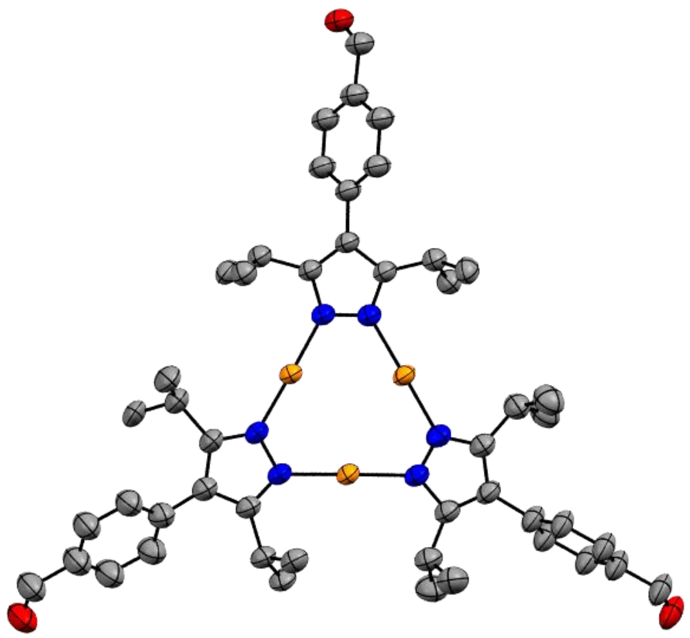

**Figure S57.** OTRP plot at 50% probability of **4**; hydrogen atoms are omitted for clarity.

Crystals suitable for SCXRD analysis were obtained by layering MeOH onto a solution of complex **4** in $CHCl_3$.

A colorless needle-shaped crystal with dimensions 0.41 × 0.01 × 0.01 $mm^3$ was mounted. Data were collected using an XtaLAB Synergy R, DW system, HyPix-Arc 150 diffractometer operating at $T$ = 229.99(10) K.

Data were measured using $\omega$ scans with CuK$\alpha$ radiation. The diffraction pattern was indexed and the total number of runs and images was based on the strategy calculation from the program CrysAlis$^{Pro}$. The maximum resolution achieved was $\Theta$ = 66.601° (0.84 Å).

The unit cell was refined using CrysAlis$^{Pro}$ 1.171.42.90a (Rigaku OD, 2023) on 9869 reflections, 29% of the observed reflections.

Data reduction, scaling and absorption corrections were performed using CrysAlis$^{Pro}$ 1.171.42.90a (Rigaku OD, 2023). The final completeness is 99.40 % out to 66.601° in $\Theta$. A Gaussian absorption correction was performed using CrysAlis$^{Pro}$ 1.171.42.90a (Rigaku Oxford Diffraction, 2023). Numerical absorption correction based on Gaussian integration over a multifaceted crystal model. Empirical absorption correction using spherical harmonics as implemented in SCALE3 ABSPACK scaling algorithm. The absorption coefficient $\mu$ of this material is 17.334 $mm^{-1}$ at this wavelength ($\lambda$ = 1.54184Å) and the minimum and maximum transmissions are 0.140 and 1.000.

The structure was solved in the space group $P\bar{1}$ (# 2) by the ShelXT 2018/2 (Sheldrick, 2015) structure solution program using dual methods and refined by full matrix least squares minimization on $\boldsymbol{F^2}$ using version 2019/3 of ShelXL (Sheldrick, 2015). All non-

hydrogen atoms were refined anisotropically. Hydrogen atom positions were calculated geometrically and refined using the riding model. A RIGU card was applied to all the atoms since the entire structure is disordered. SIMU, SADI and DFIX cards were imposed on the atoms, which are split over two positions. There are neither A- nor B-level alerts in the check CIF report.

There is a single formula unit in the asymmetric unit, which is represented by the reported sum formula. In other words: *Z* is 2 and *Z*' is 1. The moiety formula is $C_{48}H_{57}Au_3N_6O_3$, 1.25[$CH_2Cl_2$].

A solvent mask was calculated, and 101 electrons were found in a volume of 303 Å$^3$ in two voids per unit cell. This is consistent with the presence of 1.25 solvent molecules of DCM per Asymmetric Unit which account for 105 electrons per unit cell.

**Table S2.** Crystal data and structure refinement for **4**

| | |
|---|---|
| Formula | $C_{49.25}H_{59.5}Au_3Cl_{2.5}N_6O_3$ |
| $D_{calc.}$/ g cm$^{-3}$ | 1.891 |
| $\mu$/mm$^{-1}$ | 17.334 |
| Formula Weight | 1463.05 |
| Color | colorless |
| Shape | needle-shaped |
| Size/mm$^3$ | 0.41×0.01×0.01 |
| $T$/K | 229.99(10) |
| Crystal System | triclinic |
| Space Group | $P\bar{1}$ |
| $a$/Å | 8.3005(6) |
| $b$/Å | 17.0876(10) |
| $c$/Å | 19.6636(9) |
| $\alpha$/° | 68.300(5) |
| $\beta$/° | 87.800(5) |
| $\gamma$/° | 82.613(5) |
| V/Å$^3$ | 2569.7(3) |
| $Z$ | 2 |
| $Z'$ | 1 |
| Wavelength/Å | 1.54184 |
| Radiation type | Cu$K\alpha$ |
| $\Theta_{min}$/° | 2.418 |
| $\Theta_{max}$/° | 66.601 |
| Measured Refl's. | 34529 |
| Indep't Refl's | 9020 |
| Refl's I≥2$\sigma$(I) | 5489 |
| $R_{int}$ | 0.0979 |
| Parameters | 568 |
| Restraints | 649 |
| Largest Peak/e Å$^{-3}$ | 1.914 |
| Deepest Hole/e Å$^{-3}$ | -2.617 |
| GooF | 1.011 |
| $wR_2$ (all data) | 0.1994 |
| $wR_2$ | 0.1546 |
| $R_1$ (all data) | 0.1083 |
| $R_1$ | 0.0609 |
| *CCDC number* | 2284422 |

## 4.3 Crystal structure of 5

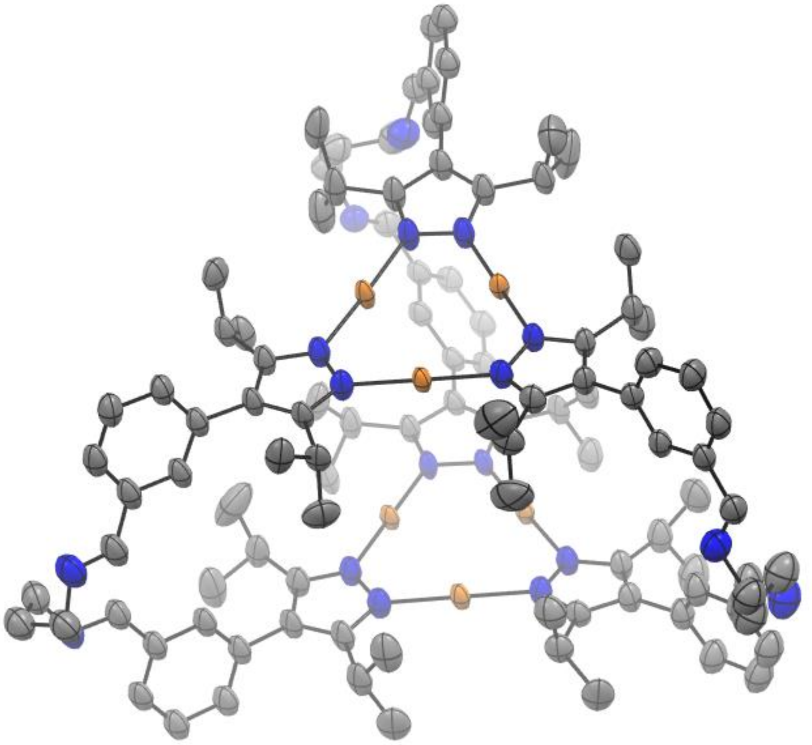

**Figure S58.** OTRP plot at 50% probability of **5**; hydrogen atoms are omitted for clarity.

Crystals suitable for SCXRD analysis were obtained by layering acetonitrile onto a solution of cage **5** in $CH_2Cl_2$.

A colourless prism-shaped crystal with dimensions 0.10 × 0.05 × 0.03 $mm^3$ was mounted. Data were collected using an XtaLAB Synergy R, DW system, HyPix-Arc 150 diffractometer operating at $T$ = 140.00(10) K.

Data were measured using $\omega$ scans with Cu$K\alpha$ radiation. The diffraction pattern was indexed and the total number of runs and images was based on the strategy calculation from the program CrysAlis$^{Pro}$ system (CCD 43.104a 64-bit (release 22-12-2023)). The maximum resolution achieved was $\Theta$ = 66.601° (0.84 Å).

The unit cell was refined using CrysAlis$^{Pro}$ 1.171.43.100a (Rigaku OD, 2023) on 32375 reflections, 31% of the observed reflections.

Data reduction, scaling and absorption corrections were performed using CrysAlis$^{Pro}$ 1.171.43.100a (Rigaku OD, 2023). The final completeness is 99.60 % out to 66.601° in $\Theta$. A Gaussian absorption correction was performed using CrysAlis$^{Pro}$ 1.171.43.100a (Rigaku Oxford Diffraction, 2023) Numerical absorption correction based on Gaussian integration over a multifaceted crystal model. Empirical absorption correction using spherical harmonics as implemented in SCALE3 ABSPACK scaling algorithm. The absorption coefficient $\mu$ of this material is 15.148 $mm^{-1}$ at this wavelength ($\lambda$ = 1.54184Å) and the minimum and maximum transmissions are 0.385 and 0.787.

The structure was solved in the space group $P\bar{1}$ (# 2) by the ShelXT 2018/2 (Sheldrick, 2015) structure solution program using dual methods and refined by full matrix least squares minimisation on $\boldsymbol{F^2}$ using version 2019/3 of ShelXL 2019/3 (Sheldrick, 2015).

All non-hydrogen atoms were refined anisotropically. Hydrogen atom positions were calculated geometrically and refined using the riding model. RIGU and SIMU cards were applied to all the atoms since the entire structure is disordered. Each gold metal is disordered over three positions with a ratio of 87:8:5, determined by using a SUMP instruction and then fixed. There are neither A- nor B-level alerts in the check CIF report.

There is a single formula unit in the asymmetric unit, which is represented by the reported sum formula. In other words: Z is 2 and Z' is 1. The moiety formula is $C_{105}H_{132}Au_6N_{18}$, 4[$CH_2Cl_2$].

A solvent mask was calculated and 318 electrons were found in a volume of 1368 Å$^3$ in one void per unit cell. This is consistent with the presence of four solvent molecules of DCM per Asymmetric Unit which account for 336 electrons per unit cell.

**Table S3.** Crystal data and structure refinement for **5**

| | |
|---|---|
| Formula | $C_{109}H_{140}Au_6Cl_8N_{18}$ |
| $D_{calc.}$/ g cm$^{-3}$ | 1.716 |
| $\mu$/mm$^{-1}$ | 15.148 |
| Formula Weight | 3167.78 |
| Colour | colourless |
| Shape | prism-shaped |
| Size/mm$^3$ | 0.10×0.05×0.03 |
| *T*/K | 140.00(10) |
| Crystal System | triclinic |
| Space Group | $P\bar{1}$ |
| *a*/Å | 15.6340(3) |
| *b*/Å | 17.1978(2) |
| *c*/Å | 25.4548(4) |
| $\alpha$/° | 94.8137(12) |
| $\beta$/° | 107.0463(15) |
| $\gamma$/° | 107.4465(15) |
| V/Å$^3$ | 6130.60(18) |
| *Z* | 2 |
| *Z'* | 1 |
| Wavelength/Å | 1.54184 |
| Radiation type | Cu$K\alpha$ |
| $Q_{min}$/° | 1.848 |
| $Q_{max}$/° | 66.601 |
| Measured Refl's. | 105980 |
| Indep't Refl's | 21590 |
| Refl's I≥2$\sigma$(I) | 16251 |
| $R_{int}$ | 0.0674 |
| Parameters | 1293 |
| Restraints | 2281 |
| Largest Peak/e Å$^{-3}$ | 1.430 |
| Deepest Hole/e Å$^{-3}$ | -1.494 |
| GooF | 1.047 |
| $wR_2$ (all data) | 0.1184 |
| $wR_2$ | 0.1119 |
| $R_1$ (all data) | 0.0620 |
| $R_1$ | 0.0446 |
| *CCDC number* | 2284421 |

## 4.4 Crystal structure of 6

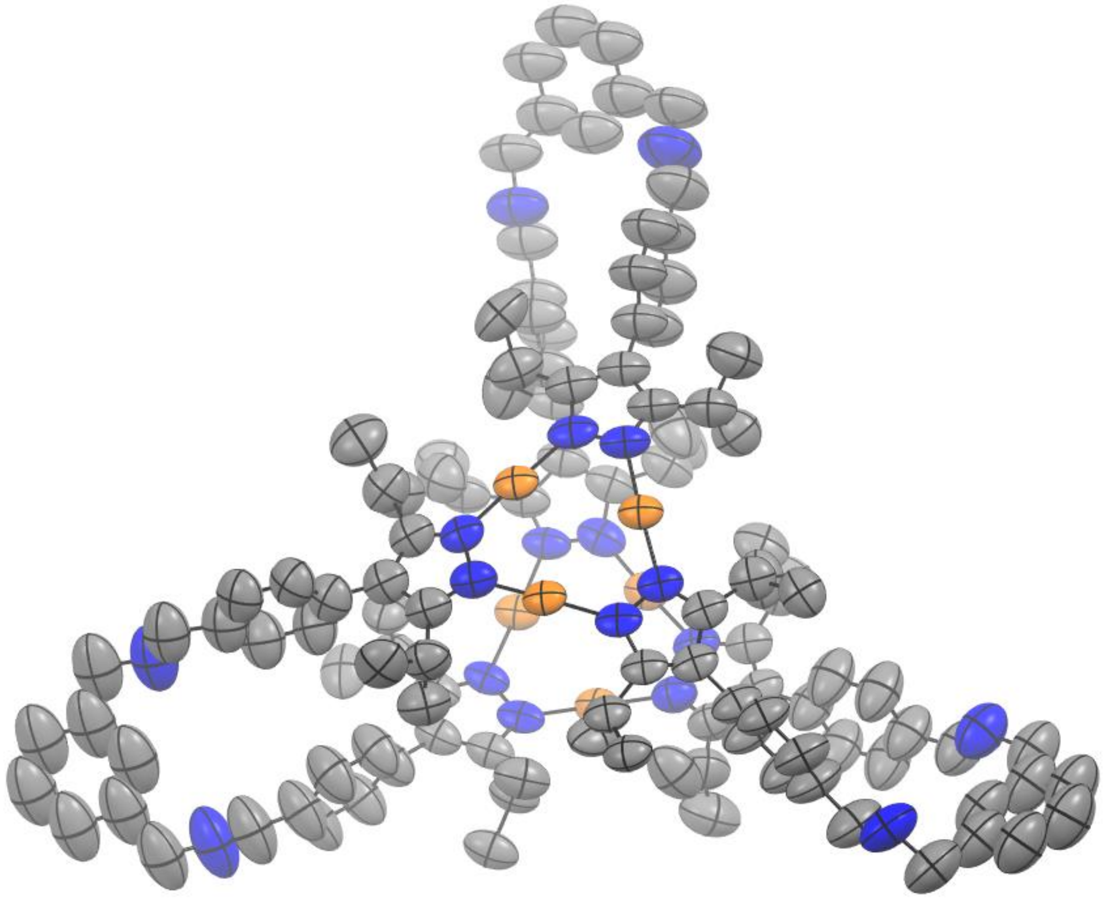

**Figure S59.** OTRP plot at 50% probability of **6**; hydrogen atoms are omitted for clarity.

Crystals suitable for SCXRD analysis were obtained from a mixture of complex **4** and *m*-xylylene diamine in $CDCl_3$.

A colorless needle-shaped crystal with dimensions 0.53 × 0.03 × 0.02 $mm^3$ was mounted. Data were collected using an XtaLAB Synergy R, DW system, HyPix-Arc 150 diffractometer operating at $T$ = 140.00 K.

Data were measured using $\omega$ scans with CuK$\alpha$ radiation. The diffraction pattern was indexed and the total number of runs and images was based on the strategy calculation from the program CrysAlis$^{Pro}$ 1.171.42.85a (Rigaku OD, 2023). The maximum resolution achieved was $\Theta$ = 50.436° (1.00 Å).

The unit cell was refined using CrysAlis$^{Pro}$ 1.171.42.85a (Rigaku OD, 2023) on 30479 reflections, 32% of the observed reflections.

Data reduction, scaling and absorption corrections were performed using CrysAlis$^{Pro}$ 1.171.42.85a (Rigaku OD, 2023). The final completeness is 99.70 % out to 50.436° in $\Theta$. A Gaussian absorption correction was performed using CrysAlis$^{Pro}$ 1.171.42.85a (Rigaku Oxford Diffraction, 2023). Numerical absorption correction based on Gaussian integration over a multifaceted crystal model. Empirical absorption correction using spherical harmonics as implemented in SCALE3 ABSPACK scaling algorithm. The

absorption coefficient $\mu$ of this material is 12.743 $mm^{-1}$ at this wavelength ($\lambda$ = 1.54184Å) and the minimum and maximum transmissions are 0.214 and 1.000.

The structure was solved in the space group $P\bar{1}$ (# 2) by the ShelXT 2018/2 (Sheldrick, 2015) structure solution program using dual methods and refined by full matrix least squares minimization on $\boldsymbol{F^2}$ using version 2019/3 of ShelXL 2019/3 (Sheldrick, 2015). All non-hydrogen atoms were refined anisotropically. Hydrogen atom positions were calculated geometrically and refined using the riding model. A RIGU card was applied to all the atoms since the entire structure is disordered. Soft SIMU, SADI, DFIX and DANG cards were imposed on some atoms to keep the right chemical geometry. The check CIF report shows only one A-level alert concerning the low data resolution, since there were no reflections beyond d(min)= 1 Å.

*_exptl_absorpt_special_details:* There were no reflections beyond d(min)= 1 Å. So, data are cut to this resolution.

There is a single formula unit in the asymmetric unit, which is represented by the reported sum formula. In other words: *Z* is 2 and *Z*' is 1. The moiety formula is $C_{120}H_{138}Au_6N_{18}$, 11.5[$CH_2Cl_2$], 1.25[$CH_2Cl_2$].

**Table S4.** Crystal data and structure refinement for **6**

| | |
|---|---|
| Formula | $C_{132.75}H_{163.5}Au_6Cl_{25.5}N_{18}$ |
| $D_{calc.}$/ g cm$^{-3}$ | 1.506 |
| $\mu$/mm$^{-1}$ | 12.743 |
| Formula Weight | 4097.08 |
| Color | colorless |
| Shape | needle-shaped |
| Size/mm$^3$ | 0.53×0.03×0.02 |
| $T$/K | 140.00 |
| Crystal System | triclinic |
| Space Group | $P\bar{1}$ |
| $a$/Å | 20.8869(6) |
| $b$/Å | 21.3091(7) |
| $c$/Å | 21.4286(4) |
| $\alpha$/° | 96.246(2) |
| $\beta$/° | 97.033(2) |
| $\gamma$/° | 105.196(3) |
| V/Å$^3$ | 9035.4(5) |
| $Z$ | 2 |
| $Z'$ | 1 |
| Wavelength/Å | 1.54184 |
| Radiation type | Cu$K\alpha$ |
| $\Theta_{min}$/° | 2.220 |
| $\Theta_{max}$/° | 50.436 |
| Measured Refl's. | 94655 |
| Indep't Refl's | 18871 |
| Refl's I≥2$\sigma$(I) | 12121 |
| $R_{int}$ | 0.0771 |
| Parameters | 1210 |
| Restraints | 2253 |
| Largest Peak/e Å$^{-3}$ | 1.438 |
| Deepest Hole/e Å$^{-3}$ | -0.909 |
| GooF | 1.053 |
| $wR_2$ (all data) | 0.1671 |
| $wR_2$ | 0.1577 |
| $R_1$ (all data) | 0.0780 |
| $R_1$ | 0.0536 |
| *CCDC number* | 2284423 |

## 4.5 Crystal structure of 7

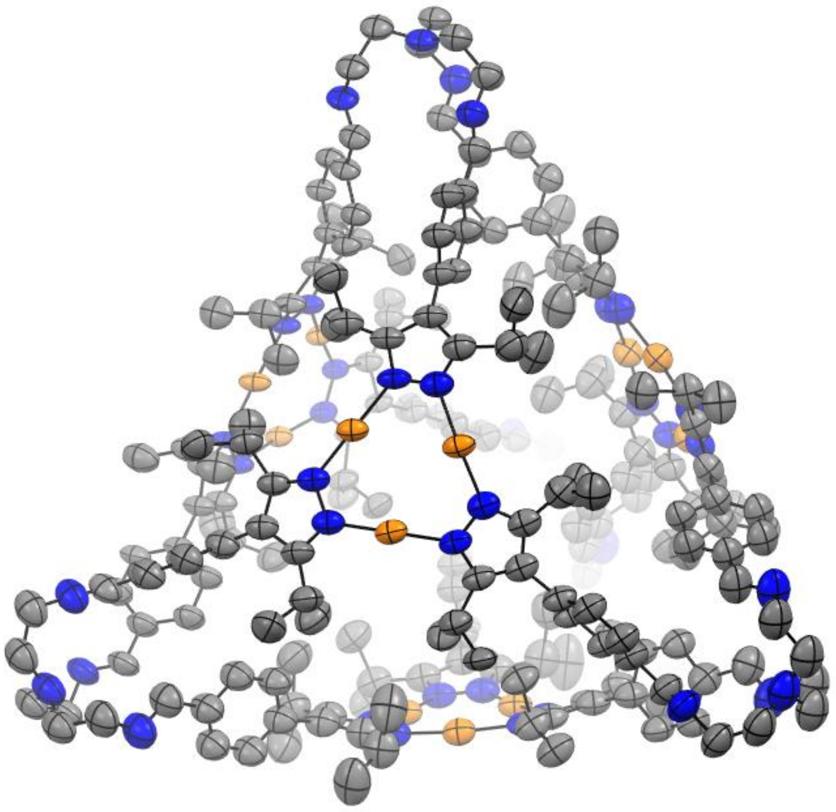

**Figure S60.** OTRP plot at 50% probability of **7**; hydrogen atoms are omitted for clarity.

Crystals suitable for SCXRD analysis were obtained by vapor diffusion of diisopropyl ether into a solution of **7** in 1,1,2,2-tetrachloroethene.

A colorless prism-shaped crystal with dimensions 0.47 × 0.25 × 0.12 $mm^3$ was mounted. Data were collected using an XtaLAB Synergy R, DW system, HyPix-Arc 150 diffractometer operating at $T$ = 229.99(10) K.

Data were measured using $\omega$ scans with Cu$K\alpha$ radiation. The diffraction pattern was indexed and the total number of runs and images was based on the strategy calculation from the program CrysAlis$^{Pro}$ 1.171.42.90a (Rigaku OD, 2023). The maximum resolution achieved was $\Theta$ = 66.599° (0.84 Å).

The unit cell was refined using CrysAlis$^{Pro}$ 1.171.42.90a (Rigaku OD, 2023) on 75502 reflections, 28% of the observed reflections.

Data reduction, scaling and absorption corrections were performed using CrysAlis$^{Pro}$ 1.171.42.90a (Rigaku OD, 2023). The final completeness is 99.50 % out to 66.599° in $\Theta$. A Gaussian absorption correction was performed using CrysAlis$^{Pro}$ 1.171.42.90a (Rigaku Oxford Diffraction, 2023). Numerical absorption correction based on Gaussian integration over a multifaceted crystal model. Empirical absorption correction using spherical harmonics as implemented in SCALE3 ABSPACK scaling algorithm. The absorption coefficient $\mu$ of this material is 13.966 $mm^{-1}$ at this wavelength ($\lambda$ =

1.54184 Å) and the minimum and maximum transmissions are 0.065 and 0.862.

The structure was solved in the space group $P\bar{1}$ (# 2) by the ShelXT 2018/2 (Sheldrick, 2015) structure solution program using dual methods and refined by full matrix least squares minimization on $\boldsymbol{F^2}$ using version 2019/3 of ShelXL 2019/3 (Sheldrick, 2015). All non-hydrogen atoms were refined anisotropically. Hydrogen atom positions were calculated geometrically and refined using the riding model. A RIGU card was applied to all the atoms since the entire structure is disordered. Soft SIMU, DFIX and DANG cards were imposed on some atoms to keep the right chemical geometry. The check CIF report shows two B-level alerts concerning a low bond precision on the C-C bonds and a residual density of 2.6 eÅ$^{-3}$ around the gold atoms. Both these alerts were commented in the CIF.

There is a single formula unit in the asymmetric unit, which is represented by the reported sum formula. In other words: *Z* is 2 and *Z*' is 1. The moiety formula is $C_{216}H_{276}Au_{12}N_{40}$, 12.75[$C_2H_2Cl_4$].

A solvent mask was calculated, and 2010 electrons were found in a volume of 7652 Å3 in two voids per unit cell. This is consistent with the presence of 12.75 solvent molecules of tetrachloroethane per asymmetric unit which account for 2040 electrons per unit cell.

**Table S5.** Crystal data and structure refinement for **7**

| | |
|---|---|
| Formula | $C_{241.5}H_{301.5}Au_{12}Cl_{51}N_{40}$ |
| $D_{calc.}$/ g cm$^{-3}$ | 1.595 |
| $\mu$/mm$^{-1}$ | 13.966 |
| Formula Weight | 7910.55 |
| Colour | colourless |
| Shape | prism-shaped |
| Size/mm$^3$ | 0.47×0.25×0.12 |
| $T$/K | 229.99(10) |
| Crystal System | triclinic |
| Space Group | $P\bar{1}$ |
| $a$/Å | 27.6564(2) |
| $b$/Å | 27.7466(3) |
| $c$/Å | 28.2657(3) |
| $\alpha$/° | 112.4515(10) |
| $\beta$/° | 94.9615(8) |
| $\gamma$/° | 119.2080(10) |
| V/Å$^3$ | 16466.2(3) |
| $Z$ | 2 |
| $Z'$ | 1 |
| Wavelength/Å | 1.54184 |
| Radiation type | Cu$K\alpha$ |
| $\Theta_{min}$/° | 1.884 |
| $\Theta_{max}$/° | 66.599 |
| Measured Refl's. | 265630 |
| Indep't Refl's | 57857 |
| Refl's I≥2$\sigma$(I) | 40389 |
| $R_{\text{int}}$ | 0.0875 |
| Parameters | 2456 |
| Restraints | 3918 |
| Largest Peak/e Å$^{-3}$ | 2.751 |
| Deepest Hole/e Å$^{-3}$ | -2.101 |
| GooF | 1.054 |
| $wR_2$ (all data) | 0.2050 |
| $wR_2$ | 0.1956 |
| $R_1$ (all data) | 0.0863 |
| $R_1$ | 0.0684 |
| *CCDC number* | 2284424 |

## 4.6 Crystal structure of $C_{60}\subset$7

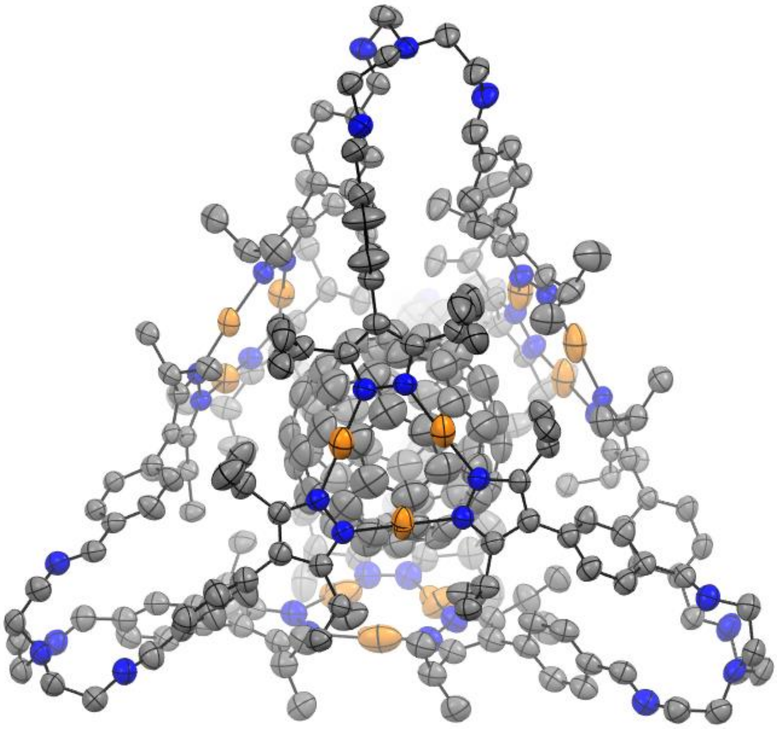

**Figure S61.** OTRP plot at 50% probability of **$C_{60}\subset$7**; hydrogen atoms and solvent molecules are omitted for clarity.

Crystals suitable for SCXRD analysis were obtained by vapor diffusion of diethyl ether into a solution of **$C_{60}\subset$7** in $CDCl_3$.

A clear intense red plate-shaped crystal with dimensions 0.66 × 0.49 × 0.20 $mm^3$ was mounted. Data were collected using a XtaLAB Synergy R, DW system, HyPix-Arc 150 diffractometer operating at $T$ = 140.15 K.

Data were measured using $\omega$ scans with Cu $K_\alpha$ radiation. The diffraction pattern was indexed and the total number of runs and images was based on the strategy calculation from the program $CrysAlis^{Pro}$. The maximum resolution that was achieved was $\Theta$ = 74.971° (0.80 Å).

The unit cell was refined using $CrysAlis^{Pro}$ on 93630 reflections, 46% of the observed reflections.

Data reduction, scaling and absorption corrections were performed using $CrysAlis^{Pro}$. The final completeness is 99.50 % out to 74.971° in $\Theta$. A gaussian absorption correction was performed using $CrysAlis^{Pro}$. The numerical absorption correction was based on Gaussian integration over a multifaceted crystal model. The empirical absorption correction was done using spherical harmonics, implemented in SCALE3 ABSPACK scaling algorithm. The absorption coefficient $\mu$ of this crystal is 13.022 $mm^{-1}$ at this wavelength ($\lambda$ = 1.54184Å) and the minimum and maximum transmissions are 0.009 and 0.599.

The structure was solved and the space group $P$-1 (# 2) determined by the ShelXT

structure solution program using dual methods and refined by full matrix least squares minimization on $\boldsymbol{F^2}$ using version 2019/3 of ShelXL. All non-hydrogen atoms were refined anisotropically. Hydrogen atom positions were calculated geometrically and refined using the riding model. The check CIF report shows one B-level alert, the alert was commented in the CIF.

*_smtbx_masks_special_details*: A solvent mask was calculated and 618 electrons were found in a volume of 2598 Å$^3$ in 5 voids per unit cell. This is consistent with the presence of 2[$CDCl_3$] per asymmetric unit which account for 652 electrons per unit cell.

There is a single formula unit in the asymmetric unit, which is represented by the reported sum formula. In other words: *Z* is 2 and *Z*' is 1. The moiety formula is $C_{216}H_{276}Au_{12}N_{40}$, 2($CDCl_3$), $C_{60}$, 2[$CDCl_3$].

**Table S6.** Crystal data and structure refinement for **$C_{60}$⊂7**

| | |
|---|---|
| Formula | $C_{280}H_{276}Au_{12}Cl_{12}D_4N_{40}$ |
| $D_{calc.}$/ g cm$^{-3}$ | 1.663 |
| $\mu$/mm$^{-1}$ | 13.022 |
| Formula Weight | 6998.45 |
| Colour | clear intense red |
| Shape | plate-shaped |
| Size/mm$^3$ | 0.66×0.49×0.20 |
| $T$/K | 140.15 |
| Crystal System | triclinic |
| Space Group | $P$-1 |
| $a$/Å | 22.16590(19) |
| $b$/Å | 24.2178(2) |
| $c$/Å | 26.9851(2) |
| $\alpha$/° | 91.9058(7) |
| $\beta$/° | 104.0920(8) |
| $\gamma$/° | 94.9457(7) |
| V/Å$^3$ | 13975.5(2) |
| $Z$ | 2 |
| $Z'$ | 1 |
| Wavelength/Å | 1.54184 |
| Radiation type | Cu $K_\alpha$ |
| $\Theta_{min}$/° | 2.323 |
| $\Theta_{max}$/° | 74.971 |
| Measured Refl's. | 204256 |
| Indep't Refl's | 54774 |
| Refl's I≥2 $\sigma$(I) | 46601 |
| $R_{int}$ | 0.0614 |
| Parameters | 3131 |
| Restraints | 7430 |
| Largest Peak | 2.366 |
| Deepest Hole | –2.133 |
| GooF | 1.045 |
| $wR_2$ (all data) | 0.1693 |
| $wR_2$ | 0.1644 |
| $R_1$ (all data) | 0.0669 |
| $R_1$ | 0.0598 |
| Formula | 2309725 |

## 4.7 Crystal structure of $C_{70}$⊂**7**

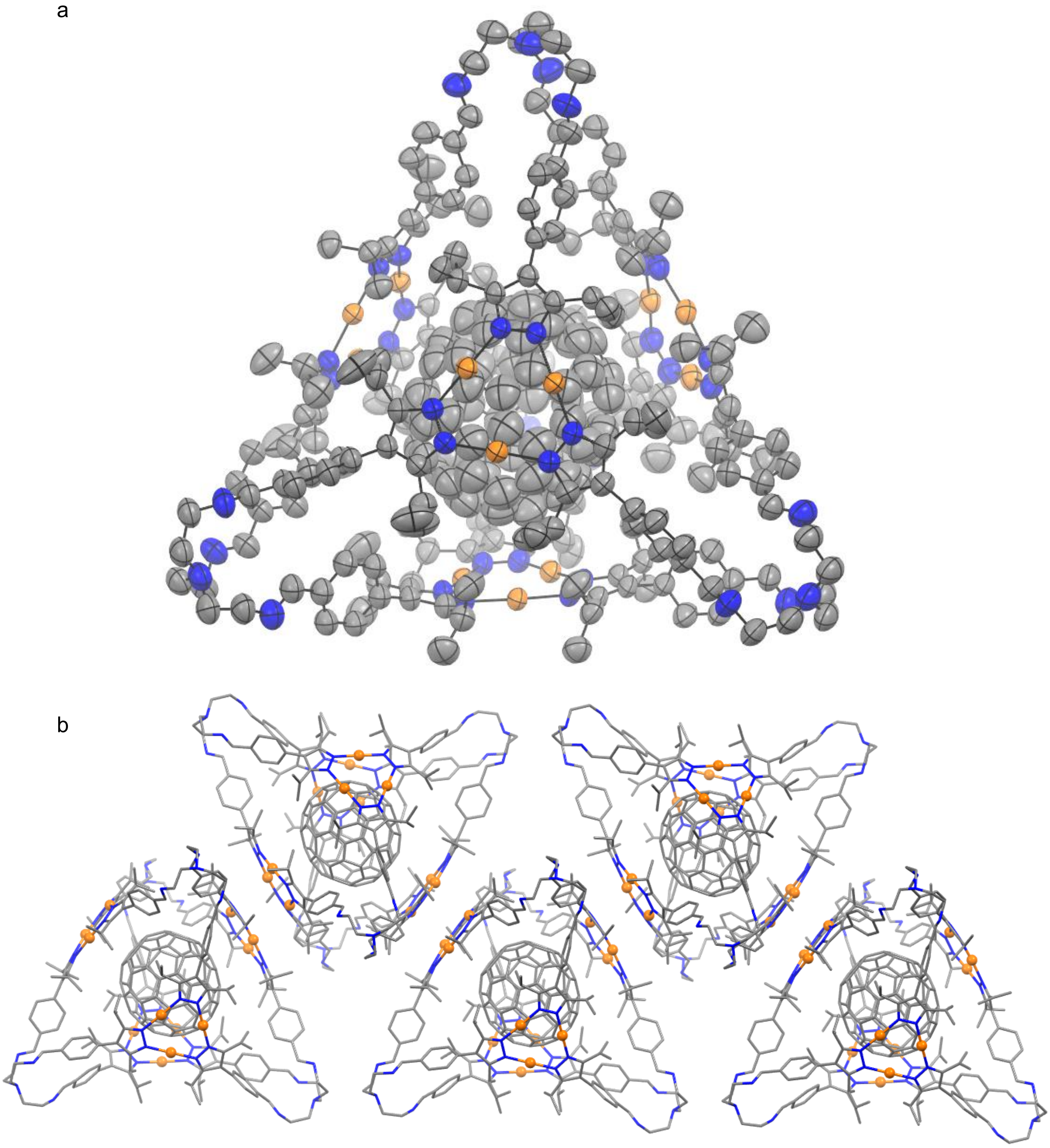


**Figure S62.** a) OTRP plot at 50% probability of **$C_{70}$⊂7**; hydrogen atoms are omitted for clarity. B) Packing of **$C_{70}$⊂7.**

Crystals suitable for SCXRD analysis were obtained by vapor diffusion of diisopropyl ether into a solution of **$C_{70}$⊂7** in 1,1,2,2-tetrachloroethene.

A clear dark red prism-shaped crystal with dimensions 0.25 × 0.21 × 0.16 $mm^3$ was mounted. Data were collected using an XtaLAB Synergy R, DW system, HyPix-Arc 150 diffractometer operating at $T$ = 229.99(10) K.

Data were measured using $\omega$ scans with CuK$\alpha$ radiation. The diffraction pattern was indexed and the total number of runs and images was based on the strategy calculation from the program CrysAlis$^{Pro}$ 1.171.42.90a (Rigaku OD, 2023). The maximum resolution achieved was $\Theta$ = 66.600° (0.84 Å).

The unit cell was refined using CrysAlis$^{Pro}$ 1.171.42.90a (Rigaku OD, 2023) on 78413 reflections, 26% of the observed reflections.

Data reduction, scaling and absorption corrections were performed using CrysAlis$^{Pro}$ 1.171.42.90a (Rigaku OD, 2023). The final completeness is 99.50 % out to 66.600° in $\Theta$. A Gaussian absorption correction was performed using CrysAlis$^{Pro}$ 1.171.42.90a (Rigaku Oxford Diffraction, 2023). Numerical absorption correction based on Gaussian integration over a multifaceted crystal model. Empirical absorption correction using spherical harmonics as implemented in SCALE3 ABSPACK scaling algorithm. The absorption coefficient $\mu$ of this material is 13.495 mm$^{-1}$ at this wavelength ($\lambda$ = 1.54184Å) and the minimum and maximum transmissions are 0.198 and 0.513.

The structure was solved in the space group $P\bar{1}$ (# 2) by the ShelXT 2018/2 (Sheldrick, 2015) structure solution program using dual methods and refined by full matrix least squares minimization on $\boldsymbol{F^2}$ using version 2019/3 of ShelXL 2019/3 (Sheldrick, 2015). All non-hydrogen atoms were refined anisotropically. Hydrogen atom positions were calculated geometrically and refined using the riding model. RIGU and SIMU cards were applied to all the atoms since the entire structure is disordered. AFIX 66, SADI and DFIX cards were imposed on some atoms to keep the right chemical geometry. There are neither A- nor B-level alerts in the check CIF report.

There is a single formula unit in the asymmetric unit, which is represented by the reported sum formula. In other words: *Z* is 2 and *Z*' is 1. The moiety formula is $C_{216}H_{272}Au_{12}N_{40}$, $C_{70}$, 13[$C_2H_2Cl_4$].

A solvent mask was calculated, and 2078 electrons were found in a volume of 6808 Å$^3$ in two voids per unit cell. This is consistent with the presence of 13 solvent molecules of tetrachloroethane per asymmetric unit which account for 2080 electrons per unit cell.

**Table S7.** Crystal data and structure refinement for **$C_{70}$⊂7**

| | |
|---|---|
| Formula | $C_{312}H_{298}Au_{12}Cl_{52}N_{40}$ |
| $D_{calc.}$/ g cm$^{-3}$ | 1.694 |
| $\mu$/mm$^{-1}$ | 13.495 |
| Formula Weight | 8788.68 |
| Colour | clear dark red |
| Shape | prism-shaped |
| Size/mm$^3$ | 0.25×0.21×0.16 |
| *T*/K | 229.99(10) |
| Crystal System | triclinic |
| Space Group | $P\bar{1}$ |
| *a*/Å | 27.2311(3) |
| *b*/Å | 27.3567(3) |
| *c*/Å | 27.4198(4) |
| $\alpha$/° | 62.4166(13) |
| $\beta$/° | 79.9283(11) |
| $\gamma$/° | 72.2345(9) |
| V/Å$^3$ | 17227.8(4) |
| *Z* | 2 |
| *Z'* | 1 |
| Wavelength/Å | 1.54184 |
| Radiation type | Cu$K\alpha$ |
| $\Theta_{min}$/° | 1.705 |
| $\Theta_{max}$/° | 66.600 |
| Measured Refl's. | 297732 |
| Indep't Refl's | 60611 |
| Refl's I≥2$\sigma$(I) | 42884 |
| $R_{int}$ | 0.0425 |
| Parameters | 2957 |
| Restraints | 5711 |
| Largest Peak/e Å$^{-3}$ | 1.929 |
| Deepest Hole/e Å$^{-3}$ | -0.976 |
| GooF | 1.060 |
| $wR_2$ (all data) | 0.1738 |
| $wR_2$ | 0.1642 |
| $R_1$ (all data) | 0.0751 |
| $R_1$ | 0.0570 |
| *CCDC number* | 2284425 |

## 5 References


[1] M. Saotome, D. Shimizu, A. Itagaki, D. J. Young and K. Fujisawa, *Chem. Lett.*, 2019, **48**, 533–536.

[2] G. Yang and R. G. Raptis, *Inorg. Chem.*, 2003, **42**, 261–263.

[3] CrysAlis$^{Pro}$ Software System, Rigaku Oxford Diffraction, (2021).

[4] G. M. Sheldrick, *Acta Cryst.*, 2015, **A71**, 3–8.

[5] G. M. Sheldrick, *Acta Cryst.*, 2015, **C71**, 3–8.

[6] O. V. Dolomanov, L. J. Bourhis, R. J. Gildea, J. A. K. Howard and H. Puschmann, *J. Appl. Cryst.*, 2009, **42**, 339–341.
